# A novel quinazolinone insulin receptor inhibitor and its synergy with an EGFR inhibitor in glucose‐driven glioblastoma

**DOI:** 10.1002/1878-0261.70274

**Published:** 2026-05-27

**Authors:** Patryk Rurka, Wioleta Cieślik, Wojciech Płaziński, Katarzyna Stępnik, Anna Boguszewska‐Czubara, Elżbieta Kot, Robert Musioł, Mateusz Jasica, Anna Mrozek‐Wilczkiewicz, Katarzyna Malarz

**Affiliations:** ^1^ Institute of Physics University of Silesia in Katowice Chorzów Poland; ^2^ Institute of Chemistry University of Silesia in Katowice Chorzów Poland; ^3^ Jerzy Haber Institute of Catalysis and Surface Chemistry Polish Academy of Sciences Kraków Poland; ^4^ Department of Biopharmacy Medical University of Lublin Poland; ^5^ Department of Physical Chemistry, Institute of Chemical Sciences Maria Curie–Skłodowska University in Lublin Poland; ^6^ Department of Medical Chemistry Medical University of Lublin Poland; ^7^ Department of Systems Biology and Engineering Silesian University of Technology Gliwice Poland

**Keywords:** anticancer activity, combination therapy, EGFR, glioblastoma, IGF1R kinase, insulin inhibitor, styrylquinazolinone

## Abstract

Aberrant signaling through insulin‐like growth factor 1 receptor (IGF1R) and epidermal growth factor receptor (EGFR) drives glioblastoma (GBM) progression and therapy resistance. Herein, we describe the synthesis and biological evaluation of W1B, a novel styrylquinazolinone‐based small‐molecule inhibitor. In antiproliferative assays, W1B exhibited potent submicromolar activity against a panel of GBM cell lines. Kinase assays and binding studies confirmed strong inhibition and high binding affinity toward IGF1R. Molecular docking suggested possible interactions with both IGF1R and EGFR, with W1B adopting distinct binding poses in each kinase domain. In cellular studies, W1B reduced IGF1R and EGFR protein levels in LN229 cells and suppressed Akt phosphorylation. Under high‐glucose conditions, however, W1B only retained inhibitory activity toward IGF1R, resulting in attenuated effects on the Akt/mTOR axis and underscoring the influence of glucose‐dependent signaling rewiring on drug efficacy. Combination studies revealed that W1B acts synergistically with the EGFR inhibitor dacomitinib, effectively overcoming compensatory activation of parallel pathways. Biomimetic lipophilicity and *in silico* pharmacokinetic analyses indicated that styrylquinazolinone has the potential to cross the blood–brain barrier (BBB). The *in vivo* studies on Danio rerio have shown a good safety profile, as well as strong antitumor potential of the tested compound. Therefore, these findings establish W1B as a promising derivative for the development of next‐generation dual IGF1R/EGFR inhibitors in GBM.

## Graphical abstract

The novel styrylquinazolinone‐based molecule W1B effectively suppresses glioblastoma by inhibiting IGF1R and EGFR. In high‐glucose microenvironments driving tumor resistance, W1B acts synergistically with the EGFR inhibitor dacomitinib. This combination safely blocks compensatory survival signaling in zebrafish xenograft models. Showcasing promising *in silico* blood–brain barrier permeability, W1B represents a valuable lead for developing next‐generation combination therapies against brain tumors.

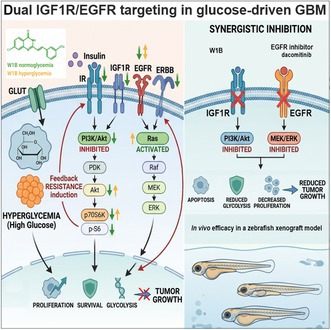

**Abbreviations**
BBB: blood–brain barrier
EGFR: epidermal growth factor receptor
Fb: unbound fractions in brain tissue
Fu: unbound fractions in plasma
GBM: glioblastoma
HPLC: high‐performance liquid chromatography
IAM: immobilized artificial membrane
IGF1R: insulin‐like growth factor 1 receptor
InsR: insulin receptor
logBB: logarithm of blood–brain partitioning
logPcw: logarithm of the cyclohexane/water partition coefficient
logPow: logarithm of the octanol–water partition coefficient
logPS: logarithm of passive diffusion
logPS‐fu, brain: logarithm of brain‐plasma equilibrium
MST: microscale thermophoresis
NSCLC: non‐small cell lung cancer
RTKs: receptor tyrosine kinases
TME: tumor microenvironment
TPSA: topological polar surface area

## 1 Introduction

The insulin‐like growth factor 1 receptor (IGF1R) is a key mediator of signaling pathways that regulate cell growth, proliferation, metabolism, and survival [1]. IGF1R is a transmembrane tyrosine kinase receptor activated by IGF1 and IGF2, which bind to its extracellular domain and initiate autophosphorylation of intracellular tyrosine residues [2]. This leads to the activation of multiple downstream pathways, most notably the PI3K/AKT/mTOR and RAS/RAF/MEK/ERK cascades, as well as Src and JAK/STAT, which are crucial for stimulating cell proliferation, migration and resistance to apoptosis [3]. IGF1R also influences the expression of drug transporters, DNA repair enzymes, and cell cycle regulators [4]. Overexpression or constitutive activation of IGF1R has been observed in various types of cancers, including glioblastoma (GBM), non‐small cell lung cancer (NSCLC), breast cancer, colon cancer, and pancreatic cancer [5]. Beyond tumor cell‐intrinsic mechanisms, IGF1R also shapes the immune landscape of the tumor microenvironment (TME) by driving immunosuppression through STAT3, MAPK, and PI3K signaling that attenuates cytotoxic T‐cell activity [6, 7]. These findings highlight IGF1R as a key driver of tumor progression and immune evasion, supporting the inhibition of this receptor as a therapeutic strategy.

Glioblastoma, the most aggressive and treatment‐resistant primary brain tumor, is characterized by high IGF1R expression and correlates with poor prognosis, reduced survival, and limited responsiveness to standard‐of‐care therapies such as temozolomide [8, 9]. Despite the use of intensive treatment modalities, the median overall survival for GBM patients is only about 10.2 months, and the five‐year survival rate remains below 5% [10]. The development of novel therapeutic approaches has faced numerous obstacles, one of the most critical being efficient drug delivery across the highly selective blood–brain barrier (BBB), which restricts the efficacy of nearly 98% of therapeutic agents [11].

**Fig. 1:**
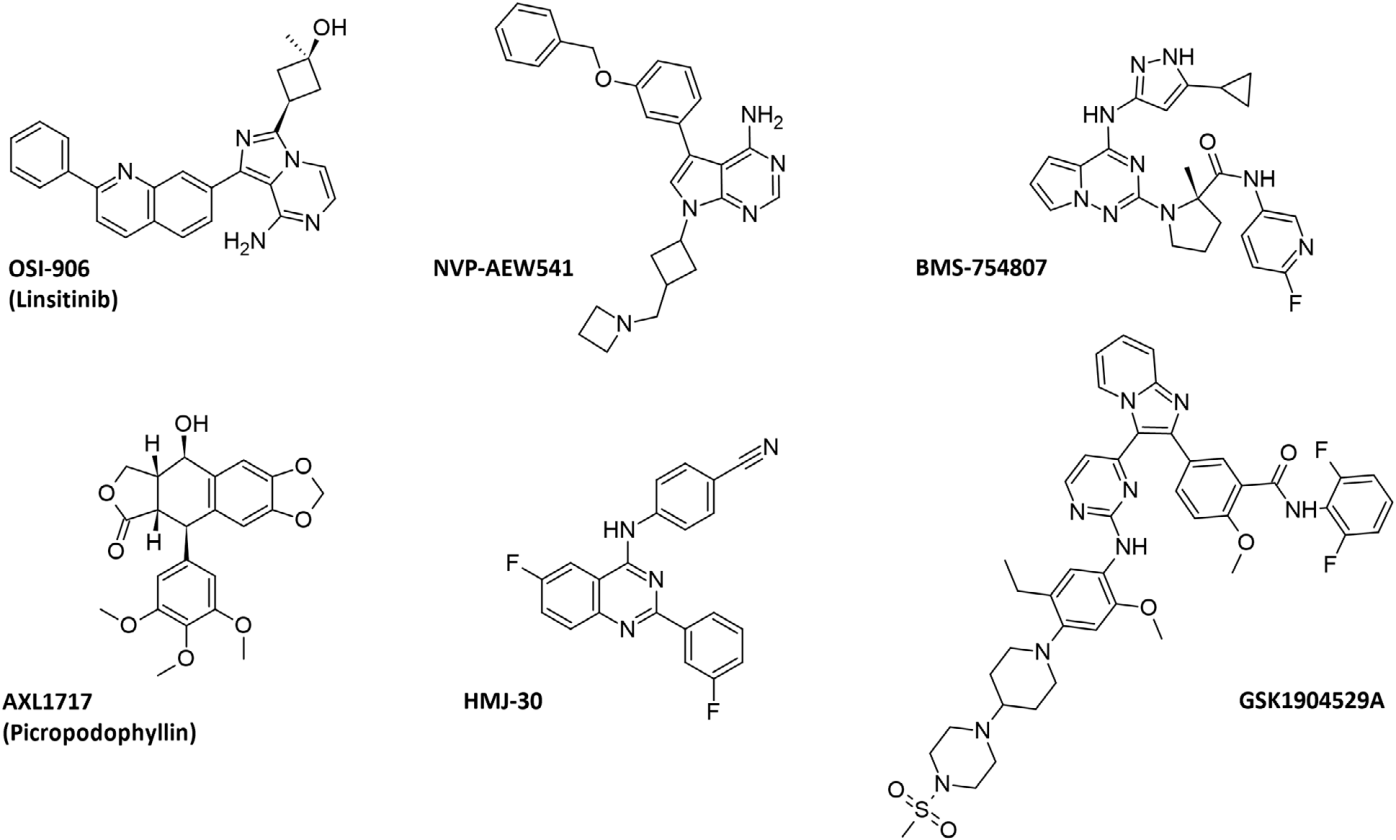
The chemical structures of insulin‐like growth factor 1 receptor (IGF1R) inhibitors.

Although targeted therapies against receptor tyrosine kinases (RTKs), such as epidermal growth factor receptor (EGFR) and IGF1R, have shown promise in preclinical models, clinical translation has been largely unsuccessful [12, 13]. This limited efficacy is mainly attributed to the pronounced molecular heterogeneity of GBM, particularly within the EGFR/PI3K/AKT/mTOR, CDK4/6–CDKN2A/B–RB1 and p53 signaling networks, as well as the emergence of resistance mechanisms, including loss of EGFR expression and activation of compensatory pathways (e.g., c‐MET, IGF1R) [14, 15]. Importantly, similar to other RTKs, IGF1R functions as a central hub within highly integrated signaling networks [16]. Its extensive crosstalk with EGFR [17], integrins [18], and insulin receptor (InsR) [19] has complicated the development of effective IGF1R‐directed therapeutics, while simultaneously providing a rationale for cotargeting strategies. Ultimately, overcoming the resilience of such interconnected signaling systems may require the use of multi‐agent or combination‐based therapeutic approaches. Indeed, combination strategies with OSI‐906 (Linsitinib) (Fig. 1), a dual inhibitor of IGF1R/InsR, and gefitinib, the EGFR inhibitor, showed greater efficacy in treating subcutaneous GBM xenograft tumors. However, this effect may have been counteracted by increased Akt kinase expression [20]. Despite more than 180 clinical trials involving IGF1R‐targeting agents, none have been approved for oncology indications, and only a few have included patients with primary brain tumors [13]. Nonetheless, early clinical data suggest the potential of the small‐molecule AXL1717 (Picropodophyllin) (Fig. 1), which induced a durable response in patients with astrocytoma in a phase I trial [21].

Significant efforts have been directed toward the development of IGF1R inhibitors, encompassing both monoclonal antibodies [22, 23, 24, 25] and small‐molecule tyrosine kinase inhibitors. Notable examples of the latter are NVP‐AEW541 (a pyrrolopyrimidine derivative) [26] and BMS‐754807 (a pyrrolotriazine derivative) [27, 28] (Fig. 1). Quinazoline‐based compounds, such as HMJ‐30 [29] (Fig. 1), along with other derivatives [30], have also been shown to inhibit IGF1R. The quinazoline ring in these molecules underscores its potential as a versatile scaffold for engaging the IGF1R active site, making it an attractive template for the design of next‐generation inhibitors.

Compounds incorporating sulfonyl group (–SO_2_–), including tosyl (4‐methylphenylsulfonyl) moieties, are primarily known for their auxiliary functions in organic synthesis or for modulating physicochemical properties such as solubility and stability—including biochemical stability—while often preserving good bioavailability. Less frequently, they serve as essential pharmacophores in biologically active molecules, and reports on their use as selective kinase inhibitors, including IGF1R inhibitors, remain limited. Notably, GSK1904529A [31] (Fig. 1), a potent and selective IGF1R inhibitor, features a sulfonyl group in its side chain that is critical for interactions with the kinase active site. Moreover, sulfonyl‐indole‐based compounds have demonstrated significant IGF1R inhibitory activity [32]. This underscores the value of sulfonyl moieties as key interaction motifs in the design of IGF1R‐targeted inhibitors. In our group, we have been actively developing compounds containing a styrylquinazoline motif, focusing on their antiproliferative and tyrosine kinase inhibitory properties. We initially characterized a series of p53 reactivator analogues, such as CP‐31398 [33]. Subsequently, we elucidated the mechanism of action of one derivative, IS20 (6b), demonstrating its capacity to induce oxidative stress by modulating *Ndrg1* gene expression and disrupting EGFR signaling [34]. Subsequent efforts explored thio‐analogs of styrylquinazoline, which increase bioavailability and improve binding to different conformations in the DFG pocket of ABL kinase [35], as well as sulfamoyl derivatives as dual IDH1/ABL inhibitors [36]. Moreover, several benzenesulfonates revealed pronounced anti‐GBM activity, driven by robust G2/M cell cycle arrest and enhanced tubulin polymerization [37, 38].

Given the limited efficacy of current kinase inhibitors in aggressive and resistant tumors, there is a clear need to develop novel compounds with enhanced properties. Leveraging our expertise, we have designed and synthesized a novel series of derivatives featuring a unique 7‐chloroquinazolinone core, a styryl moiety, and a tosyl group, a structural combination not previously reported. Among these, one compound has emerged as a promising IGF1R inhibitor, whose mechanism of action we have characterized in GBM cells, including under conditions of elevated glucose metabolism. Moreover, we have explored strategies for the effective combination of this approach with the EGFR inhibitor dacomitinib, emphasizing its potential for multi‐targeted therapeutic approaches. As monotherapies, EGFR inhibitors such as dacomitinib or osimertinib are effective in the NSCLC [39], but display limited activity in GBM [40]. Likewise, quinazoline‐based agents, such as lapatinib, have shown no efficacy in recurrent GBM [41]. In turn, afatinib demonstrated only modest activity in patients with recurrent GBM, and a similar outcome was observed for dacomitinib [40, 42].

## 2 Material and methods

### 2.1 Chemistry

All of the reagents were purchased from Sigma Aldrich. Microwave syntheses were performed in a CEM Discover 2.0 microwave reactor. Thin‐layer chromatography (TLC) experiments were performed on alumina‐backed silica gel 460 F_254_ plates (Merck, Darmstadt, Germany). The plates were illuminated under UV (254 nm and 360 nm). The melting points were determined on an Optimelt MPA‐100 (SRS, USA). All ^1^H and ^13^C NMR spectra were recorded on a Bruker Avance III 500 MHz FT‐NMR spectrometer (500 MHz for ^1^H and 101/126 MHz for ^13^C, Bruker Comp., Karlsruhe, Germany). Chemical shifts are reported in ppm (δ) using the signal of the solvent (DMSO‐*d*_*6*_) as the reference against the internal standard Si(CH_3_)_4_. Easily exchangeable signals were omitted when they were diffuse. Signals are designated as follows: s, singlet; d, doublet; dd, doublet of doublets; ddd, doublet of doublet of doublets; t, triplet; tt, triplet of triplets; td, triplet of doublets; m, multiplet. Mass spectra were measured using a maXis impact ESI/APCI‐Q‐TOF mass spectrometer (Bruker Daltonics, USA) with direct injection into an APCI source in the positive mode.

#### 2.1.1 General procedure for the preparation of tosyl aldehyde derivatives (A‐C)

A solution of hydroxybenzaldehyde (1.0 eq, 5 mmol), p‐toluenesulfonyl chloride (1.1 eq, 5.5 mmol), and triethylamine (2.5 eq, 12.5 mmol) in dichloromethane (CH_2_Cl_2_, 40 mL) was placed in a 100 mL round bottom flask. The mixture was stirred at room temperature for 3 h. The reaction mixture was washed with water (3 × 20 mL), and the water layer was extracted with EtOAc (3 × 20 mL). The organic layer was dried by MgSO_4_. After filtration and removal of the solvent under reduced pressure, the crude product was dried under air.

#### 2.1.2 General procedure for the preparation of 2‐methylquinazolin‐4(3H)‐one derivatives (W1‐2)

The 2‐aminobenzoic acid derivatives (23,4 mmol) were dissolved in acetic anhydride (110 mL), and the reaction mixture was stirred at 80 °C for 2 h. The solvent was then evaporated, and the crude product was washed with isopropanol and filtered out. To a dry solid (benzoxazin‐4‐one intermediates) was added 90 mL of ammonium hydroxide (32% NH_3_), and they were stirred at room temperature for 6 days. The product precipitated from the solution was filtered out and washed with water. The solid compound was dried under vacuum overnight.

#### 2.1.3 General procedure for the preparation of styrylquinazolinone derivatives (W1A‐C, W2B)

A mixture of the appropriate 2‐methylquinazolin‐4(3*H*)‐one derivative (W1‐2) (1 mmol) and the appropriate tosyl aldehyde (A‐C) (1 mmol) was heated in 3 mL of 80% concentrated acetic acid. The reaction was carried out in a microwave reactor at 130 °C for 1.5 h. The reaction mixture was then cooled in a refrigerator to precipitate the solid. The resulting solid was filtered under reduced pressure and recrystallized from acetic acid to afford the corresponding styrylquinazolinone derivative as a white solid.

The physicochemical data with spectroscopic analyses for all compounds are given in the Supplementary Information.

### 2.2 Molecular docking

The ligand molecule was drawn manually using Avogadro 1.1.1 software [43] and optimized within the UFF force field [44] (15 000 steps, steepest descent algorithm). The flexible, optimized ligand molecule was docked into the binding pocket of the five protein structures found in the PDB database: 1K3A (IGF1R), 1 M17 (EGFR), 2ZM3 (IGF1R), 3EKN (InsR) and 4HJO (EGFR). Vina software [45] was used for docking simulations, with all default procedures and algorithms implemented in AutoDock Vina during docking procedures. The docking procedure was carried out within the rectangular region of dimensions 22 × 22 × 16 Å^3^, which covers the originally co‐crystallized ligands present in the PDB structures as well as the closest amino acid residues that exhibit contact with those ligands. In addition to the flexibility of the ligand molecules, the rotation of selected sidechains (in particular: two Leu, one Lys, one Arg, one Val, two Met and two Asp) in the proximity of the co‐crystalized ligands was allowed. Visual inspection of the location and orientation of the docked ligands was accompanied by RMSD‐based clustering in order to control the uniformity of the binding pattern. Before docking the compound, the adopted methodology was validated by performing docking for each of the ligands present in the crystal structure of the given protein. All procedures were the same as described above.

### 2.3 Measurements of tyrosine kinase activity

RTK inhibition was measured using Kinase Selectivity Profiling Systems (TK‐1 and TK‐2) and ADP‐Glo Kinase Assay (Promega). W1B was dissolved in 100 μm DMSO and a 1 μm solution was prepared in 1× Kinase Reaction Buffer (40 mm Tris—pH 7.5; 20 mm MgCl_2_; 0.1 mg·mL^−1^ BSA; 50 μm DTT) with a final DMSO concentration of 5%. Then, each kinase (EGFR, HER2, HER4, IGF1R, InsR, KDR, PDGFRa, PDGFRb) or (ABL1, BRK, BTK, CSK, Fyn A, Lck, Lyn B, Src) from an 8‐well strip was dissolved in 95 μL of 2.5× Kinase Reaction Buffer, and the 8‐well strips containing substrates in 15 μL of 100 μm ATP. The tyrosine kinase inhibition assay was performed on a white 384‐well plate by transferring 1 μL of W1B solutions, then adding 2 μL of the previously prepared kinase and substrate solutions, then incubating for 1 h at room temperature. Kinase activity was detected by adding 5 μL of ADP‐Glo™ Reagent to each well, shaking the plate for two minutes, and incubating for 40 min at room temperature. Then, 10 μL of Kinase Detection Reagent was added, remixed, and incubated for 30 min at room temperature. Luminescence was measured using a Varioskan LUX multidetector plate reader (Thermo Fisher Scientific, USA), with analysis and graphical representation employing graphpad prism 9 (GraphPad Software, USA).

### 2.4 Binding assays (microscale thermophoresis – MST)

W1B affinity to the IGF1R protein was measured by MST using the Monolith NT.115 Blue/Red device (NanoTemper Technologies, Germany). Experiments were performed in a buffer containing 50 mm HEPES (pH = 7.5), 300 mm NaCl, 10 mm MgCl_2_ and 0.05% Tween 20. Recombinant human IGF‐I R/IGF1R protein (His‐tagged) (Bio‐Techne) was labeled using the Monolith His‐Tag RED‐tris‐NTA 2nd Generation labeling kit (NanoTemper Technologies). The final labeled protein concentration was 50 nm. Final serial dilutions of W1B were prepared starting at 10 μm, with 2‐fold dilutions to 0.000305 μm in assay buffer. For Kd determination, labeled proteins were incubated with serial dilutions of W1B for 15 min at room temperature. Samples were then centrifuged (12 000 × ***g*** for 10 min), placed in standard capillaries (NanoTemper Technologies) and measured at 25 °C using 80% LED power and 40% MST power. Experiments were repeated at least three times. Data analysis was performed using mo.affinity analysis 2.3 software (NanoTemper Technologies). All final graphs were prepared using graphpad prism 9.

### 2.5 Cell culture

The human GBM cell lines U87MG (RRID:CVCL_0022), T98G (RRID:CVCL_0556), LN18 (RRID:CVCL_0392) and LN229 (RRID:CVCL_0393) were purchased from ATCC. The human breast carcinoma cell line MCF‐7 (RRID:CVCL_0031), and the lung adenocarcinoma PC‐9 (RRID:CVCL_B260) were obtained from Merck. Normal human dermal fibroblasts (NHDF) were purchased from PromoCell. The U‐251 (RRID:CVCL_0021) GBM cell line was kindly provided by Prof. G. Kramer‐Marek from the Institute of Cancer Research in London, United Kingdom. GBM cell lines and MCF‐7 cells were cultured in Dulbecco's modified Eagle's medium F‐12 (DMEM), whereas PC‐9 cells were cultured in RPMI 1640 medium. Both media were supplemented with 10% heat‐inactivated fetal bovine serum—FBS (all reagents from Merck). For some experiments, LN229 cells (later named LN229 HG) were also cultured in DMEM containing high glucose (DMEM HG; Merck) and supplemented with 10% heat‐inactivated FBS. The NHDF cell line was cultured in fibroblast growth medium with a low‐serum content (PromoCell). Each complete medium contained penicillin and streptomycin (1% v/v; Gibco). Cell lines were cultured at 37 °C with a 5% CO_2_ humidified atmosphere. Cell lines were routinely tested for *mycoplasma* contamination using the PCR technique. All cell lines used in this study were purchased specifically for the purposes of this research and have been authenticated within the past three years.

### 2.6 Cytotoxicity

Cells were seeded in 96‐well plates (Nunc) at a density of 5000 cells per well (U‐251, U87MG, T98G, LN18, LN229, LN229 HG, MCF‐7, PC‐9) or 4000 cells per well (NHDF) and incubated under standard conditions at 37 °C for 24 h. The assay was conducted after a 72 h incubation period with the various concentrations of the tested compounds. Then, DMEM without phenol red with CellTiter 96®AQueous One Solution‐MTS (Promega) solution was added to each well and incubated for 1 h at 37 °C. The optical densities of the samples were measured at 490 nm using a multi‐plate reader (Varioskan LUX). The obtained results were compared to the control and were calculated as inhibitory concentration (IC_50_) values with graphpad prism 9. All experiments were performed independently four times, with each compound tested in triplicate.

### 2.7 Immunoblotting

The LN229 and LN229 HG cells were seeded in a 3 cm Petri dishes (Nunc) at a density of 500 000 and incubated for 24 h at 37 °C. After 24 h, DMEM or DMEM HG medium was removed and various concentrations of W1B compound were added. For the combination therapy experiments, LN229 HG cells were treated with W1B, dacomitinib, or their combination at the IC_50_ dose. The cells were then incubated for an additional 24 h and collected, centrifuged, and lysed on ice in complete RIPA buffer containing Halt Protease Inhibitor Cocktail, Halt Phosphatase Inhibitor Cocktail, and 0.5 M EDTA (all Thermo Scientific). The protein amount was determined using a BCA Protein Assay Kit (Thermo Scientific) according to the manufacturer's protocol. Equal amounts of the proteins were electrophoresed on SDS/PAGE gels and transferred onto nitrocellulose membranes. After blocking with 5% non‐fat milk with TTBS, the membranes were cut and incubated overnight at 4°C with one of the chosen primary antibodies (from CellSignaling or Abcam): EGFR (D38B1) (#4267), IGF‐I Receptor β (D23H3) (#9750), Akt (#9272), phospho‐Akt (Ser473) (D9E) (#4060), p70 S6 Kinase (49D7) (#2708), phospho‐S6 Ribosomal Protein (Ser240/244) (D68F8) (#5364), RAS (EP1125Y) (#ab52939), vinculin (E1E9V) (#13901), GAPDH (14C10) (#2118), cyclophilin β (D1V5J) (#43603), and cofillin (D3F9) (#5175). The antibodies were diluted in 5% non‐fat milk with TTBS. The membranes were then washed and incubated with horseradish peroxidase‐conjugated secondary antibodies for 1 h at room temperature. The chemiluminescence signals were recorded after staining with a SuperSignal™ West Atto Chemiluminescent Substrate (Thermo Scientific) using a ChemiDoc™ XRS+ System (BioRad). The experiments were performed six times. The densitometric analysis was performed using ImageJ 1.41. Statistical analysis (one‐way ANOVA with Dunnett's post hoc test) and graph preparation used graphpad prism 9.

### 2.8 Combination therapy with EGFR inhibitor

The LN229 or LN229 HG cells were seeded in 96‐well plates (Nunc) at a density of 5000 cells per well and incubated at 37 °C for 24 h. After 24 h, solutions of W1B, dacomitinib, or their combination at IC_50_ were added and incubated for 72 h. Then, DMEM without phenol red with CellTiter 96®AQueous One Solution‐MTS (Promega) solution was added to each well and incubated for 1 h at 37 °C. The optical densities of the samples were measured at 490 nm using a multi‐plate reader (Varioskan LUX). Each variation was tested in triplicate in a single experiment with each experiment being performed at least three or four times. Drug combination effects were calculated using CompuSyn software [46]. The CI values are presented in Table S3. Statistical analysis (two‐way ANOVA with Tukey's post hoc test) and graph preparation were performed using graphpad prism 9.

### 2.9 Computational and biomimetic studies

The ACD/Percepta software (version 2012, Advanced Chemistry Development, Inc., Toronto, ON, Canada) was used in the *in silico* studies. Statistical analysis of the results obtained was made using the Minitab 18 Statistical Software (Minitab Inc., State College, USA).

The Shimadzu Vp liquid chromatographic system (Shimadzu, Kyoto, Japan) equipped with LC 10AT pump, SPD 10A UV–Vis detector, SCL 10A system controller, CTO‐10 AS chromatographic oven and Rheodyne injector valve with a 20 μL loop was applied in the HPLC‐IAM measurements. The solution of W1B was prepared in methanol (Merck, Darmstadt, Germany) at a concentration of 1 mg·mL^−1^. The optimization process of the chromatographic separation was made before the experiment. The flow rate of the mobile phases was established to 1 mL·min^−1^ and the temperature was set at 20 °C. The tested compound was detected with the UV light at 340 nm. The IAM.PC.DD2 stationary phase (100 × 4.6 mm i.d., 10 μm; Regis Chemicals Company, Morton Grove, USA) was used as the stationary phase while the buffered solutions of acetonitrile were used as mobile phases. The mobile phases composition was in each measurement: 0.4; 0.5; 0.6; 0.7% v/v acetonitrile‐buffer (pH = 7.4). The buffer was prepared from the solutions of both Na_2_HPO_4_ (0.02 mol·dm^−3^) and citric acid (0.01 mol·dm^−3^). The dead time values were measured from the citric acid peaks. All the reported logarithms of the retention factor were measured three times. The values of peak asymmetry factor were in the acceptable range.

### 2.10 *In vivo* studies

All experiments using the *Danio rerio* model were performed in accordance with European regulations, in particular the Directive 2010/63/EU, and the corresponding Polish legislation implementing this directive (Act of 15 January 2015 on the protection of animals used for scientific or educational purposes). According to these regulations, Danio rerio larvae up to 120 h post‐fertilization (hpf) are not considered protected animals. Therefore, no animal experimentation license or ethical approval number was required for the procedures described in this study. All experiments using the *in vivo* model were carried out respecting the 3Rs principle and other guidelines (e.g., ARRIVE) for working with animals.

Strain: Wild‐type AB strain. Developmental stage: Embryos (Toxicity assays were performed from 0 to 96 hpf. Microinjection experiments were conducted between 48 and 120 hpf). Sex: Sex cannot be determined at the embryonic and larval stages used in this study. In zebrafish, phenotypic sex differentiation occurs later during juvenile development (typically several weeks post‐fertilization). Embryos and larvae were maintained under standard conditions at 26 °C in aerated water with oxygen saturation maintained at ≥ 80%. Embryos were kept in E3 embryo medium containing 5 mm NaCl, 0.17 mm KCl, 0.33 mm CaCl_2_, and 0.33 mm MgSO_4_.

#### 2.10.1 Zebrafish toxicity studies

Toxicity testing was carried out following a modified OECD Guideline 236 protocol. In brief, freshly collected zebrafish (Danio rerio) embryos were placed into 24‐well plates, five embryos per well, with two wells assigned to each treatment group. Embryos were maintained in E3 medium and exposed to W1B, lapatinib, dacomitinib, or osimertinib at concentrations ranging from 0 to 50 μm, each compound tested on a separate plate. Incubation was performed under controlled conditions at 28 ± 0.5 °C with a light–dark cycle of 14/10 h. After 96 h of continuous exposure, the following developmental endpoints were evaluated: (1) embryo coagulation, (2) lack of somite formation, (3) failure of tail detachment, and (4) absence of heartbeat. The half‐maximal inhibitory concentration (IC_50_) values were determined from dose–response curves generated for each compound, providing a quantitative measure of toxicity.

#### 2.10.2 Zebrafish xenograft experiments

The LN229 cells were cultured until reaching approximately 70% confluence, washed with phosphate‐buffered saline (PBS), and detached for labeling. Cells were stained with Vybrant CM‐DiI (Thermo Fisher; 4 μL·mL^−1^ in 1× PBS) for 10 min at 37 °C in the dark. After staining, the cell suspension was adjusted in PBS to a final concentration of 2.5 × 10^8^ cells·mL^−1^. Approximately 500–1000 fluorescently labeled cells were microinjected into the yolk sac of 2 days post‐fertilization (dpf) zebrafish embryos. Successful engraftment was verified by fluorescence imaging (Zeiss Discovery V8), after which embryos were randomly assigned to treatment groups: vehicle control (E3 medium), W1B, lapatinib, osimertinib, dacomitinib, each tested at concentrations of 5, 7.5, and 10 μm. In addition, the combination of W1B and dacomitinib at a dose of 7.5 μm was tested. Following treatment, embryos were incubated at 31 °C for 72 h, after which tumor size was evaluated. Fluorescence images were acquired using a Zeiss Discovery V8 microscope, and quantitative analysis was performed with FIJI/ImageJ software. Tumor‐derived fluorescence was measured at baseline (immediately post‐injection) and at 72 h and changes in tumor size were expressed as a percentage relative to the untreated E3 control group, which was set at 100%.

## 3 Results

### 3.1 Synthesis of novel compounds

**Fig. 2:**
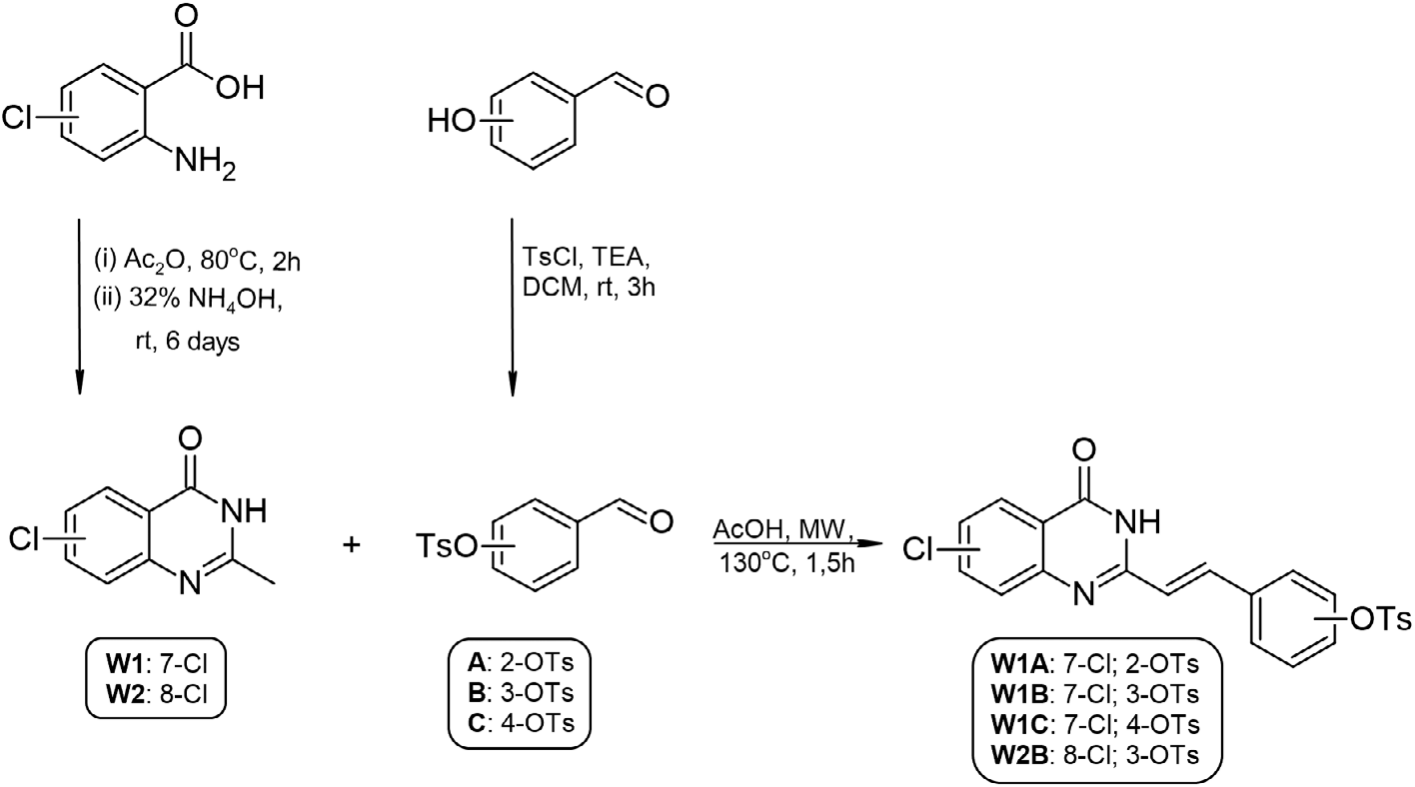
Synthesis of the studied compounds.

The synthesis of styrylquinazolinones was performed in a multistep process as shown in Fig. 2. The first step involved the preparation of the starting quinazolinone cores, with 2‐methyl‐4(3*H*)quinazolinones (W1‐2) synthesized via the cyclocondensation of anthranilic acid with acetic anhydride, followed by treatment with concentrated aqueous ammonia [47, 48]. This method enabled efficient formation of the quinazolinone core substituted with a methyl group at the C‐2 position. In parallel, tosylbenzaldehyde derivatives (A‐C) were obtained through a nucleophilic substitution reaction between *p*‐toluenesulfonyl chloride and various hydroxybenzaldehydes. The reaction was carried out in dichloromethane using triethylamine as a base [49, 50]. The key step in the synthetic pathway involved the condensation of the obtained 2‐methyl‐4(3*H*)quinazolinone derivatives (W1‐2) with the corresponding tosylbenzaldehyde derivatives (A‐C). This reaction was performed under microwave irradiation at 130 °C for 90 min in glacial acetic acid [37]. The application of microwave‐assisted heating enabled a substantial reduction in reaction time compared to conventional thermal methods [33]. All synthesized styrylquinazolinones (W1A‐C and W2B) were purified by recrystallization in concentrated acetic acid. Their structures were confirmed by spectroscopic analyses, including ^1^H and ^13^C nuclear magnetic resonance (NMR) spectroscopy, as well as high‐resolution mass spectrometry (HRMS). These data are provided in the Supplementary Information (Fig. S1‐S8).

### 3.2 Antiproliferative activity of styrylquinazolinones toward glioblastoma

The synthesized compounds were assessed for antiproliferative activity against GBM cells. For this purpose, five human GBM cell lines with different genetic and proteomic profiles were selected. As summarized in Table 1, only one of the tested derivatives (W1B) containing a 7‐chloroquinazoline ring with a tosyl group substituted at position 3 in the styryl moiety showed very good biological activity. In contrast, two other 7‐chloroquinazoline analogues were inactive, with the concentration required to inhibit 50% of cell proliferation (IC_50_) exceeding 25 μm. The W2B derivative, containing an 8‐chloroquinazoline scaffold, displayed poor solubility. Among the tested models, the LN229 cell line was the most susceptible, with W1B achieving an IC_50_ value of 3.57 μm. Notably, this derivative also exhibited strong activity against U‐251 and U87MG cells, with IC_50_ values of 5.43 and 5.88 μm, respectively. In contrast, LN18 cells were more resistant to W1B (11.06 μm), whereas T98G cells exhibited markedly reduced susceptibility, with an IC_50_ of 21.01 μm. Evaluation of two additional epithelial cell lines (Table S1) revealed that compound W1B exhibited moderate antiproliferative activity against MCF‐7 cells. In contrast, its activity against PC‐9 cells (IC_50_ = 6.54 μm) was comparable to that observed in U‐251 and U87MG cells.

**Table 1:** Antiproliferative activity of the tested compounds against a panel of glioblastoma human cell lines and normal human dermal fibroblasts (NHDF), expressed as IC_50_ values (concentration required to inhibit 50% of cell proliferation) ± SD.

Compounds	Antiproliferative activity–IC_50_ [μm]					
	U‐251	U87MG	T98G	LN18	LN229	NHDF
W1A	> 25	> 25	> 25	> 25	> 25	‐
W1B	5.43 ± 0.97	5.88 ± 1.20	21.01 ± 2.87	11.06 ± 1.50	3.57 ± 1.03	9.88 ± 1.18
W1C	> 25	> 25	> 25	> 25	> 25	‐
W2B	> 20	> 15	> 25	> 20	> 20	‐
Osimertinib	20.13 ± 0.34	11.24 ± 0.46	7.31 ± 2.19	7.35 ± 0.27	8.99 ± 0.37	6.27 ± 0.38
Lapatinib	11.37 ± 0.46	21.22 ± 1.37	17.87 ± 0.42	12.74 ± 1.29	12.60 ± 0.32	‐
Dacomitinib	3.80 ± 0.61	3.66 ± 0.39	11.98 ± 0.41	5.56 ± 0.27	5.84 ± 0.17	‐

In our assays, we also tested two FDA‐approved reference drugs, including osimertinib, a third‐generation EGFR inhibitor that crosses the BBB, and two quinazoline‐based HER2/EGFR inhibitors, such as lapatinib and dacomitinib. In general, osimertinib exhibited a weaker activity profile than W1B, with two exceptions. In LN18 cells, its activity was comparable, whereas the EGFR inhibitor showed higher potency in T98G cells. In contrast, lapatinib consistently displayed lower activity across all tested cell lines. The third reference compound was dacomitinib, which exhibited a similar activity profile to W1B.

We further evaluated the selectivity of W1B against normal fibroblasts and compared it with osimertinib. The therapeutic index (TI) was calculated as the ratio of IC_50_ for normal cells to IC_50_ for cancer cells, with higher values indicating greater safety for normal tissues. W1B exhibited TI values of 2.8, 1.8, and 1.7 against LN229, U‐251, and U87MG cells, respectively. In contrast, osimertinib showed limited selectivity (TIs below 0.8), consistent with the profile of many clinically approved anticancer agents.

### 3.3 Inhibitory profile of W1B on receptor tyrosine kinases

**Fig. 3:**
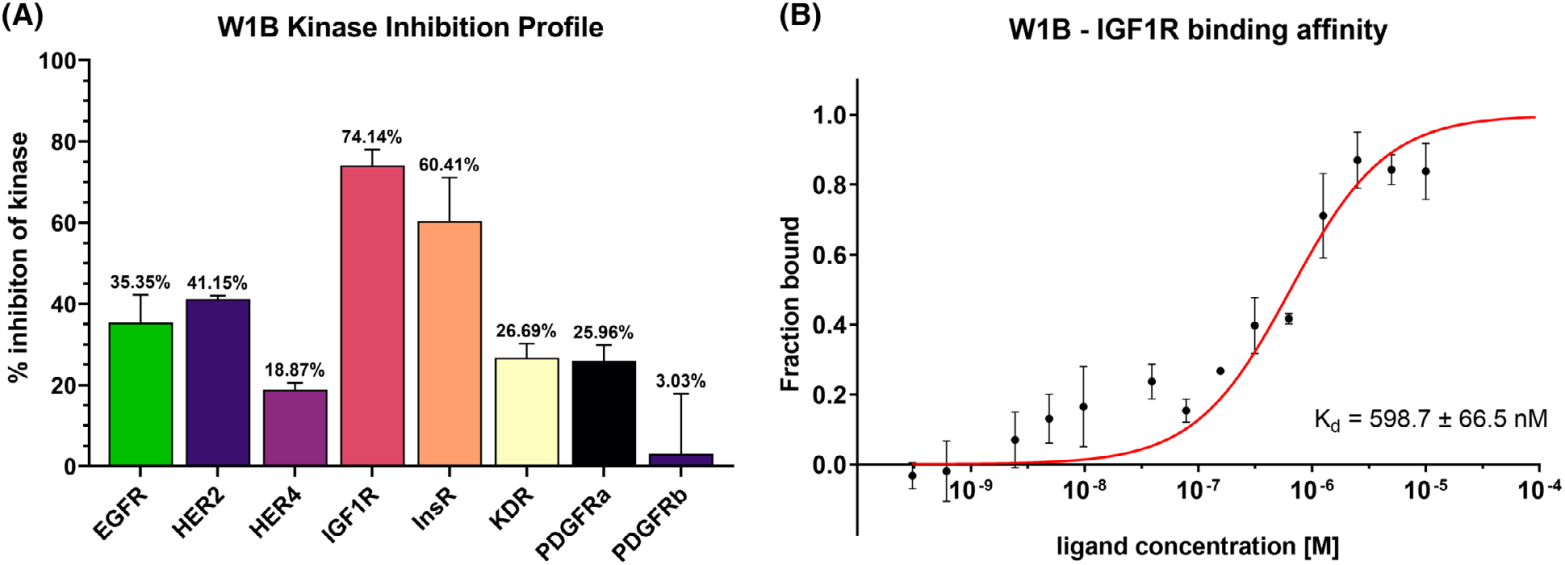
Inhibition levels on the receptor tyrosine kinases by the W1B compound. A graphical representation of W1B inhibition is shown as mean [%] and standard deviation (SD) (*n* = 4) (A). The binding affinity of W1B to IGF1R was measured using the microscale thermophoresis (MST) method to determine the dissociation constant (*K*_d_). A fraction bound line chart was plotted from the showcased mean points and their SD (*n* = 3) (B).

We screened our lead compound—W1B at a concentration of 1 μM, for inhibition of a panel of RTKs using Kinase Profiling System TK‐1. This ATP‐competitive profiling assay included eight representative RTKs: EGFR, HER2, HER4 (from ERBB family), as well as IGF1R, InsR, KDR, and two PDGFR isoforms. As depicted in Fig. 3A, W1B exhibited pronounced inhibitory activity against two insulin‐related RTKs. The highest effect was observed for IGF1R, where kinase activity was diminished by more than 74%. In the case of InsR, a more than 60% reduction of kinase activity was observed. W1B also exhibited moderate inhibitory effects on two members of the ERBB family, reducing EGFR and HER2 kinase activities by 35% and 41%, respectively. Moreover, the tested compound showed rather weak inhibitory potential against HER4, KDR, and PDGFRs. We also assessed W1B with regard to inhibition of non‐receptor tyrosine kinases, such as ABL, BRK, BTK, CSK, Fyn, Lck, Lyn, and Src. The results are presented in Table S2. Notably, our lead compound showed selective activity against RTKs. Indeed, W1B did not reduce the activity of any non‐receptor kinase by more than 20%.

In further experiments, we focused on determining the binding affinity of W1B with IGF1R. For this purpose, a microscale thermophoresis (MST) technique was used, and the IGF1R His‐Tag protein was labeled with a fluorescent tag. The binding curve showing the bound and unbound states of the ligand with the protein is presented in Fig. 3B. From these traces, the dissociation constant (*K*_d_) for W1B was determined to be 598 nm.

### 3.4 Binding interactions of W1B with IGF1R and EGFR kinases

The next step involved molecular docking studies to further characterize W1B interactions with target kinases. Five protein structures were selected from the Protein Data Bank (PDB): 1K3A (IGF1R), 1M17 (EGFR), 2ZM3 (IGF1R), 3EKN (InsR), and 4HJO (EGFR), into whose binding pockets the ligand was docked. First, the docking protocol was verified by comparing the experimentally determined ligand pose (based on XRD data) with the most energetically favorable pose obtained by docking, as shown in Fig. S9. In all cases, it was possible to reconstruct the ligand position in the binding pocket with high accuracy, as well as its conformation (which is significant given the presence of many rotatable bonds in the W1B). The largest, yet still acceptable, deviations were observed for flexible side chains of erlotinib in PDB structures 1M17 and 4HJO.

The binding energies corresponding to the most energetically favorable W1B–protein arrangements were then determined for: 1K3A (−8.1 kcal·mol^−1^), 1M17 (−9.4 kcal·mol^−1^), 2ZM3 (−8.6 kcal·mol^−1^), 3EKN (−9.2 kcal·mol^−1^), and 4HJO (−9.9 kcal·mol^−1^). It is worth noting some differences in the binding energies involving the two alternative structures of the same proteins, namely IGF1R and EGFR, which amounted to 0.6 kcal·mol^−1^ and 0.5 kcal·mol^−1^, respectively.

**Fig. 4:**
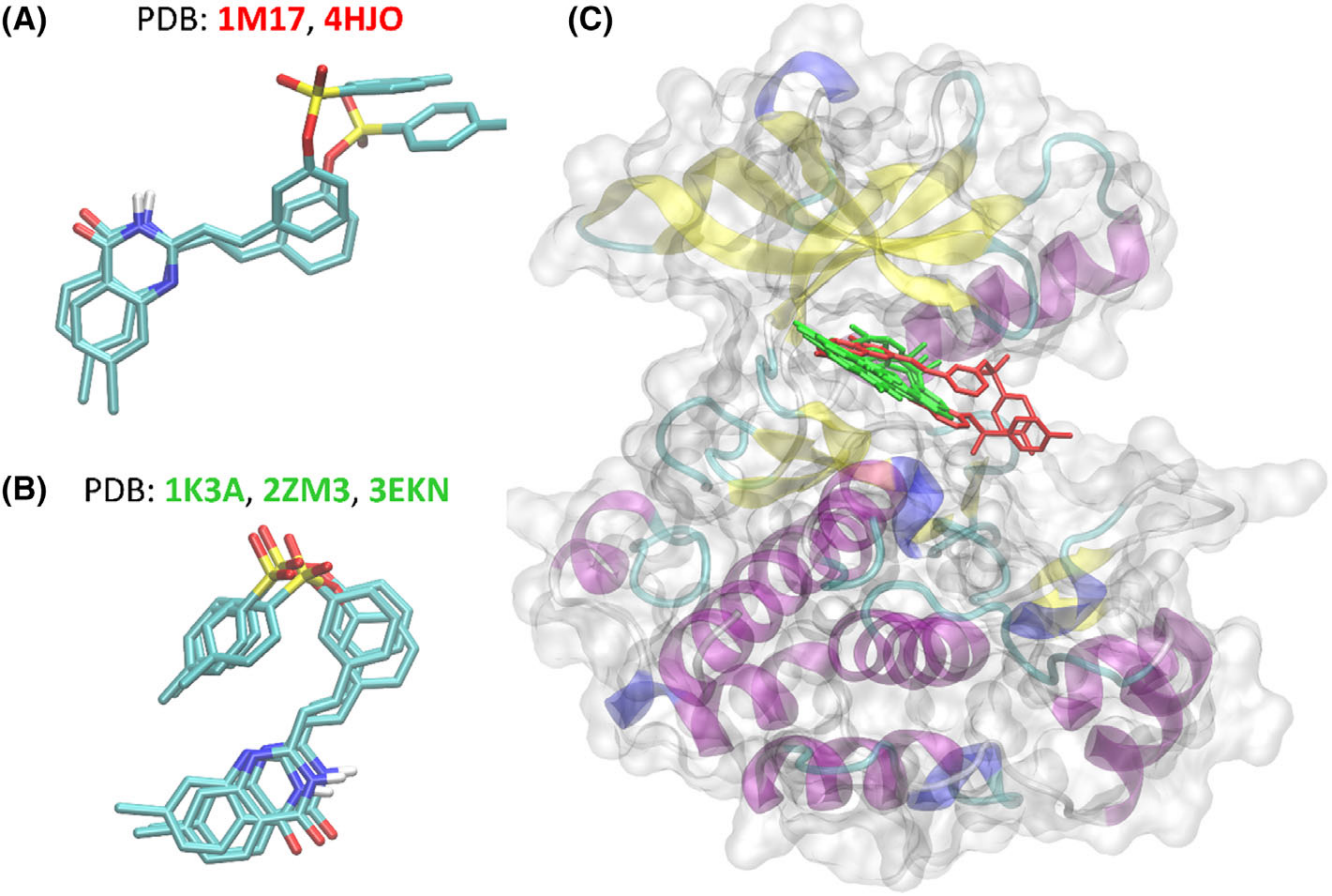
Superposition of energetically favorable W1B arrangements docked to EGFR (PDB structures: 1M17 and 4HJO) (A) and to IGF1R/InsR (PDB: 1K3A, 2ZM3, and 3EKN) (B). Panel (C) provides a schematic representation of the possible ligand orientations shown in (A) and (B) relative to the protein structure of PDB: 1M17. Ligand colors correspond to the color coding of the respective PDB structures in panels (A) and (B).

The next stage involved structural analysis of the W1B‐protein complexes corresponding to the most favorable binding energies. Notably, all proteins are structurally similar, with an average RMSD per residue after superposition ranging from approximately 0.9 to 1.5 nm, depending on the pair compared. Therefore, a relatively similar ligand–protein interaction mechanism can be expected. As shown in Fig. 4A,B, W1B can adopt two distinct poses within the binding pocket, depending on the target protein, which are accompanied by different ligand conformations. In complexes with EGFR (PDB: 1M17 and 4HJO), W1B occupies the binding pocket in a more extended pose, associated with an elongated conformation of the molecule. In contrast, when bound to IGF1R and InsR (PDB: 1K3A, 2ZM3, and 3EKN), W1B adopts an alternative pose, characterized by a partially folded conformation driven in part by the rotation of an S–O bond. Regardless of these differences, the ligand occupies a similar binding pocket region (Fig. 4C), with slightly larger contact areas for the extended conformation, correlating with higher binding energies. Furthermore, significant conformational variability and different orientations of ligands with similar structural characteristics are typical for ligands present in the crystallized structures of the studied proteins.

**Fig. 5:**
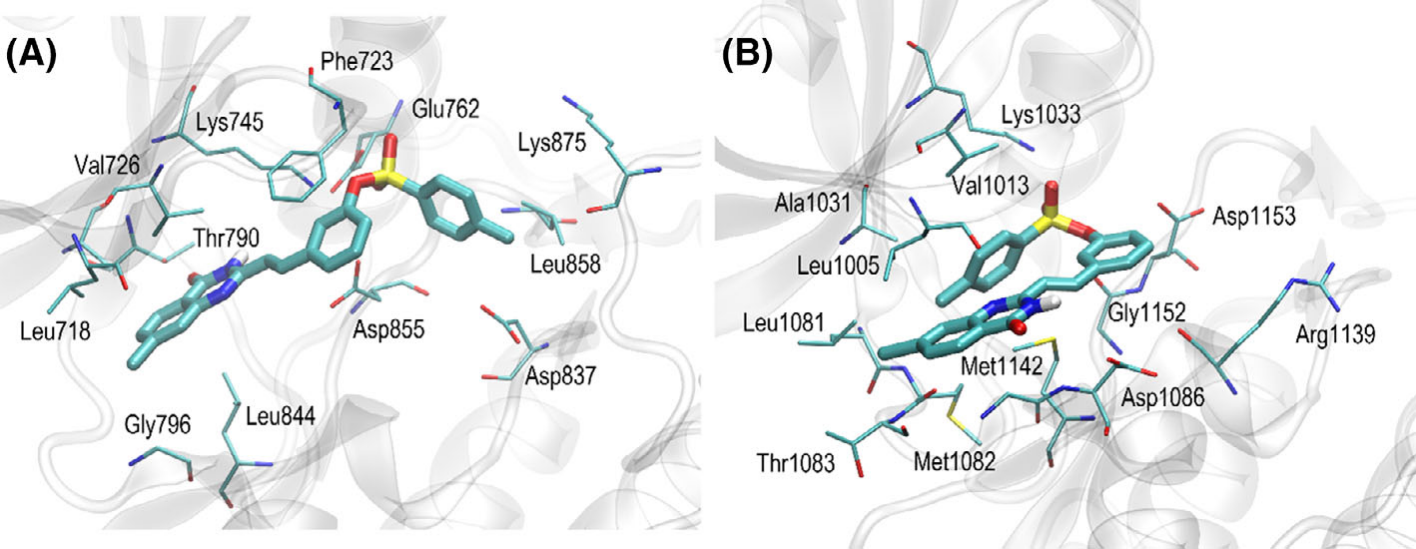
The energetically favorable location of the ligand molecule bound to the EGFR (PDB: 1M17) (A) and to the IGF1R (PDB: 2ZM3) (B). The ligand molecule is shown as thick sticks whereas all the closest amino acid residues (< 0.4 nm) are represented by thin sticks.

Differences in ligand–protein interactions characteristic of the identified ligand orientations are illustrated in Fig. 5A,B, using PDB structures 1M17 (EGFR) and 2ZM3 as representative examples. First, we focused on the causes of different ligand orientations in proteins with similar structures. In addition to differences in the backbone conformation, which are conformationally‐locked during docking, a potential cause could be differences in the primary sequence. The binding pocket fragment near the bound ligand differs in the type of amino acid residues present. For instance, Met1079 and Met1142 (present in IGF1R/InsR: 1K3A, 2ZM3, and 3EKN) are replaced by Leu and Thr in EGFR (1M17 and 4HJO). Additional substitutions include Gly1152 → Thr, Asp1086 → Cys, Glu1080 → Gln, Val1063 → Cys, Thr1083 → Pro, Met1165 → Leu, and Gln1007 → Ser. These changes, which affect the interaction characteristics between individual amino acid residues and the W1B, qualitatively explain the structural differences in the ligand–protein complex. In particular, the presence of Leu858 in EGFR (1M17 and 4HJO) enables CH–π interactions with the aromatic–aliphatic portion of W1B, thereby stabilizing its extended conformation. In contrast, the absence of this interaction in the other structures favors a folded conformation of the ligand. Additionally, in the context of changes in the backbone conformation, the most significant structural difference in the proteins appears to be the deviation of the loop connecting the β‐strands around Phe1010 (the equivalent in PDB: 1M17 is Phe723). This last change allows energetically favorable *π*‐*π* interactions with the phenyl rings of W1B in the case of EGFR (PDB: 1M17 and 4HJO). In the other structures, the analogous residues are shifted away from the binding pocket and do not contact the ligand, while the space left unoccupied by Phe is instead filled by a fragment of W1B in its folded conformation.

When considering the residues involved in the driving force for binding, it is particularly worth mentioning the nonpolar residues (Leu, Val, Ala) and weakly polar residues (Met), whose cluster forms the main center of favorable interactions, primarily CH‐*π* interactions, with the quinazoline ring of W1B. These favorable interactions in this region are further enhanced by contacts with the sidechains of Thr, which can be either polar or nonpolar, depending on the fragments of the sidechain and the ligand. In the case of the folded conformation of W1B in the IGF1R/InsR (PDB: 1K3A, 2ZM3, and 3EKN), interactions with the aforementioned nonpolar and weakly polar residues also involve the outer phenyl ring and its substituent. Here, the primary type of responsible interaction is also CH‐*π*. Conversely, in the extended conformation of W1B in EGFR (PDB: 1M17, 4HJO), there is an additional *π*–*π* contact with the sidechain of Phe. These interactions are complemented by a series of polar interactions, mainly involving Asp residues and the flexible side chain of Lys, which acts as a donor for hydrogen bonds with the sulfonyl group.

### 3.5 W1B‐mediated inhibition of IGF1R and EGFR signaling pathways

**Fig. 6:**
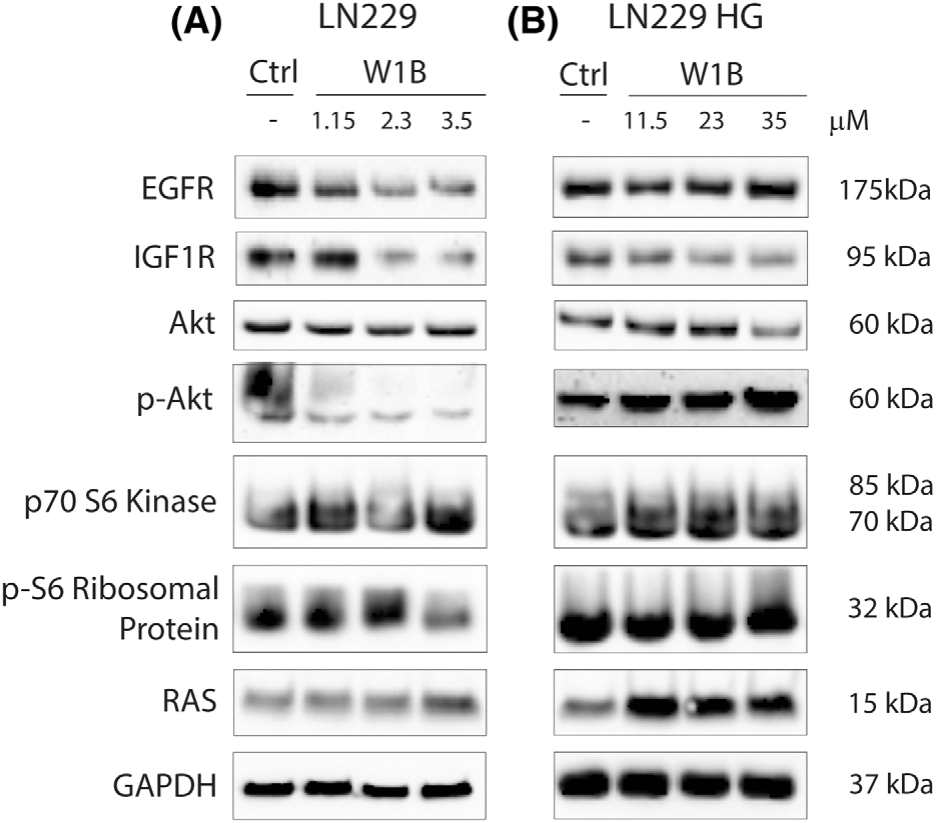
Protein expression profiles in LN229 (A) and LN229 HG (B) cells following incubation with W1B at various concentrations. The immunoblots shown are representative of six independent experiments. Proteins were cropped, and full‐length blots are provided in Fig. S12.

To further validate our previous findings, we assessed the cellular effects of W1B on IGF1R‐ and EGFR‐mediated signaling pathways. In particular, we evaluated the protein levels of downstream signaling elements of these pathways, including Akt, p‐Akt, Ras, as well as two mTOR‐regulated proteins: p70 S6 kinase and p‐S6 ribosomal protein. Representative western blot results are shown in Fig. 6A, with the corresponding densitometric analyses provided in Fig. S11. Uncropped blot images are provided in Fig. S12. The analysis revealed that W1B significantly reduced EGFR and IGF1R levels in LN229 cells. The most significant downregulation was observed at a concentration of 3.5 μm and 2.3 μm, at which the levels of both proteins decreased by more than 2‐fold. As expected, this resulted in the inhibition of one of the key effectors of the signaling pathway cascade, namely Akt activation. We observed that both W1B concentrations reduced the level of Akt phosphorylation at Ser473. To further confirm inhibition of Akt signaling, the ratio of phosphorylated Akt to total Akt was analyzed and showed a comparable level of suppression (see Fig. S11).

Next, we examined two downstream effectors of mTOR signaling, which displayed an interesting expression pattern in response to treatment. At 2.3 μm, W1B reduced p70 S6K levels but enhanced p‐S6 ribosomal protein expression. Conversely, treatment at 3.5 μm led to an almost 1.9‐fold increase in p70 S6K and a 1.6‐fold reduction in p‐S6 levels compared to untreated cells. Treatment with W1B had minimal effect on RAS levels.

Furthermore, we investigated the effect of the small‐molecule inhibitor W1B on LN229 cells under conditions of elevated glucose availability. For this purpose, LN229 cells were cultured in high‐glucose Dulbecco's modified Eagle's medium (DMEM) (hereafter referred to as LN229 HG). Under these conditions, the newly determined IC_50_ value was 34.4 ± 3.5 μm, indicating that enhanced metabolic activity diminishes the potency of W1B to some extent. Consequently, we assessed the impact of W1B on the EGFR/IGF1R signaling axis in LN229 HG cells. As depicted in Fig. 6B, EGFR expression remained at high levels after treatment with W1B. In contrast, IGF1R levels were significantly reduced (almost 1.5‐fold) after treatment with 23 μm and 35 μm of W1B. Despite the inhibition of IGF1R, the Akt/mTOR signaling pathway was upregulated in LN229 HG cells, as evidenced by increased levels of p‐Akt and p70 S6K. Additionally, RAS abundance was also elevated under these high‐glucose conditions.

### 3.6 Combination strategy of W1B with EGFR inhibitor

**Fig. 7:**
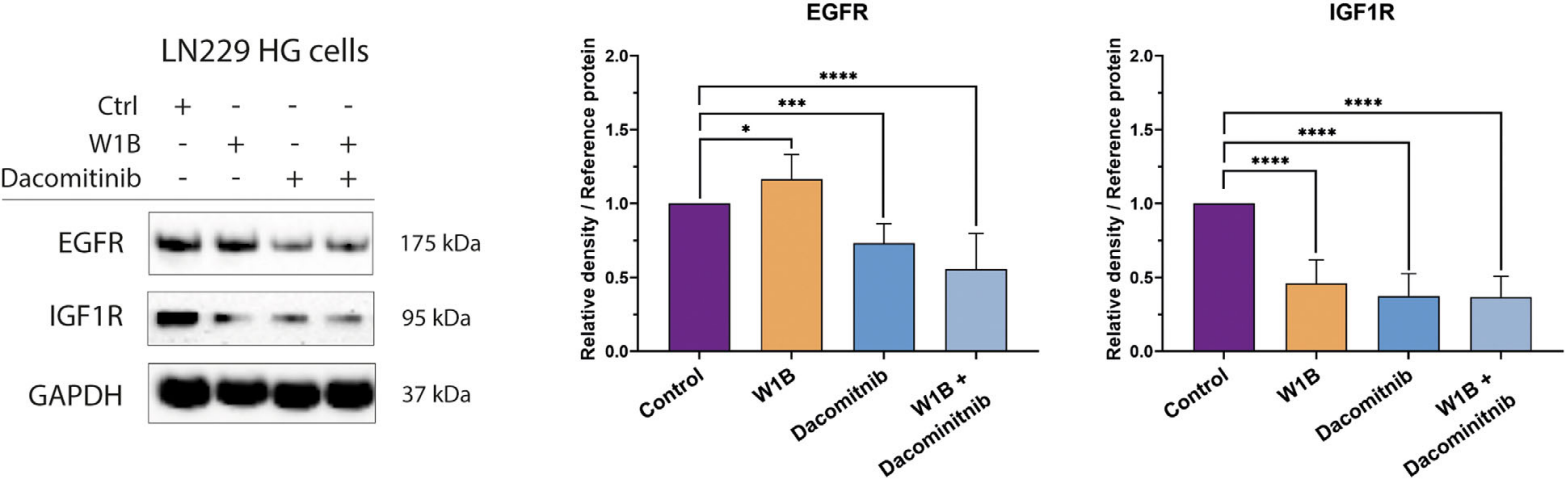
Protein expression in LN229 HG cells after 24 h treatment with W1B (35 μm), dacomitinib (5.4 μm), or their combination. Relative protein levels were normalized to the reference protein and compared to control (untreated) cells. The immunoblots shown are representative of six independent experiments. Proteins were cropped, and full‐length blots are provided in Fig. S13. Data are presented as mean ± SD (*n* = 6) and were analyzed using one‐way ANOVA with Dunnett's post hoc test (*P < 0.05, ***P < 0.001, ****P < 0.0001).

We evaluated the effects of a combination therapy regimen comprising W1B and dacomitinib on the IGF1R and EGFR kinases under conditions of elevated glucose concentration. As presented in Fig. 7, simultaneous administration of the compounds resulted in a comparable degree of IGF1R inhibition to that observed with W1B. However, a marked decrease in EGFR activity was observed when both inhibitors were administered simultaneously, compared to monotherapy. Uncropped blot images are provided in Fig. S13.

**Fig. 8:**
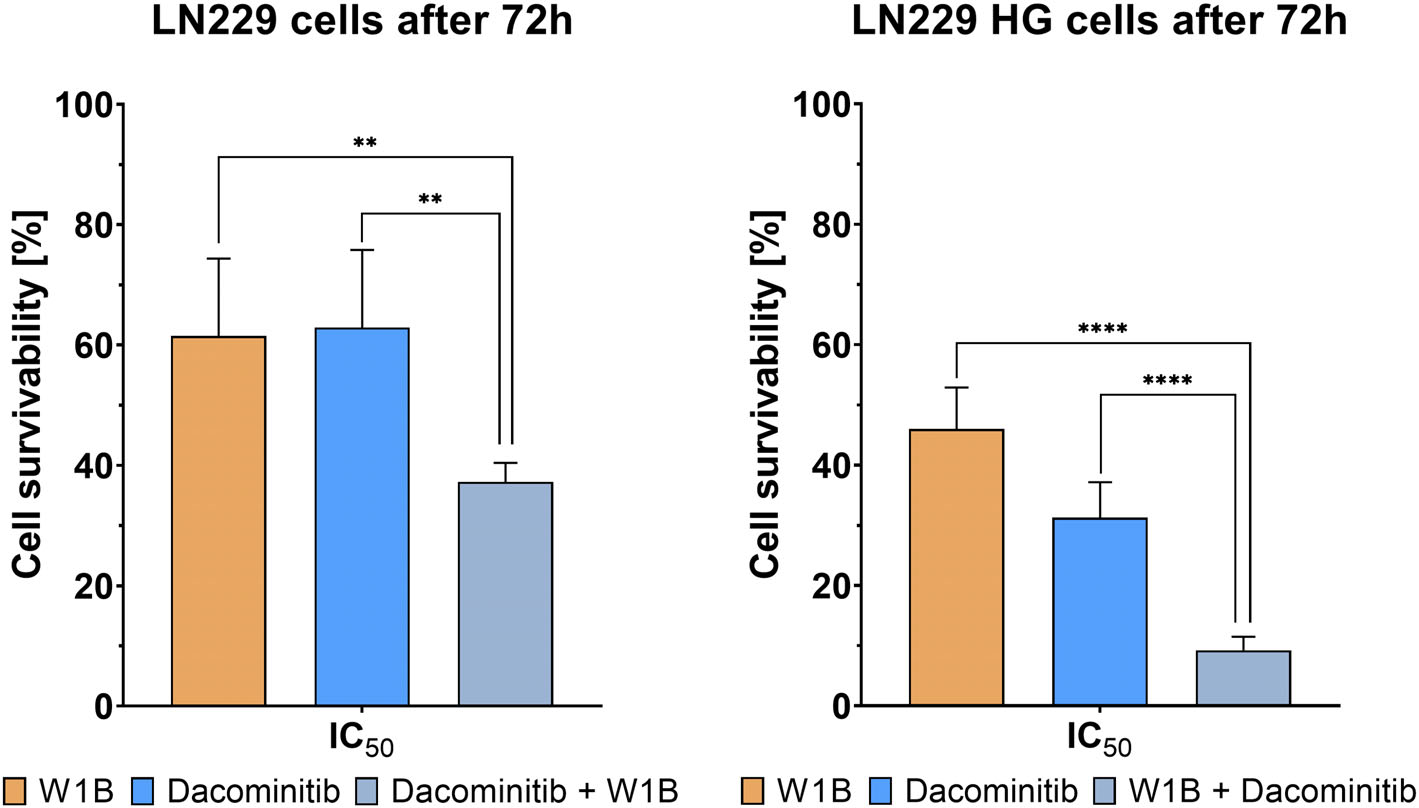
Survivability of LN229 and LN229 high‐glucose (HG) cells following treatment with W1B, dacomitinib, or their combination. Data are presented as mean ± SD (*n* = 6) and were analyzed using two‐way ANOVA with Tukey's post hoc test (**P < 0.01, ****P < 0.0001).

As the results were so interesting, we decided to verify the synergistic action more quantitatively. The efficacy of the therapy was assessed at its final stage (after 72 h) using a cytotoxicity assessment method. The results presented in Fig. 8 show a significant reduction in cell survival after combination therapy under standard conditions and conditions of increased glucose concentration. Supporting these observations, the combination yielded CI values of 0.94 in the LN229 cells and an even stronger synergistic score of 0.87 in the LN229 HG variant (Table S3 and Fig. S10).

### 3.7 *In silico* determination of BBB permeation

To pre‐evaluate the ability of the novel quinazolinone derivative to cross the BBB, the *in silico* pharmacokinetic analysis was performed using ACD/Percepta software. The calculated parameters included BBB penetration (logBB), passive diffusion (logPS), brain‐plasma equilibrium (logPS*_fu,brain_), and the unbound fractions in plasma (Fu) and brain tissue (Fb). These descriptors provide an initial indication of CNS pharmacokinetics and are summarized in Table 2.

**Table 2:** The BBB Pharmacokinetic Descriptors of W1B Calculated *in silico* (ACD/Percepta software).

Log BB	Log PS	LogPS*_fu,brain_	Fu	Fb
−0.87	−1.1	−3.1	0.0016	0.01

In our study, W1B had very low unbound fractions in both brain (Fb = 0.0016) and plasma (Fu = 0.11), while the equilibrium rate between brain and plasma (logPS‐fu, brain = −3.1) was relatively high.

To further refine BBB permeability predictions, additional physicochemical properties were assessed using the Hansch method (see Table 3). Steric, electronic, and hydrophobic descriptors were calculated, since molecular size, shape, and lipophilicity are critical for both target binding and membrane permeation. Lipophilicity was quantified by partition coefficients between n‐octanol and water (logPow) and between cyclohexane and water (logPcw), which provide complementary insights into solvation and membrane affinity.

**Table 3:** Physicochemical parameters calculated *in silico*.

LogPow	LogPcw	MW [g·mol^−1^]	TPSA [Å^2^]
4.206	1.717	452.91	93.21

W1B, with a molecular weight of 452.9 g·mol^−1^, exhibits pronounced polarizability, as well as has a topological polar surface area (TPSA) of 93.2 Å^2^ and an n‐octanol/water partition coefficient (logPow) calculated as 4.206.

### 3.8 Biomimetic studies on the lipophilicity

Lipophilicity is a key determinant of CNS accessibility, given its decisive role in barrier penetration and systemic distribution. To experimentally assess this property for W1B, we employed non‐cell‐based biomimetic *in vitro* studies as a model of passive transport and absorptive behavior across biological membranes. Specifically, high‐performance liquid chromatography (HPLC) with an immobilized artificial membrane (IAM) system was used.

Chromatographic experiments were performed using an IAM.PC.DD2 column as the stationary phase, while buffered acetonitrile solutions served as mobile phases (0.4, 0.5, 0.6, 0.7% v/v acetonitrile/buffer; pH = 7.4). The logarithm of retention factors extrapolated to a purely aqueous mobile phase (log kw) was calculated according to the Soczewinski–Wachtmeister Eq. [1]:

(1) logk=logkw−sφ

where log *k* is the logarithm of the retention factor measured in the mixed aqueous–organic effluent system, *φ* is the volume fraction of the organic modifier, and *s* is the solute‐specific slope characteristic for the chromatographic system.

Application of this model yielded an extrapolated log kw value of 2.533 for W1B, with a solute‐specific slope *s* = 4.787 and an excellent correlation coefficient (*R* ^2^ = 0.98), confirming the robustness of the linear fit.

### 3.9 *In vivo* studies

**Fig. 9:**
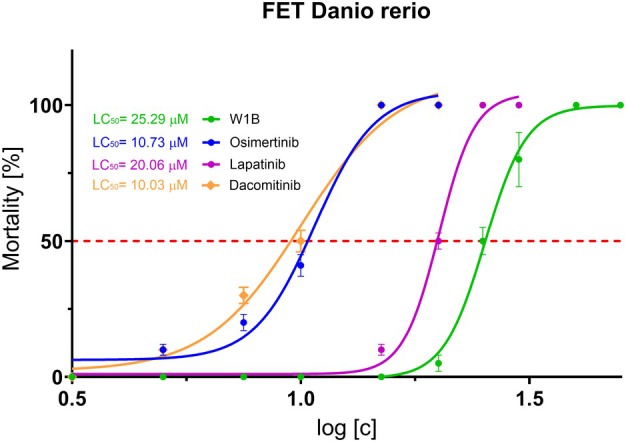
Toxicity of W1B, osimertinib, lapatinib, and dacomitinib showed as mortality rate [%] ± SD of zebrafish embryos (Danio rerio) exposed to a range of concentrations: 0–50 μm. Calculated values of LD_50_ for W1B, osimertinib, lapatinib, and dacomitinib: 25.29, 10.73, 20.06, and 10.03 μm respectively.

W1B displayed a safety profile comparable to lapatinib and clearly superior to osimertinib and dacomitinib (Fig. 9). The LC_50_ values obtained from the zebrafish embryo acute toxicity assay were 25.29 μm for W1B, 20.06 μm for lapatinib, 10.73 μm for osimertinib, and 10.03 μm for dacominitib.

**Fig. 10:**
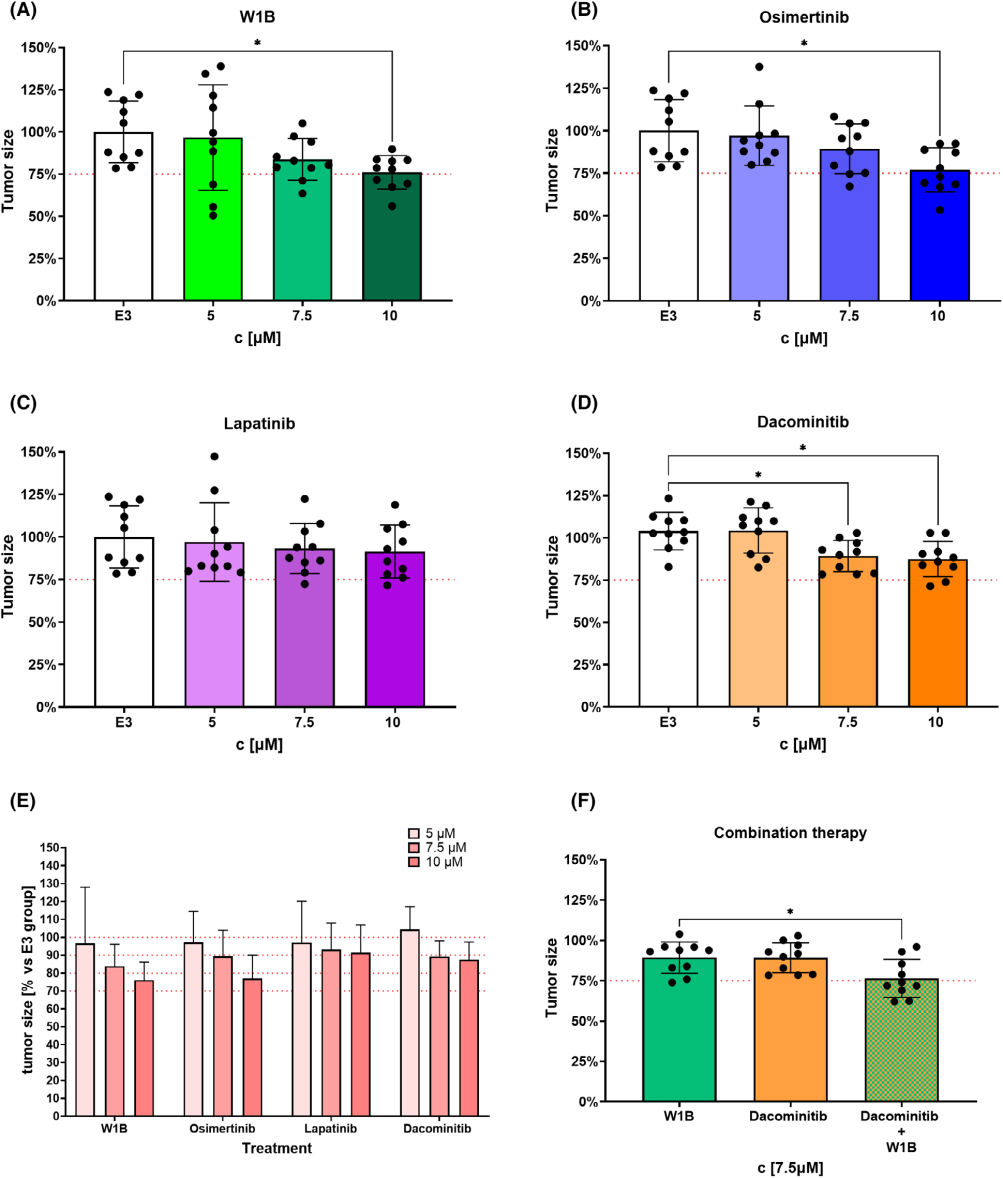
Antitumor effectiveness of W1B, osimertinib and lapatinib in Danio rerio xenograft model evaluated as a percentage of tumor area measurements for the LN229 xenograft after 72 h of incubation with tested compounds: (A) W1B, (B) osimertinib, (C) lapatinib, (D) dacominitib. (E) Comparison of treatment efficacy at three different concentrations: 5, 7.5, and 10 μm of applied antitumor substances, presented as percentage of tumor size versus the untreated control group (E3) (F) co‐treatment of W1B and dacominitib at the dose of 7.5 μm. Data are presented as mean ± SD (*n* = 4) and were analyzed using one‐way ANOVA; *P < 0.05.

In terms of therapeutic efficacy, W1B demonstrated strong antitumor activity in xenografted zebrafish embryos, with outcomes consistent with prior *in vitro* data (Fig. 10A–D). Tumor growth was significantly reduced at all tested concentrations, with the most pronounced effect observed at the highest dose. At this concentration, tumor size was decreased to 76.09% in the W1B‐treated group and to 76.97% in the osimertinib group and 87.42% in case of dacomitinib treatment, whereas lapatinib‐treated embryos showed only a modest reduction to 91.44% (Fig. 10E). These results are in agreement with the antiproliferative activity observed *in vitro*, where W1B exhibited a potent effect (IC_50_ = 3.57 ± 1.03 μm), superior to all of them: osimertinib (IC_50_ = 8.99 ± 0.37 μm), dacomitinib (IC_50_ = 5.84 ± 0.17 μm) and lapatinib (IC_50_ = 12.60 ± 0.32 μm). Notably, at 7.5 μm, the combination of W1B and dacomitinib achieved an antitumor effect superior to that observed for either compound alone. The selection of the 7.5 μm dose for both compounds was dictated by the toxicity profile of dacomitinib (LC_50_ = 10.03 μm). Consequently, this combination demonstrates efficacy at sub‐10 μm concentrations while maintaining a favorable toxicity profile (Fig. 10F).

## 4 Discussion

The therapeutic management of glioblastoma is severely compromised by the tumor's intrinsic molecular complexity and the restrictive properties of the BBB. Consequently, developing novel small molecules with favorable physicochemical profiles for CNS penetration that selectively target tumor proliferation is of paramount importance. In evaluating the antiproliferative spectrum of our synthesized compounds, the robust activity of the W1B derivative against both GBM and specific epithelial models is of particular interest. Our findings highlight a stringent structure–activity relationship within this novel class of styrylquinazolinones. The striking potency discrepancy between W1B and its inactive analogues underscores that the specific combination of a 7‐chloroquinazoline scaffold with a tosyl group substituted at position 3 of the styryl moiety is critical for conferring significant biological activity. Beyond brain tumors, we expanded our evaluation to include the PC‐9 cell line, an EGFR‐mutant non‐small cell lung cancer (NSCLC) model. Notably, NSCLC has a well‐documented clinical propensity for brain metastasis, making PC‐9 a highly relevant complementary model in the context of our brain‐targeted, glioblastoma‐oriented research. The fact that W1B's activity against PC‐9 cells was comparable to that observed in standard GBM models (U‐251 and U87MG) underscores its potential against malignancies capable of penetrating the CNS. Furthermore, the comparison of W1B with clinically utilized EGFR/HER2 inhibitors highlights its favorable safety profile. While osimertinib showed limited selectivity (TIs below 0.8), a characteristic consistent with the profile of many clinically approved anticancer agents. In contrast, W1B demonstrated a superior safety margin for normal tissues. Thus, the promising antiproliferative activity and favorable selectivity of W1B prompted us to explore further its inhibitory effects on several RTKs relevant to GBM.

RTK signaling pathways represent the most commonly dysregulated signaling networks in GBM, with alterations occurring in nearly 90% of tumors [51]. These aberrations, arising from both mutations and gene amplification, lead to receptor overexpression and constitutive activity, thereby highlighting them as key therapeutic targets. Among these, the most prominent are EGFR, IGF1R, PDGFR, and VEGFR. The enzymatic profiling of W1B revealed a distinct selectivity profile, demonstrating preferential activity against specific RTKs. Most notably, W1B exhibited potent inhibitory activity against IGF1R, decreasing its activity by more than 74%. This marked reduction in enzymatic activity in the presence of W1B is indicative of its mechanism as a competitive inhibitor, efficiently engaging the ATP‐binding site and thereby impeding the phosphorylation process required for receptor activation. This robust target engagement was further corroborated by MST analysis, which yielded a submicromolar dissociation constant (*K*_d_ = 598 nm). Such a value highlights a strong and specific interaction between W1B and IGF1R, further supporting its potential as a promising inhibitor. However, it should be emphasized that the full utility of the compound will be established through cellular studies, specifically by evaluating its ability to inhibit receptor‐mediated signaling pathways.

Our *in silico* studies provided deep structural insights into the interaction profile of W1B. All calculated binding energies were substantial, indicating strong ligand–protein affinity and effective occupancy of the binding pocket. The slight variability in binding energies between alternative structures of the same proteins (such as IGF1R and EGFR) indicates non‐negligible differences in the conformation of the protein's backbone. These results are partially consistent with experimental data; however, they may not fully capture the influence of the complete protein environment or the differences in the inhibitory potency and behavior of W1B toward EGFR and IGF1R.

A critical observation from the structural analysis is the ligand's ability to adopt two distinct poses depending on the target protein. This divergent structural pattern can be rationalized by specific sequence variations within the binding pockets. The structural differences in the ligand–protein complex are qualitatively explained by changes in the interaction characteristics between individual amino acid residues and the W1B. In particular, the presence of Leu858 in EGFR enables CH–π interactions with the aromatic–aliphatic portion of W1B, thereby stabilizing its extended conformation. In contrast, the absence of this interaction in the other structures favors a folded conformation of the ligand. Additionally, regarding changes in the backbone conformation, the most significant structural difference in the proteins appears to be the deviation of the loop connecting the β‐strands around Phe1010 (the equivalent in PDB: 1M17 is Phe723). This change allows energetically favorable π‐π interactions with the phenyl rings of W1B in the case of EGFR. In the other structures, the analogous residues are shifted away from the binding pocket and do not contact the ligand, while the space left unoccupied by Phe is instead filled by a fragment of W1B in its folded conformation. In addition, the main driving force for binding is the CH–π interactions involving a cluster of nonpolar residues (Val, Leu, and Ala), present in the binding pocket of every considered protein, with the quinazoline scaffold of the W1B. However, the final arrangement of the other fragments of the ligand in the pocket and its conformation strongly depend on the primary structure of the protein.

At the cellular level, activation of IGF1R and EGFR initiates signaling cascades through Akt and RAS proteins, ultimately modulating mTOR expression and regulating key cellular processes such as metabolism, proliferation, growth, survival, and motility. To investigate the mechanism underlying the action of W1B, the protein levels of IGF1R and EGFR, and their downstream signaling elements, including Akt, p‐Akt, RAS, as well as two mTOR‐regulated proteins: p70 S6 kinase and p‐S6 Ribosomal protein were evaluated. Our analysis demonstrated that W1B effectively downregulates both receptors in LN229 cells, leading to a potent inhibition of Akt activation. Interestingly, two downstream effectors of mTOR signaling displayed an interesting expression pattern in response to treatment. First was p70 S6K, a direct substrate of mTORC1, which cooperates with it to augment protein synthesis, ribosomal biogenesis, and stimulate overall anabolic processes [52]. It is also worth mentioning that full activation of mTORC1 requires the presence of amino acids and insulin stimulation [53]. Our analysis demonstrated a significant reduction in the p70 S6K abundance when GBM cells were treated with W1B at 2.3 μm. In contrast, treatment with the higher concentration (3.5 μm) led to an almost 1.9‐fold increase compared to untreated cells. A possible explanation for this nonlinear protein response is the differential phosphorylation kinetics at multiple activation sites. This behavior may reflect the fact that S6K activity and subcellular localization are modulated in response to diverse stimuli [54]. In addition, the p70 S6K may contribute to PI3K/Akt/mTOR feedback loop regulation by suppressing mTORC2 complex and PDK1 activity, thereby leading to Akt inhibition [54, 55, 56]. Conversely, the opposite situation was registered for the second analyzed protein – phosphorylated S6 Ribosomal protein, which is also involved in the regulation of protein synthesis, ribosome biogenesis, and cell growth downstream of the mTORC1 signaling pathway. Indeed, W1B significantly reduced the protein level at 3.5 μM concentration (almost 1.6‐fold), whereas the lower concentration enhanced protein expression. The last protein analyzed was RAS, an alternative signaling mediator downstream of EGFR and IGF1R activation. Within the RAS pathway, ERK and MEK phosphorylate numerous substrates to regulate cellular responses, including promoting proliferation and differentiation while inhibiting apoptosis. RAS can also bind to the p110 subunit of PI3K, thereby facilitating the activation of Akt [57]. Notably, W1B treatment had minimal effect on RAS levels, suggesting that this pathway is not involved in the cellular response. Collectively, these findings indicate that the novel derivative exerts a strong inhibitory effect on kinase signaling at the cellular level.

Importantly, GBM cells are characterized by reprogrammed metabolism that supports their rapid proliferation, division, and resistance to therapy. A hallmark of this metabolic shift is the increased uptake of glucose and elevated lactate production, even under normoxic conditions. This phenomenon is known as aerobic glycolysis, or the Warburg effect, which provides both energy and biosynthetic precursors to sustain tumor growth. In addition, glucose and acetate have been shown to promote EGFRvIII signaling through mTORC2 in GBM [58]. Elevated levels of pyruvate kinase M2, the enzyme catalyzing the final step of glycolysis, have also been reported in GBM and correlate with enhanced EGFR activity and increased glioma malignancy [59]. Bao *et al*. also showed that high glucose levels increase the activity of GPCR and EGFR receptors, promoting the migration and growth of GBM cells [60]. Another report revealed that EGFR participates in and facilitates glucose transport into cells by binding to and stabilizing sodium/glucose cotransporter 1 (SGLT1) [61, 62]. Our experiments showed that culturing LN229 cells in a high‐glucose environment (LN229 HG) diminished the potency of W1B, increasing the IC_50_ value to 34.4 μm. In addition, the EGFR expression remained high in glucose‐driven GBM cells after exposure to W1B. This lack of EGFR susceptibility is consistent with the findings of Ren *et al*., who postulated that modulation of EGFR activity does not disrupt its interaction with SGLT1 in prostate cancer [63]. Notably, 23 μm and 35 μm of W1B caused a significant, almost 1.5‐fold reduction in IGF1R levels in LN229 HG. These findings indicate that W1B effectively binds to and inhibits IGF1R activity. However, further western blot analysis confirmed the upregulation of the Akt/mTOR signaling pathway, as evidenced by increased levels of p‐Akt and p70 S6K. This effect was likely driven by crosstalk between insulin‐like receptors and EGFR, reflecting an intracellular compensatory mechanism that is crucial for the efficacy of treatment. Thus, inhibition of IGF1R by W1B and its effect on downstream targets may be counteracted by a robust EGFR‐driven activation signal. In addition, Akt activation may govern glucose uptake by directing GLUT expression and trafficking to the cell surface [64], which may also maintain EGFR expression. Moreover, RAS abundance was elevated in LN229 HG cells after treatment with W1B. Such an increase likely reflects a known compensatory feedback mechanism [65], whereby partial inhibition of tyrosine receptors leads to transcriptional upregulation of RAS, enabling downstream signaling to persist despite blockade.

Comparing standard and elevated glucose conditions revealed distinct differences in W1B's efficacy against two key RTKs. While enhanced, glucose‐driven cellular metabolism significantly attenuated the compound's inhibition of EGFR, its ability to block IGF1R remained entirely unaffected. This finding corroborates our earlier data from isolated kinase assays, where W1B also exhibited its most potent inhibitory activity against IGF1R. Our strategy for investigating the reduced potential of W1B to inhibit EGFR under conditions of elevated metabolism was to utilize dacomitinib—a quinazoline‐based irreversible inhibitor of the ERBB family of kinases, including EGFR, ERBB2 (HER2), and ERBB4 (HER4). Our combination therapy data revealed that the simultaneous administration of W1B and dacomitinib not only preserves the strong suppression of IGF1R but also successfully restores and markedly enhances the inhibition of EGFR activity in the high‐glucose model. The quantitative assessment of cytotoxicity further confirmed this interaction, revealing clear synergy (CI = 0.87), particularly in the metabolically active LN229 HG cells.

Collectively, these data demonstrate the high potential of W1B, not only as an IGF1R inhibitor in monotherapy, but also as an EGFR inhibitor in combination therapy, even under conditions of increased cellular metabolism. These findings are promising and warrant further research, especially in the context of overcoming metabolic‐driven drug resistance and developing robust, multi‐targeted therapy against GBM.

Our further analyses focused on predicting the ability of W1B to cross the BBB. Among the *in silico* parameters used to determine of BBB permeation, logBB is the most widely applied predictor, reflecting the steady‐state distribution ratio between brain and blood. Compounds with logBB ≥ 0.3 are considered readily permeable, −1 < logBB < 0.3 moderately permeable, and logBB < −1 poorly permeable [66, 67]. Based on these thresholds, the logBB value of −0.87 determined *in silico* indicates that W1B is only able to cross an intact BBB to a limited extent. However, given that glioblastoma is often associated with focal BBB disruption, this limited penetration could still allow meaningful drug exposure within tumor regions. Although these computational predictions are encouraging, experimental validation in relevant *in vitro* or *in vivo* models is necessary. In addition to the overall penetration of the BBB, the rate at which a substance equilibrates between plasma and brain tissue is influenced by its permeability surface area product (PS) and the unbound fraction in the brain (Fb). When PS or Fb values are low, distribution equilibrium in the CNS is delayed [68]. For a drug to exert its pharmacological effect, only the unbound fraction in the bloodstream is pharmacologically active, although drugs can also associate with plasma proteins and erythrocytes. These principles are fundamental to the free drug hypothesis, which states that distribution in tissue is primarily determined by the concentration of the unbound drug [69, 70]. The combination of the relatively high equilibrium rate and very low unbound fractions suggests that W1B is able to efficiently penetrate brain tissue despite the low unbound fraction, probably due to favorable permeability (PS) and limited binding in the brain. Together with the logBB value, these data indicate that W1B has at least partial CNS accessibility, supporting its potential as a candidate for glioblastoma therapy.

Based on physicochemical profiling, W1B can be classified as a highly lipophilic molecule with the capacity to cross biological barriers, including the BBB. According to the Hansch framework, membrane permeability is determined not only by lipophilicity but also by steric and electronic factors. A key predictor of CNS penetration is the topological polar surface area (TPSA), which reflects the number of polar atoms that can form hydrogen bonds. Compounds with TPSA < 90 Å^2^ are generally favored for passive BBB diffusion. W1B has a TPSA of 93.2 Å^2^, slightly above this threshold; however, its pronounced lipophilicity may compensate for this limitation. Moreover, the localized BBB disruption commonly observed in glioblastoma could further facilitate drug entry into tumor tissue.

It is important to emphasize that the ability of a drug to cross the BBB is largely determined by its physicochemical properties, with lipophilicity playing a central role alongside potential contributions from active transport mechanisms [71]. The lipophilic properties influence not only the distribution in the membrane, but also the interactions with competing binding sites and metabolizing enzymes. As hydrophobic interactions are crucial for molecular recognition and transport, lipophilicity is usually quantified by partition coefficients measured in different experimental systems [72]. Given its decisive role in barrier penetration and systemic distribution, lipophilicity is a key determinant of CNS accessibility. To complement *in silico* predictions, we utilized HPLC with IAM system, which is consists of a monolayer of phosphatidylcholine covalently bound to a propylamino‐silicate carrier and is widely recognized as a robust, non‐cell‐based *in vitro* approach. The permeability of a substance through biological barriers is largely determined by its membrane partition coefficient, which is difficult to determine directly *in vivo* [73]. The IAM stationary phases are designed to mimic the amphiphilic character of biological membranes, providing a suitable model for assessing passive membrane partitioning and the interactions of compounds with phospholipid‐like environments. This approach yields an experimentally robust estimate of a molecule's ability to traverse lipid bilayers, thereby complementing *in silico* predictions and highlighting the central role of lipophilicity in CNS penetration. Although the extrapolated log kw (2.533) is slightly lower than the calculated logPow (4.206), reflecting the distinct experimental setup, both measures consistently support the high lipophilicity of W1B, reinforcing its potential to efficiently cross biological membranes. Although these predictive and biomimetic findings are very promising, they ultimately require experimental validation in complex biological models. A major limitation of *in silico* modeling and non‐cell‐based IAM systems is their inability to account for active efflux transporters, such as P‐gp or BCRP, which heavily restrict CNS entry for many lipophilic drugs. Therefore, the actual brain penetrance of W1B warrants assessment using cell‐based *in vitro* BBB models (e.g., Transwell assays) or pharmacokinetic profiling in mouse models. Should passive diffusion prove insufficient under physiological conditions, the delivery of W1B could be supported by advanced formulation strategies, such as encapsulation in nanocarriers or liposomes, to ensure effective therapeutic concentrations within the brain.

The translation of *in vitro* efficacy to *in vivo* models is a critical step in preclinical drug development. Our zebrafish embryo assays confirmed that W1B possesses a highly favorable safety profile, allowing for administration at higher concentrations without inducing significant toxicity. This indicates a broader therapeutic window for W1B compared to osimertinib and dacomitinib, a finding of particular clinical importance, as systemic toxicity frequently represents a limiting factor in the application of small‐molecule kinase inhibitors. In terms of therapeutic efficacy, the robust antitumor activity of W1B in the xenograft model strongly corroborates our earlier *in vitro* findings. The potent *in vivo* tumor reduction (to 76.09%) mirrors the superior *in vitro* antiproliferative effect of W1B (IC_50_ = 3.57 μm) when compared to osimertinib (IC_50_ = 8.99 μm), dacomitinib (IC_50_ = 5.84 μm), and lapatinib (IC_50_ = 12.60 μm). While lapatinib demonstrated a toxicity profile similar to W1B, it was markedly less effective in both experimental settings. Conversely, osimertinib and dacomitinib, despite showing comparable or slightly lower efficacy, are associated with significantly greater toxicity. Importantly, the combination of W1B and dacomitinib at 7.5 μm concentrations demonstrated an enhanced, synergistic antitumor effect without an accompanying increase in toxicity. This suggests a modest synergistic therapeutic effect and non‐overlapping toxicity profiles between the two agents, reinforcing the rationale for our dual‐targeted strategy.

Taken together, the concordance between *in vitro* and *in vivo* findings supports the robustness of W1B's anticancer activity. By offering an advantageous balance between safety and efficacy, W1B emerges as a highly promising therapeutic candidate, and its consistent performance across different experimental models provides a strong rationale for advancing it into further preclinical investigations.

## 5 Conclusion

The study identified W1B, a novel styrylquinazolinone‐based small‐molecule with potent antiproliferative activity against GBM cells. W1B displayed strong inhibition of IGF1R kinase activity, with additional effects on EGFR and HER2, to a lesser extent. Microscale thermophoresis confirmed high binding affinity toward IGF1R, while molecular docking predicted favorable binding energies and different binding poses within IGF1R and EGFR. Importantly, W1B exerted robust dual receptor inhibition in cellular GBM models and attenuated signaling along the Akt/mTOR axis. Under high‐glucose conditions, which more closely reflect the reprogrammed metabolism of GBM, the compound retained selective IGF1R inhibition, although a compensatory mechanism was observed that enhanced activation of the Akt pathway. Combination studies under high‐glucose conditions revealed a pronounced synergistic effect with the EGFR inhibitor dacomitinib, underscoring the therapeutic relevance of simultaneously targeting IGF1R and EGFR. Notably, co‐administration of both inhibitors led to a marked reduction in receptors' activity in glucose‐driven GBM cells. The presented measurements of biomimetic lipophilicity showed that W1B has the potential to penetrate biological membranes, confirming its ability to affect the CNS. Finally, *in vivo* studies on the zebrafish xenograft model confirmed the high anticancer potential of W1B and its favorable safety profile. Given its structural novelty and promising activity profile, W1B represents a valuable lead for the development of multifunctional IGF1R‐targeted therapies in GBM. Moreover, W1B demonstrates strong potential for combination therapy, enhancing therapeutic efficacy without compromising the safety profile.

## Conflict of interest

The authors declare no conflict of interest.

## Author contributions

PR performed microscale thermophoresis, combination therapy, immunoblotting studies, and wrote the text; WC designed and synthesized compounds and wrote the chemistry part; WP performed and analyzed the molecular docking; KS performed and analyzed biomimetic experiments; ABC and EK performed and analyzed *in vivo* studies; RM supervised MJ; MJ synthesized compounds; AMW discussed the results and wrote the manuscript; KM created the research hypothesis, designed the biological experiments, performed the cytotoxicity and kinase inhibition studies, discussed the results, and wrote the manuscript. All the authors read and approved the final version of the manuscript.

## Supporting information


**Fig. S1.** ^1^H NMR and ^13^C NMR data for compound W1A.
**Fig. S2.** ^1^H NMR and ^13^C NMR data for compound W1B.
**Fig. S3.** ^1^H NMR and ^13^C NMR data for compound W1C.
**Fig. S4.** ^1^H NMR and ^13^C NMR data for compound W2B.
**Fig. S5.** HRMS data for compound W1A.
**Fig. S6.** HRMS data for compound W1B.
**Fig. S7.** HRMS data for compound W1C.
**Fig. S8.** HRMS data for compound W2B.
**Fig. S9.** Validation of the adopted docking protocol: comparison of crystallographic poses (colors correspond to atom types) and poses obtained by docking (pink colors). Protein structures from PDB: 1K3A (IGF1R), 1 M17 (EGFR), 2ZM3 (IGF1R), 3EKN (InsR), and 4HJO (EGFR).
**Fig. S10.** Synergistic effect of W1B and dacomitinib combination therapy in LN229 and LN229 HG cells. Dose–response curves illustrating the effect of W1B, dacomitinib, and their combination and polygonogram graphically representing the drug interactions.
**Fig. S11.** Densitometric analyses of protein expression. Relative band intensities were normalized to the reference protein (GAPDH, vinculin, cyclophilin β, or cofilin) and expressed as fold‐change relative to untreated control cells. Data are presented as mean ± SD (*n* = 6) and were analyzed using one‐way ANOVA with Dunnett's post hoc test (*P < 0.1, **P < 0.01, ***P < 0.001, ****P < 0.0001).
**Fig. S12.** The uncropped western blots treated LN229 and LN229 HG cell lines with W1B compound in 3 different concentrations.
**Fig. S13.** The uncropped western blots of LN229 HG cells treated with W1B compound, dacomitinib, and their combination.
**Table S1.** Antiproliferative activity of the tested compounds against MCF‐7 and PC‐9 cells.
**Table S2.** Inhibition profile of W1B against non‐receptor tyrosine kinases.
**Table S3.** Combination Index (CI) values shown at fraction affected (Fa) for W1B and Dacomitinib combination in LN229 and LN229 HG cell lines.

## Data Availability

Data supporting the findings of this study are available within the article. Source data are available from the corresponding author on request (katarzyna.malarz@us.edu.pl).
