# Supplementary material for: A novel quinazolinone insulin receptor inhibitor and its synergy with an EGFR inhibitor in glucose‐driven glioblastoma
Source: Mol Oncol. 2026 May 27:10.1002/1878-0261.70274. Online ahead of print. doi: 10.1002/1878-0261.70274 (PMC13398722; doi:10.1002/1878-0261.70274)
Supplement: Supplementary file 1 — Fig. S1. 1H NMR and 13C NMR data for compound W1A. Fig. S2. 1H NMR and 13C NMR data for compound W1B. Fig. S3. 1H NMR and 13C NMR data for compound W1C. Fig. S4. 1H NMR and 13C NMR data for compound W2B. Fig. S5. HRMS data for compound W1A. Fig. S6. HRMS data for compound W1B. Fig. S7. HRMS data for compound W1C. Fig. S8. HRMS data for compound W2B. Fig. S9. Validation of the adopted docking protocol: comparison of crystallographic poses (colors correspond to atom types) and poses obtained by docking (pink colors). Protein structures from PDB: 1K3A (IGF1R), 1 M17 (EGFR), 2ZM3 (IGF1R), 3EKN (InsR), and 4HJO (EGFR). Fig. S10. Synergistic effect of W1B and dacomitinib combination therapy in LN229 and LN229 HG cells. Dose–response curves illustrating the effect of W1B, dacomitinib, and their combination and polygonogram graphically representing the drug interactions. Fig. S11. Densitometric analyses of protein expression. Relative band intensities were normalized to the reference protein (GAPDH, vinculin, cyclophilin β, or cofilin) and expressed as fold‐change relative to untreated control cells. Data are presented as mean ± SD (n = 6) and were analyzed using one‐way ANOVA with Dunnett's post hoc test (*P < 0.1, **P < 0.01, ***P < 0.001, ****P < 0.0001). Fig. S12. The uncropped western blots treated LN229 and LN229 HG cell lines with W1B compound in 3 different concentrations. Fig. S13. The uncropped western blots of LN229 HG cells treated with W1B compound, dacomitinib, and their combination. Table S1. Antiproliferative activity of the tested compounds against MCF‐7 and PC‐9 cells. Table S2. Inhibition profile of W1B against non‐receptor tyrosine kinases. Table S3. Combination Index (CI) values shown at fraction affected (Fa) for W1B and Dacomitinib combination in LN229 and LN229 HG cell lines. [file MOL2-9999-0-s001.docx]

**A novel quinazolinone insulin receptor inhibitor and its synergy with an EGFR inhibitor in glucose-driven glioblastoma**

**Patryk Rurka^1^, Wioleta Cieślik^2^, Wojciech Płaziński^3,4^, Katarzyna Stępnik^5^, Anna Boguszewska-Czubara^6^, Elżbieta Kot^6^, Robert Musioł^2^, Mateusz Jasica^2^, Anna Mrozek-Wilczkiewicz^1,7^, Katarzyna Malarz^1*^**

^1^Institute of Physics, University of Silesia in Katowice, 75 Pułku Piechoty 1a, 41-500 Chorzów, Poland

^2^Institute of Chemistry, University of Silesia in Katowice, 75 Pułku Piechoty 1a, 41-500 Chorzów, Poland

^3^Jerzy Haber Institute of Catalysis and Surface Chemistry, Polish Academy of Sciences, Niezapominajek 8, Kraków, 30-239, Poland

^4^Department of Biopharmacy, Medical University of Lublin, Chodźki 4a, 20-093 Lublin, Poland

^5^Department of Physical Chemistry, Institute of Chemical Sciences, Maria Curie–Skłodowska University in Lublin, Pl. M. Curie-Skłodowskiej 3, Lublin, 20-031, Poland

^6^Department of Medical Chemistry, Medical University of Lublin, Chodźki 4a, 20-093 Lublin, Poland

^7^Department of Systems Biology and Engineering, Silesian University of Technology, Akademicka 16, 44-100 Gliwice, Poland

*corresponding author: [katarzyna.malarz@us.edu.pl](mailto:katarzyna.malarz@us.edu.pl)

**Table of contents:**

[1. Chemistry 2](#_Toc228221764)

[2. Representative spectra 5](#_Toc228221765)

[2.1. ^1^H NMR and ^13^C NMR spectra 5](#_Toc228221766)

[2.2. HRMS spectra 9](#_Toc228221767)

[3. Antiproliferative activity against epithelial cancer cells 13](#_Toc228221768)

[4. Non-receptor tyrosine kinase inhibition studies 13](#_Toc228221769)

[5. Molecular docking 13](#_Toc228221770)

[6. Combinational Therapy 14](#_Toc228221771)

[7. Western Blot 15](#_Toc228221772)

[7.1. Densitometric analyses 15](#_Toc228221773)

[7.2. Raw data 16](#_Toc228221774)

# Chemistry

**2-formylphenyl 4-methylbenzenesulfonate (A).** The compound was obtained using general procedure **2.1.1.;** resulting in a white powder with a yield of 69 % (950 mg); **^1^H NMR** (500 MHz, CDCl_3_-*d*) δ 10.01 (t, *J* = 0.8 Hz, 1H), 7.89 (dd, *J* = 7.7, 1.8 Hz, 1H), 7.73 (d, *J* = 8.1 Hz, 2H), 7.61 (ddd, *J* = 8.2, 7.3, 1.8 Hz, 1H), 7.42 (td, *J* = 7.5, 1.0 Hz, 1H), 7.36 (d, *J* = 8.1 Hz, 2H), 7.24 (dt, *J* = 8.2, 0.9 Hz, 1H), 2.48 (s, 3H). **^13^C NMR** (126 MHz, CDCl_3_-*d*) δ 187.34, 151.24, 146.32, 135.32, 131.35, 130.14, 129.28, 128.65, 128.51, 127.54, 123.76, 21.79.

**3-formylphenyl 4-methylbenzenesulfonate (B).** The compound was obtained using general procedure **2.1.1**.; resulting in a beige powder with a yield of 65 % (900 mg); **^1^H NMR** (500 MHz, CDCl_3_-*d*) δ 9.95 (s, 1H), 7.80 (dt, *J* = 7.6, 1.3 Hz, 1H), 7.75 – 7.72 (m, 2H), 7.53 – 7.48 (m, 2H), 7.35 (dt, *J* = 7.3, 0.9 Hz, 2H), 7.32 (ddd, *J* = 8.1, 2.5, 1.1 Hz, 1H), 2.48 (s, 3H); **^13^C NMR** (126 MHz, CDCl_3_-*d*) δ 190.62, 150.17, 145.86, 137.85, 131.98, 130.44, 129.95, 128.49, 128.43, 128.24, 123.07, 21.75.

**4-formylphenyl 4-methylbenzenesulfonate (C).** The compound was obtained using general procedure **2.1.1.;** resulting in a white powder with a yield of 44 % (620 mg); **^1^H NMR** (500 MHz, CDCl_3_-*d*) δ 9.99 (s, 1H), 7.89 – 7.82 (m, 2H), 7.76 – 7.67 (m, 2H), 7.39 – 7.32 (m, 2H), 7.23 – 7.18 (m, 2H), 2.48 (s, 3H); **^13^C NMR** (126 MHz, CDCl_3_-*d*) δ 190.67, 153.88, 145.90, 134.81, 132.04, 131.28, 129.96, 128.47, 123.09, 21.76.

**7-chloro-2-methylquinazolin-4(3*H*)-one (W1).** The compound was obtained using general procedure **2.1.2.;** resulting in a white powder with a yield of 37 % (1,7 g); mp 266-267 ^o^C; **^1^H NMR** (500 MHz, DMSO-*d*_6_) δ 12.28 (s, 1H), 8.05 (d, *J* = 8.5 Hz, 1H), 7.61 (d, *J* = 2.0 Hz, 1H), 7.47 (dd, *J* = 8.5, 2.1 Hz, 1H), 2.35 (s, 3H); **^13^C NMR** (126 MHz, DMSO-*d*_6_) δ 161.58 , 156.52 , 150.54 , 139.31 , 128.25 , 126.60 , 126.17 , 119.94 , 21.96 .

**8-chloro-2-methylquinazolin-4(3*H*)-one (W2).** The compound was obtained using general procedure **2.1.2.;** resulting in a white powder with a yield of 76 % (3,5 g); mp 247-250 ^o^C; **^1^H NMR** (500 MHz, DMSO-*d*_6_) δ 12.44 (s, 1H), 8.03 (dd, *J* = 7.9, 1.5 Hz, 1H), 7.92 (dd, *J* = 7.8, 1.4 Hz, 1H), 7.42 (t, *J* = 7.8 Hz, 1H), 2.40 (s, 3H); **^13^C NMR** (126 MHz, DMSO-*d*_6_) δ 161.68, 155.96, 145.80, 134.87, 130.58, 126.61, 125.33, 122.85, 22.24.

**(E)-2-(2-(7-chloro-4-oxo-3,4-dihydroquinazolin-2-yl)vinyl)phenyl 4-methylbenzenesulfonate (W1A).** The compound was obtained using general method **2.1.3.** method and then crystallised from acetic acid, resulting in a white powder with a yield of 37 % (167 mg); mp 236-237 ^o^C; **^1^H NMR** (500 MHz, DMSO-*d_6_*) δ 12.43 (s, 1H), 8.13 (d, J = 8.5 Hz, 1H), 7.76 (d, J = 2.0 Hz, 1H), 7.70 (dd, J = 7.8, 1.7 Hz, 1H), 7.68 – 7.59 (m, 3H), 7.55 (dd, J = 8.5, 2.1 Hz, 1H), 7.53 – 7.49 (m, 1H), 7.44 (td, J = 7.6, 1.3 Hz, 1H), 7.32 (dd, J = 8.1, 1.3 Hz, 1H), 7.28 – 7.22 (m, 2H), 6.65 (d, J = 16.0 Hz, 1H), 2.15 (s, 3H);**^13^C NMR** (126 MHz, DMSO-*d_6_*) δ 161.57, 152.61, 150.45, 147.58, 146.44, 139.66, 131.77, 131.68, 131.45, 130.60, 129.14, 128.73, 128.59, 128.42, 127.74, 127.13, 126.82, 124.36, 123.34, 120.35, 21.45.; **HR-MS**: calculated for C_23_H_17_ClN_2_O_4_S [M+H]^+^: 453.06703 *m/z*, found 453.0691 *m/z*.

**(E)-3-(2-(7-chloro-4-oxo-3,4-dihydroquinazolin-2-yl)vinyl)phenyl 4-methylbenzenesulfonate (W1B).** The compound was obtained using general method **2.1.3.** and then crystallised from acetic acid, resulting in a white powder with a yield of 22 % (99 mg); mp 259 ^o^C; **^1^H NMR** (500 MHz, DMSO-*d_6_*) δ 12.46 (s, 1H), 8.10 (d, J = 8.5 Hz, 1H), 7.88 (d, J = 16.2 Hz, 1H), 7.82 – 7.76 (m, 2H), 7.71 (d, J = 2.0 Hz, 1H), 7.61 (dt, J = 7.9, 1.3 Hz, 1H), 7.55 – 7.49 (m, 2H), 7.51 – 7.43 (m, 2H), 7.36 (t, J = 2.0 Hz, 1H), 7.04 (ddd, J = 8.2, 2.4, 0.9 Hz, 1H), 6.95 (d, J = 16.2 Hz, 1H), 2.44 (s, 3H); **^13^C NMR** (126 MHz, DMSO-*d_6_*) δ 161.58, 152.97, 150.48, 149.98, 146.46, 139.59, 137.85, 137.37, 131.71, 131.22, 130.77, 128.77, 128.45, 127.16, 127.10, 126.67, 123.60, 123.06, 121.35, 120.49, 21.67.; **HR-MS**: calculated for C_23_H_17_ClN_2_O_4_S [M+H]^+^: 453.06703 *m/z*, found 453.0677*m/z*.

**(E)-4-(2-(7-chloro-4-oxo-3,4-dihydroquinazolin-2-yl)vinyl)phenyl 4-methylbenzenesulfonate (W1C).** The compound was obtained using general method **2.1.3.** and then crystallised from acetic acid, resulting in a white powder with a yield of 42 % (212 mg); mp 277-278 ^o^C; **^1^H NMR** (500 MHz, DMSO-*d_6_*) δ 12.48 (s, 1H), 8.10 (d, J = 8.5 Hz, 1H), 7.91 (d, J = 16.2 Hz, 1H), 7.80 – 7.74 (m, 2H), 7.71 (d, J = 2.0 Hz, 1H), 7.70 – 7.64 (m, 2H), 7.55 – 7.46 (m, 3H), 7.16 – 7.09 (m, 2H), 6.96 (d, J = 16.2 Hz, 1H), 2.44 (s, 3H); **^13^C NMR** (126 MHz, DMSO-*d_6_*) δ 161.60, 153.09, 150.53, 150.24, 146.45, 139.59, 137.96, 134.49, 131.77, 130.79, 129.82, 128.73, 128.43, 127.02, 126.65, 123.22, 122.37, 120.43, 21.67.; **HR-MS**: calculated for C_23_H_17_ClN_2_O_4_S [M+H]^+^: 453.06703 *m/z*, found 453.0686 *m/z*.

**(E)-3-(2-(8-chloro-4-oxo-3,4-dihydroquinazolin-2-yl)vinyl)phenyl 4-methylbenzenesulfonate (W2B).** The compound was obtained using general method **2.1.3.** and then crystallised from acetic acid, resulting in a white powder with a yield of 14 % (63 mg); mp 232-233 ^o^C; **^1^H NMR** (500 MHz, DMSO-*d_6_*) δ 12.55 (s, 1H), 8.08 (dd, J = 7.9, 1.4 Hz, 1H), 7.98 (dd, J = 7.8, 1.5 Hz, 1H), 7.90 (d, J = 16.1 Hz, 1H), 7.82 – 7.76 (m, 2H), 7.63 (dt, J = 7.9, 1.3 Hz, 1H), 7.53 – 7.44 (m, 4H), 7.37 (t, J = 2.0 Hz, 1H), 7.05 (ddd, J = 8.2, 2.4, 0.9 Hz, 1H), 6.98 (d, J = 16.2 Hz, 1H), 2.44 (s, 3H); **^13^C NMR** (126 MHz, DMSO-*d_6_*) δ 161.70, 152.24, 149.98, 146.47, 145.79, 138.01, 137.35, 135.16, 131.71, 131.21, 131.19, 130.77, 128.79, 127.21, 127.16, 125.51, 123.63, 123.39, 123.29, 121.42, 21.67.; **HR-MS**: calculated for C_23_H_17_ClN_2_O_4_S [M+H]^+^: 453.06703 *m/z*, found 453.0676 *m/z*.

# Representative spectra

## ^1^H NMR and ^13^C NMR spectra

**Fig. S1.** ^1^H NMR and ^13^C NMR data for compound W1A.

**Fig. S2.** ^1^H NMR and ^13^C NMR data for compound W1B.

**Fig. S3.** ^1^H NMR and ^13^C NMR data for compound W1C.

**Fig. S4.** ^1^H NMR and ^13^C NMR data for compound W2B.

- 1. HRMS spectra
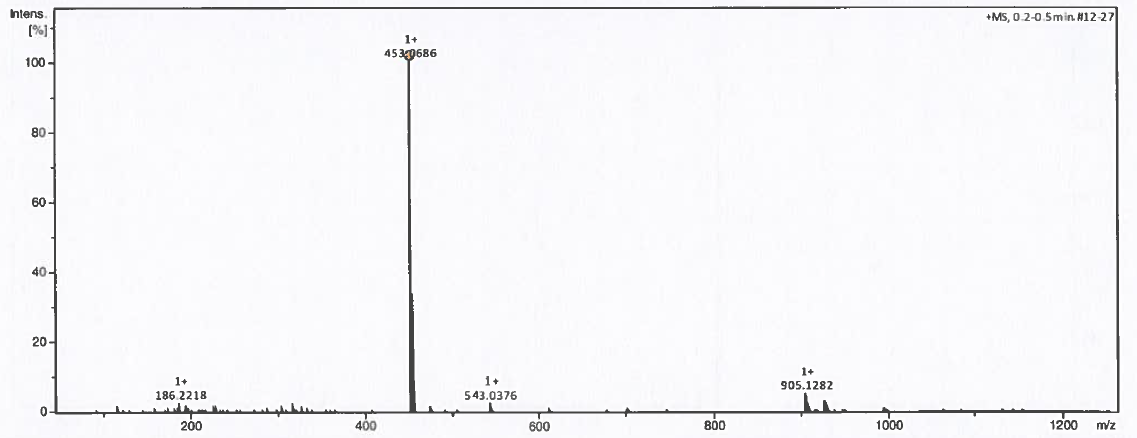


**Fig. S5.** HRMS data for compound W1A.


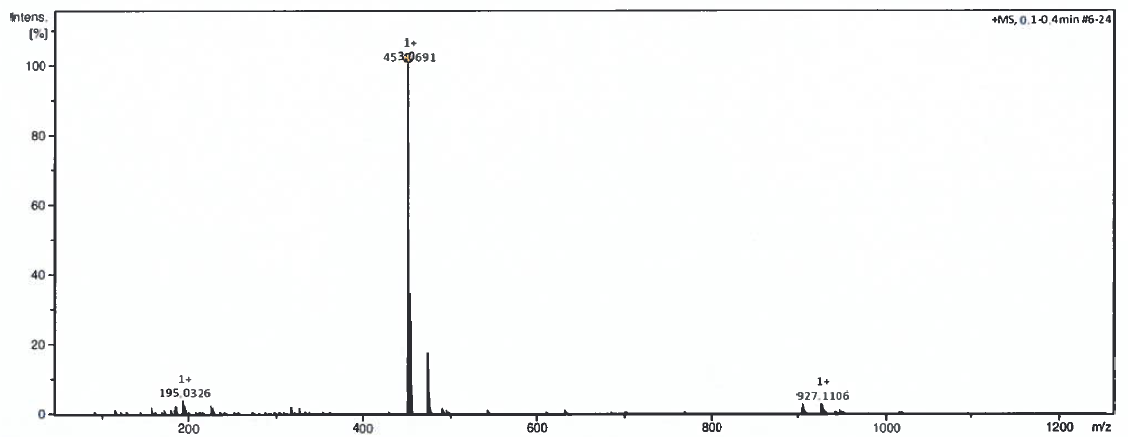


**Fig. S6.** HRMS data for compound W1B.


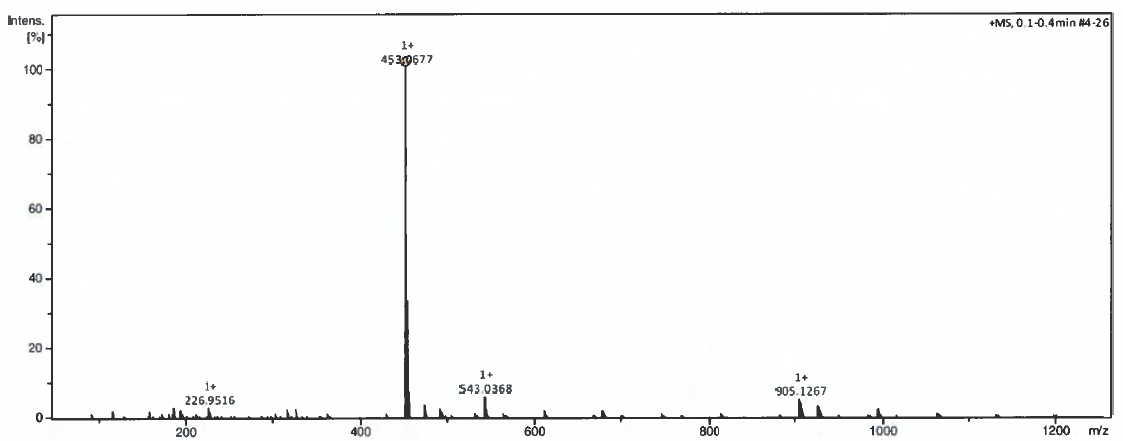


**Fig. S7.** HRMS data for compound W1C.


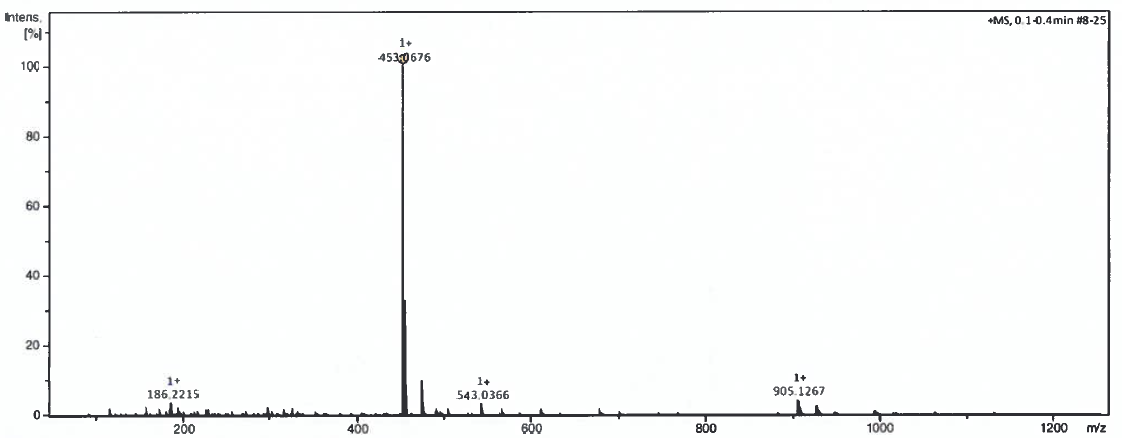


**Fig. S8.** HRMS data for compound W2B.

# Antiproliferative activity against epithelial cancer cells

**Table S1.** Antiproliferative activity of the tested compounds against MCF-7 and PC-9 cells.

| compound | **Antiproliferative activity - IC_50_ [µM]** | |
| --- | --- | --- |
|  | **MCF-7** | **PC-9** |
| **W1A** | >25 | >25 |
| **W1B** | 14.32 ± 2.39 | 6.54 ± 1.31 |
| **W1C** | >25 | >25 |
| **W2B** | >25 | >25 |

# Non-receptor tyrosine kinase inhibition studies

**Table S2.** Inhibition profile of W1B against non-receptor tyrosine kinases.

| compound | **% inhibition of kinases** | | | | | | | |
| --- | --- | --- | --- | --- | --- | --- | --- | --- |
|  | **ABL** | **BRK** | **BTK** | **CSK** | **Fyn A** | **Lck** | **Lyn B** | **Src** |
| **W1B** | 17.52 | 18.81 | 0 | 0 | 12.62 | 0 | 12.52 | 11.68 |

# Molecular docking

**
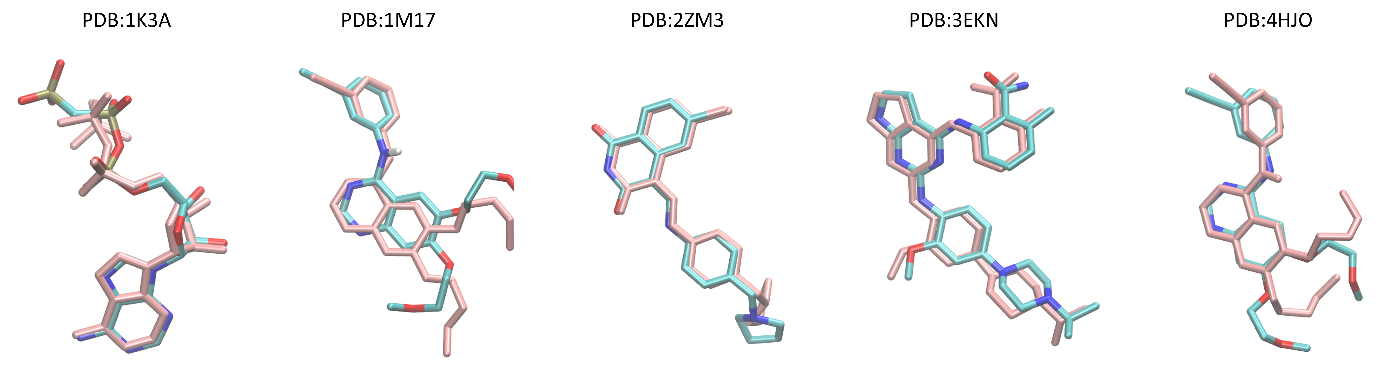
**

**Fig. S9.** Validation of the adopted docking protocol: comparison of crystallographic poses (colors correspond to atom types) and poses obtained by docking (pink colors). Protein structures from PDB: 1K3A (IGF1R), 1M17 (EGFR), 2ZM3 (IGF1R), 3EKN (InsR) and 4HJO (EGFR).

# Combinational Therapy

**Table S3.** Combination Index (CI) values shown at fraction affected (Fa) for W1B and Dacomitinib combination in LN229 and LN229 HG cell lines.

| **Cell Lines** | **Values** | **Drug Combination Ratio** |
| --- | --- | --- |
| LN229 | Fa = 0.9; CI Value = 0.94 | W1B + Dacomitinib [12:7] |
| LN229 HG | Fa = 0.9; CI Value = 0.87 | W1B + Dacomitinib [1.25:8.75] |


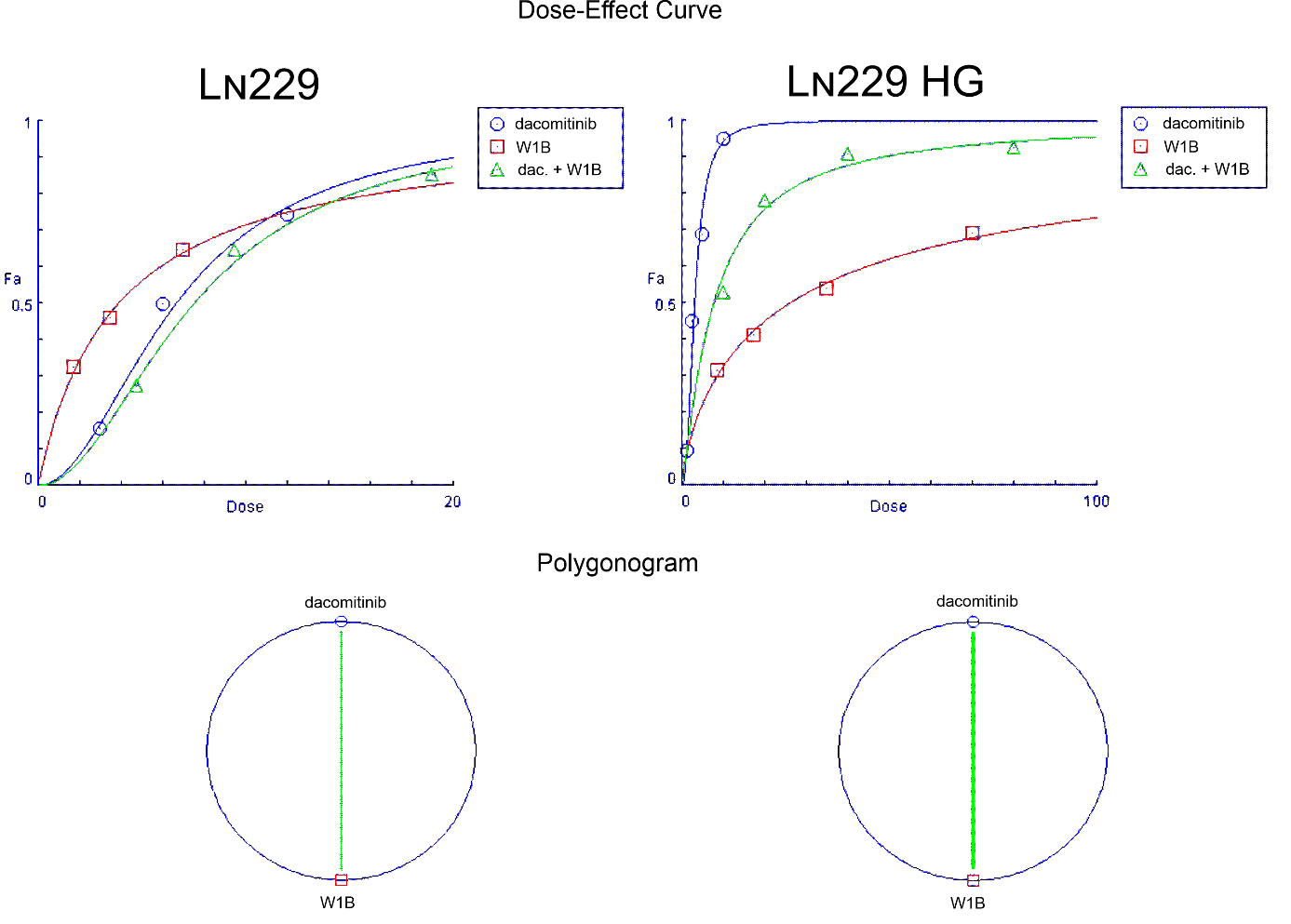


**Fig. S10.** Synergistic effect of W1B and dacomitinib combination therapy in LN229 and LN229 HG cells. Dose–response curves illustrating the effect of W1B, dacomitinib, and their combination and polygonogram graphically representing the drug interactions.

# Western Blot

## Densitometric analyses

**
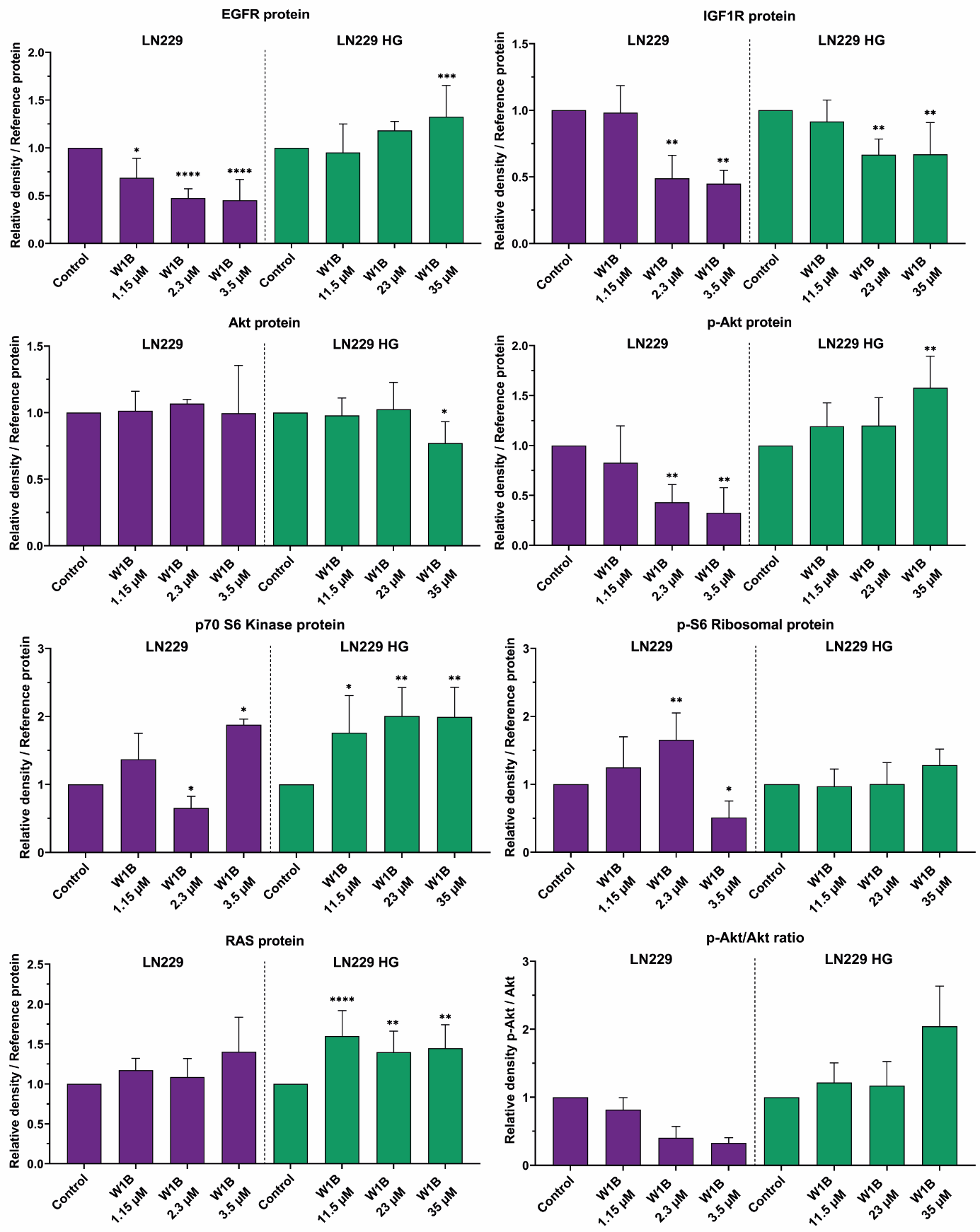
**

**Fig. S11.** Densitometric analyses of protein expression. Relative band intensities were normalized to the reference protein (GAPDH, vinculin, cyclophilin β or coffilin) and expressed as fold-change relative to untreated control cells. Data are presented as mean ± SD (n = 6) and were analysed using one-way ANOVA with Dunnett’s post-hoc test (*p < 0.1, **p < 0.01, ***p < 0.001, ****p < 0.0001).

## Raw data

Images of the gels prepared during this study. All gels are uncropped and unmodified. Proteins relevant to this study are marked in boxes along with reference proteins. Blue is LN229 cell line and red is LN229 cell line in High Glucose (HG). Purple line represent other experiment irrelevant to this study. R1 – Repeat 1, R2 – Repeat 2 etc..

- Targets: EGFR, IGF1R, Akt, p-Akt, p70 S6 Kinase, p-70 S6 Ribosomal Protein, RAS.
- Reference proteins: GAPDH, vinculin, cyclophilin β, coffilin.

Gel with a protein analyzed Later exposure time

**W1B 11.5 µM**

**Control LN229 HG**

**W1B 23 µM**

**W1B 35 µM**

**W1B 11.5 µM**

**Control LN229 HG**

**W1B 23 µM**

**W1B 35 µM**


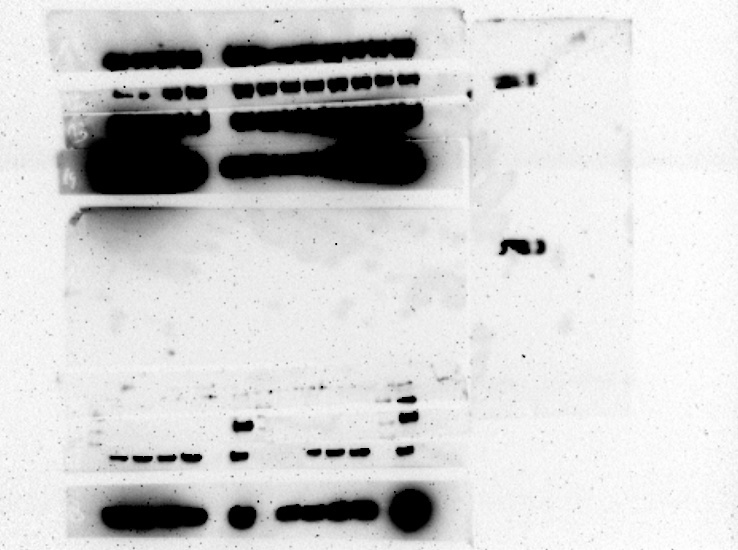

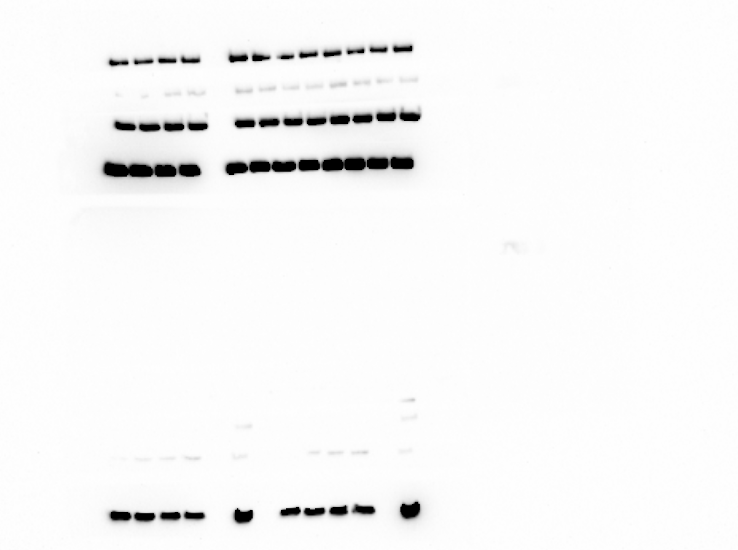


GAPDH

p-Akt R1

**W1B 11.5 µM**

**Control LN229 HG**

**W1B 23 µM**

**W1B 35 µM**

**W1B 11.5 µM**

**Control LN229 HG**

**W1B 23 µM**

**W1B 35 µM**

GAPDH

Akt R1-3

IGF1R R1-3

EGFR R1-3

Gel with a molecular weight marker in CCD camera Merged images below


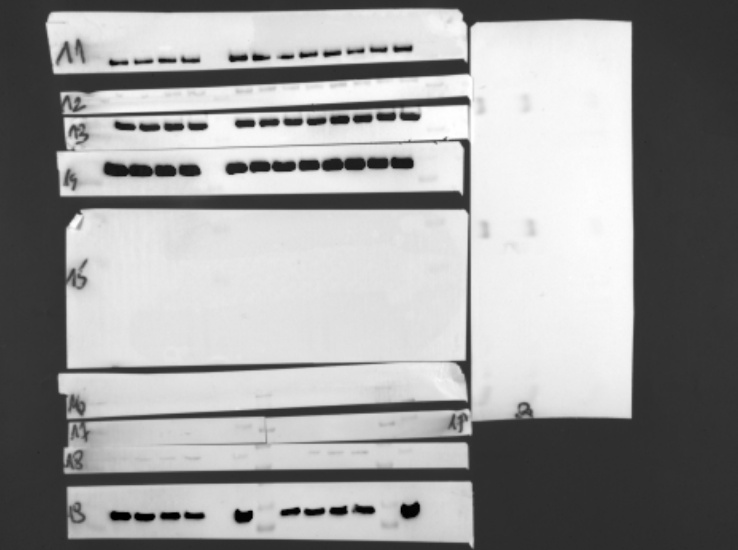
(colorimetric detection)


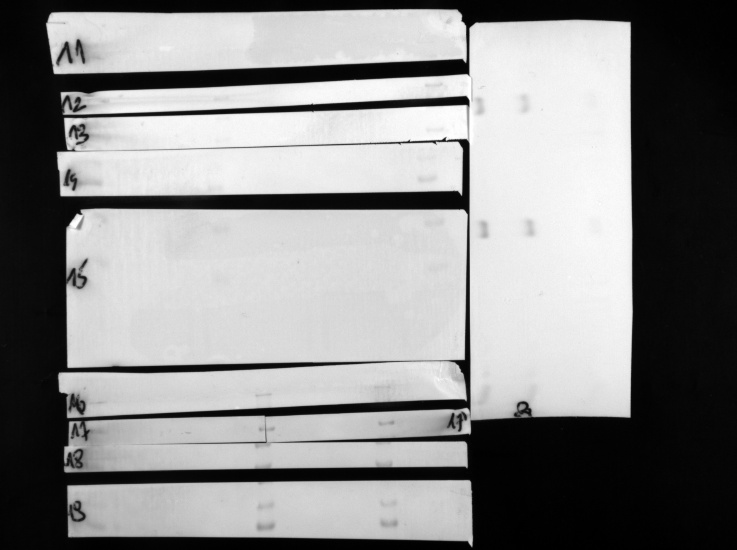


**260 kDa**

**140 kDa**

**100 kDa**

**70 kDa**

**25 kDa**

**40 kDa**

**50 kDa**

**35 kDa**

**15 kDa**

**260 kDa**

**140 kDa**

**100 kDa**

**70 kDa**

**25 kDa**

**40 kDa**

**50 kDa**

**35 kDa**

**15 kDa**

**10 kDa**

Gel with protein analyzed Later exposure time


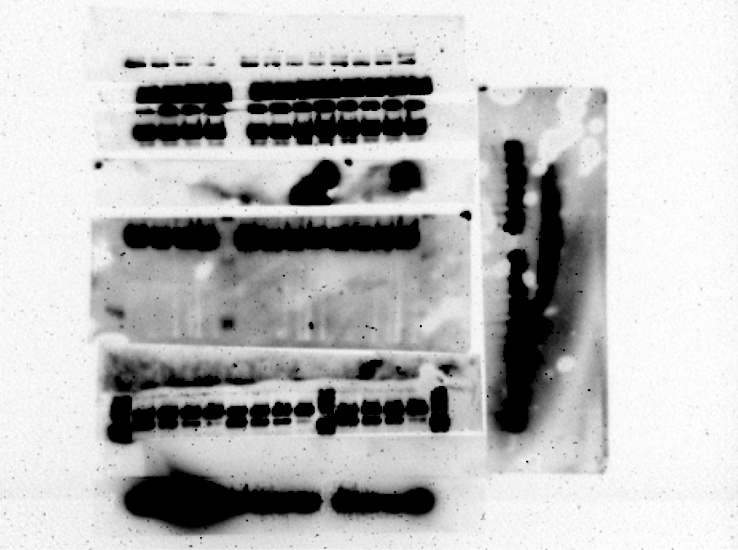

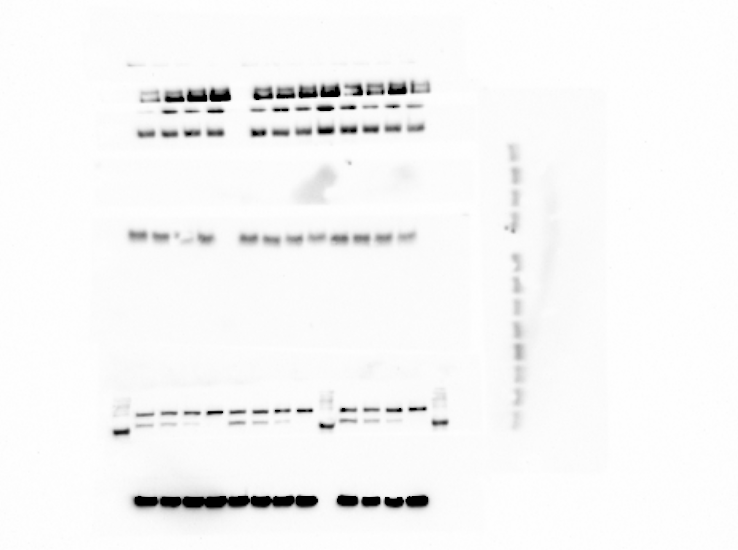


GAPDH

RAS R4-6

**W1B 11.5 µM**

**Control LN229 HG**

**W1B 23 µM**

**W1B 35 µM**

**W1B 11.5 µM**

**Control LN229 HG**

**W1B 23 µM**

**W1B 35 µM**

**W1B 11.5 µM**

**Control LN229 HG**

**W1B 23 µM**

**W1B 35 µM**

RAS R1-3

p70 S6 Kinase

R1-3

vinculin

**W1B 11.5 µM**

**Control LN229 HG**

**W1B 23 µM**

**W1B 35 µM**

**W1B 11.5 µM**

**Control LN229 HG**

**W1B 23 µM**

**W1B 35 µM**

**W1B 11.5 µM**

**Control LN229 HG**

**W1B 23 µM**

**W1B 35 µM**

Gel with a molecular weight marker in CCD camera Merged images below

(colorimetric detection)


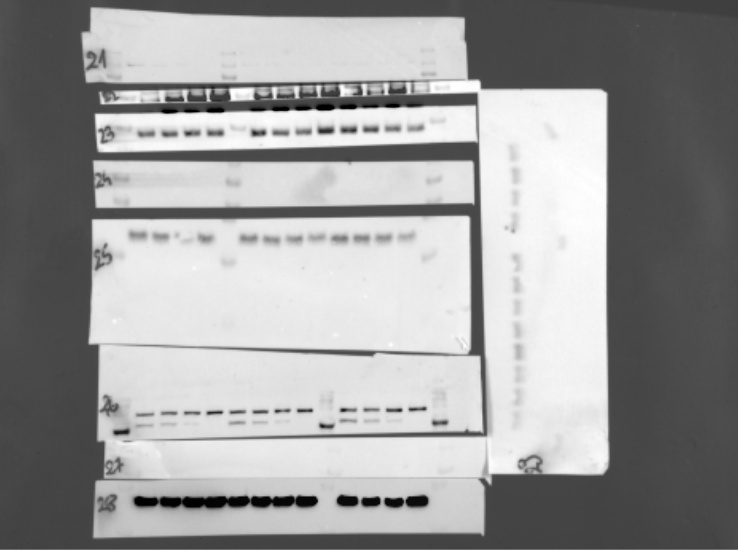

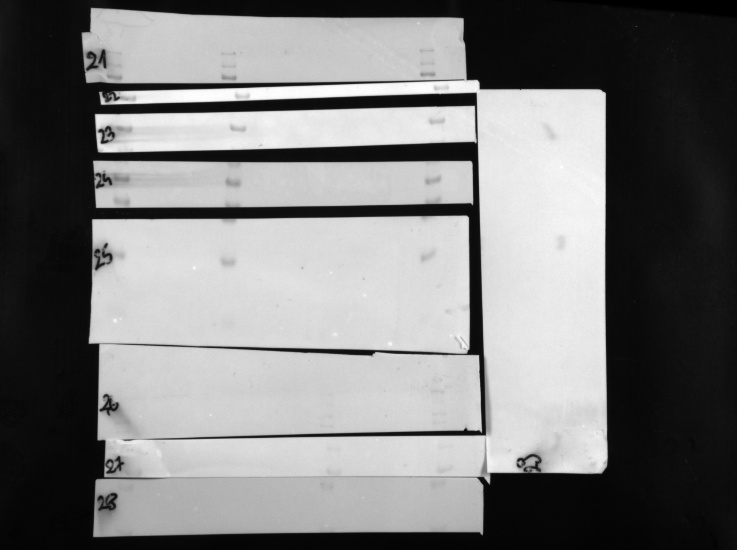


**260 kDa**

**160 kDa**

**110 kDa**

**80 kDa**

**30 kDa**

**50 kDa**

**60 kDa**

**40 kDa**

**15 kDa**

**260 kDa**

**140 kDa**

**100 kDa**

**70 kDa**

**25 kDa**

**40 kDa**

**50 kDa**

**35 kDa**

**15 kDa**

Gel with protein analyzed Later exposure time

EGFR R4-5

**W1B 11.5 µM**

**Control LN229 HG**

**W1B 23 µM**

**W1B 35 µM**


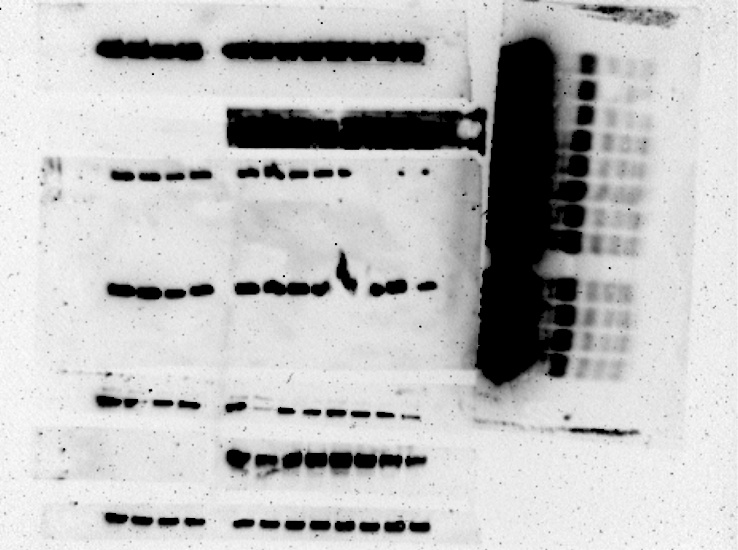

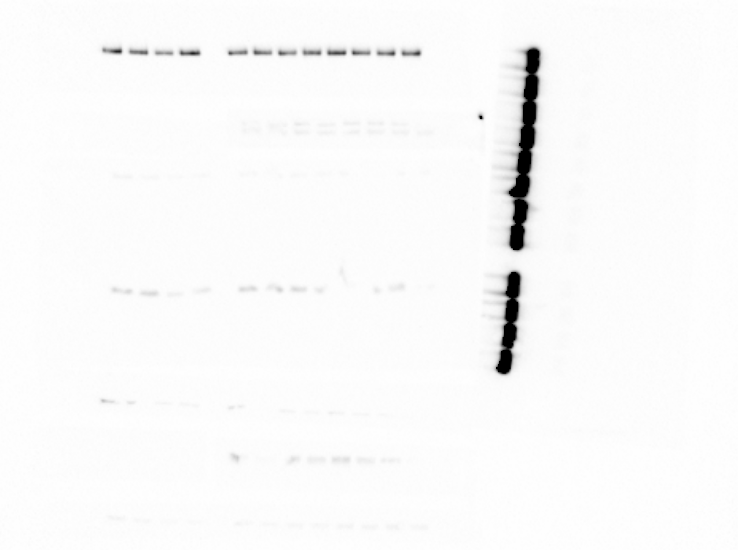


cyclophilin β

vinculin

p-70 S6 Ribosomal Protein

R1-3

**W1B 11.5 µM**

**Control LN229 HG**

**W1B 23 µM**

**W1B 35 µM**

**W1B 11.5 µM**

**Control LN229 HG**

**W1B 23 µM**

**W1B 35 µM**

**W1B 11.5 µM**

**Control LN229 HG**

**W1B 23 µM**

**W1B 35 µM**

**W1B 11.5 µM**

**Control LN229 HG**

**W1B 23 µM**

**W1B 35 µM**

Gel with a molecular weight marker in CCD camera Merged images below

(colorimetric detection)


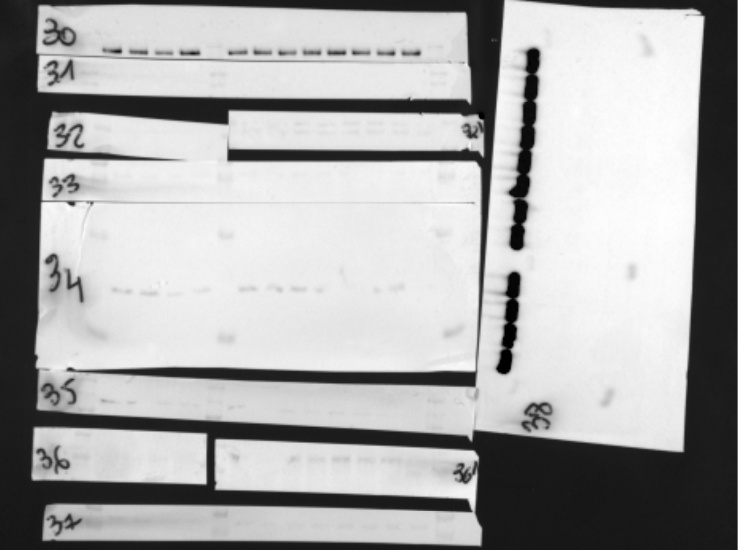

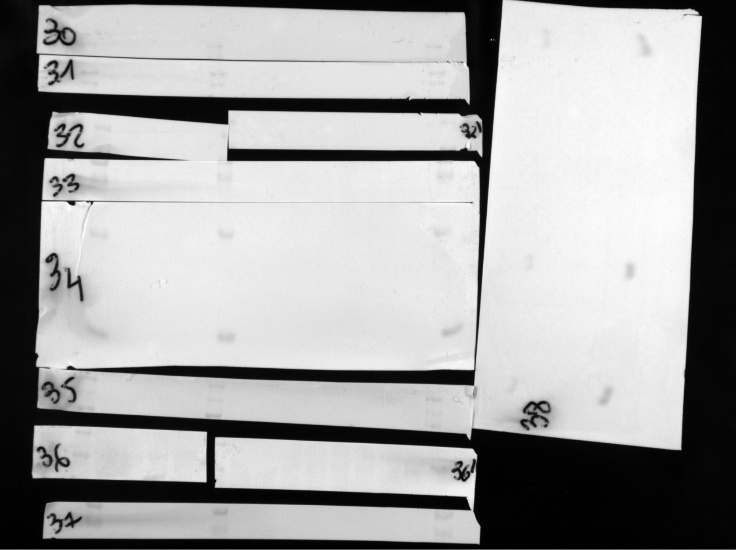


**260 kDa**

**160 kDa**

**110 kDa**

**80 kDa**

**30 kDa**

**50 kDa**

**60 kDa**

**40 kDa**

**15 kDa**

**260 kDa**

**160 kDa**

**110 kDa**

**80 kDa**

**30 kDa**

**50 kDa**

**60 kDa**

**40 kDa**

**15 kDa**

Gel with protein analyzed Later exposure time

**W1B 11.5 µM**

**Control LN229 HG**

**W1B 23 µM**

**W1B 35 µM**


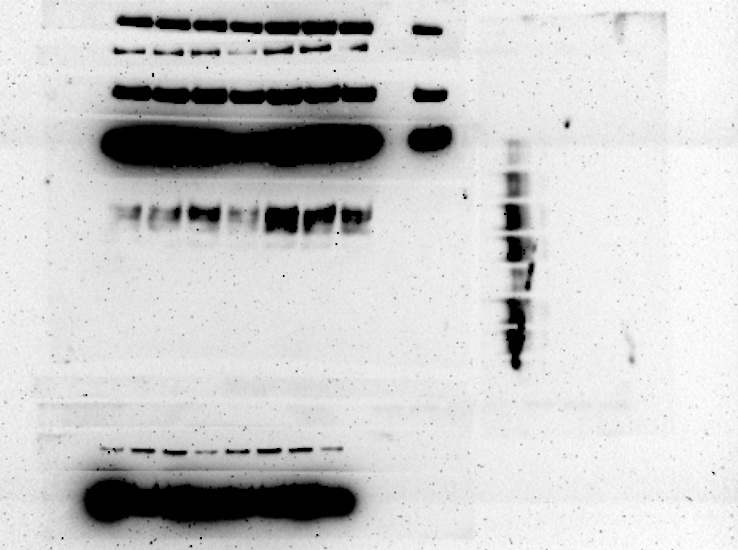

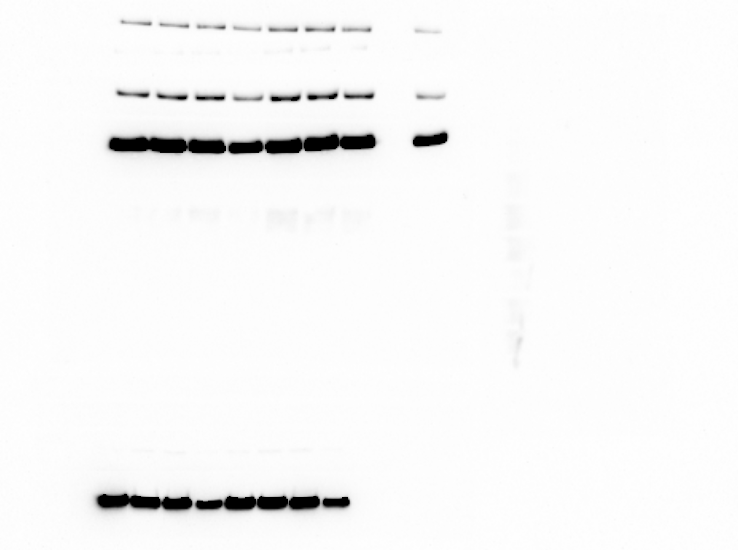


GAPDH

p-Akt R2-3

IGF1R R4

Akt R4-5

EGFR R6-7

GAPDH

**W1B 11.5 µM**

**Control LN229 HG**

**W1B 23 µM**

**W1B 35 µM**

Gel with a molecular weight marker in CCD camera Merged images below

(colorimetric detection)

**260 kDa**

**160 kDa**

**110 kDa**

**80 kDa**

**30 kDa**

**50 kDa**

**60 kDa**

**40 kDa**

**15 kDa**


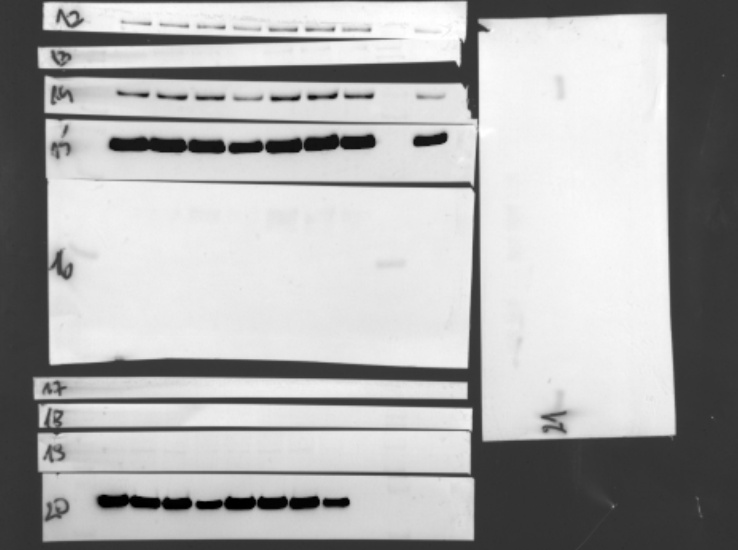

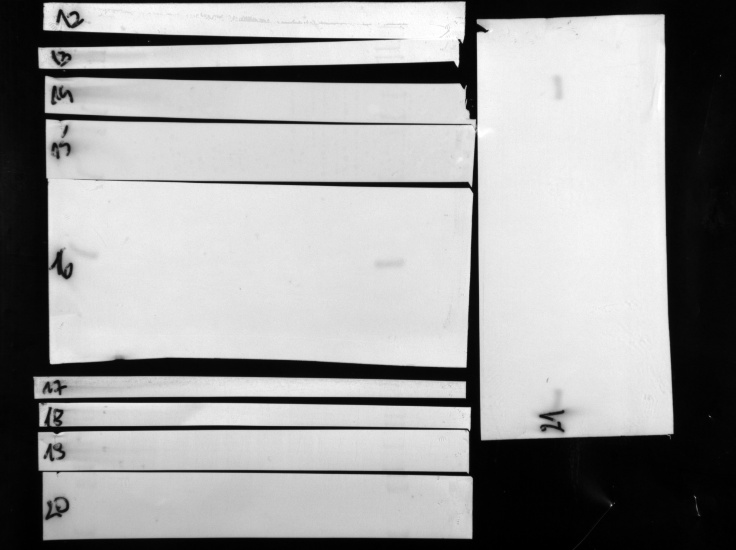


**260 kDa**

**160 kDa**

**110 kDa**

**80 kDa**

**30 kDa**

**50 kDa**

**60 kDa**

**40 kDa**

**15 kDa**

Gel with protein analyzed Later exposure time

p-70 S6 Ribosomal Protein

R4

**W1B 11.5 µM**

**Control LN229 HG**

**W1B 23 µM**

**W1B 35 µM**


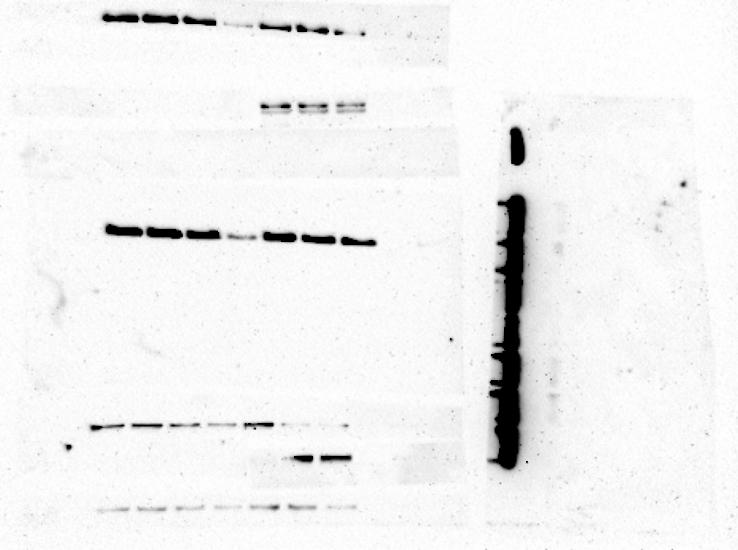

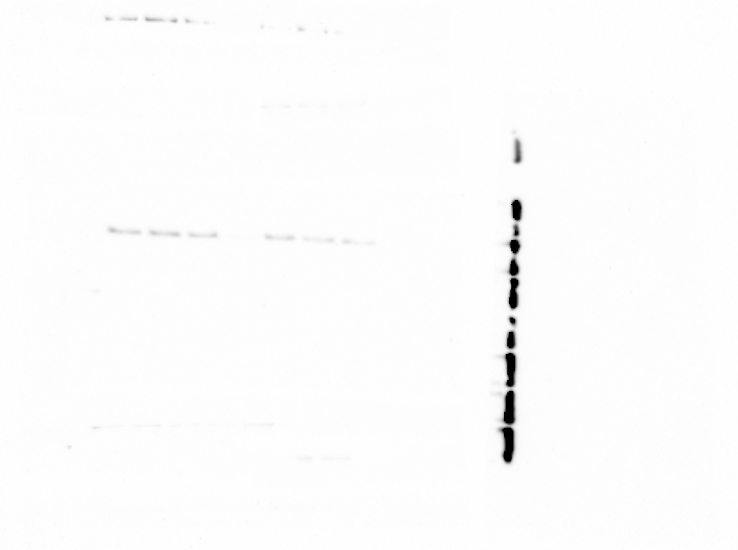


vinculin

cyclophilin β

EGFR R8

**W1B 11.5 µM**

**Control LN229 HG**

**W1B 23 µM**

**W1B 35 µM**

Gel with a molecular weight marker in CCD camera Merged images below


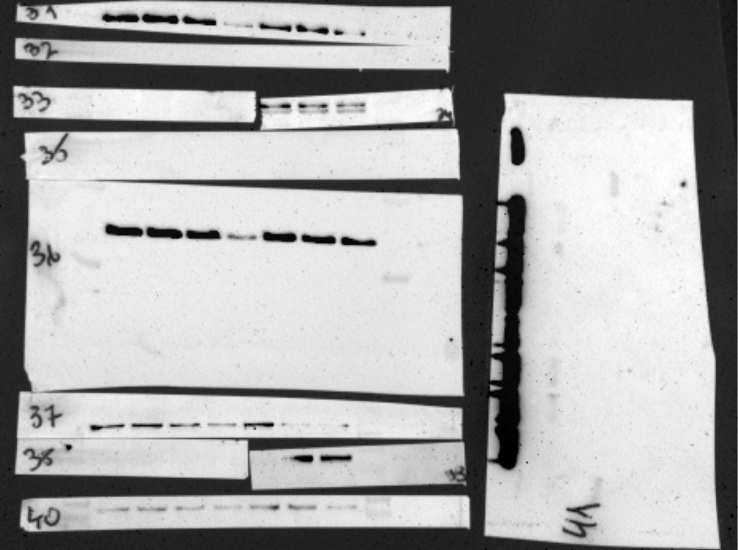
(colorimetric detection)


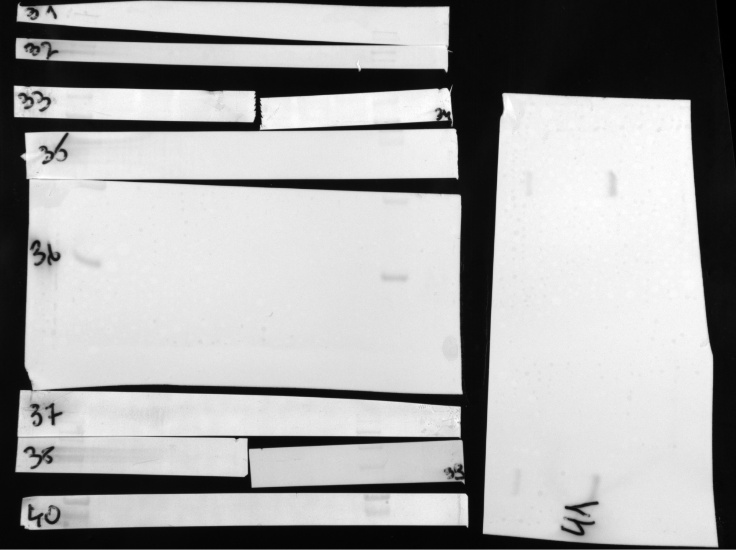


**260 kDa**

**160 kDa**

**110 kDa**

**80 kDa**

**30 kDa**

**50 kDa**

**60 kDa**

**40 kDa**

**15 kDa**

**260 kDa**

**160 kDa**

**110 kDa**

**80 kDa**

**30 kDa**

**50 kDa**

**60 kDa**

**40 kDa**

**15 kDa**

Gel with protein analyzed Later exposure time

**W1B 11.5 µM**

**Control LN229**

**W1B 23 µM**

**W1B 35 µM**


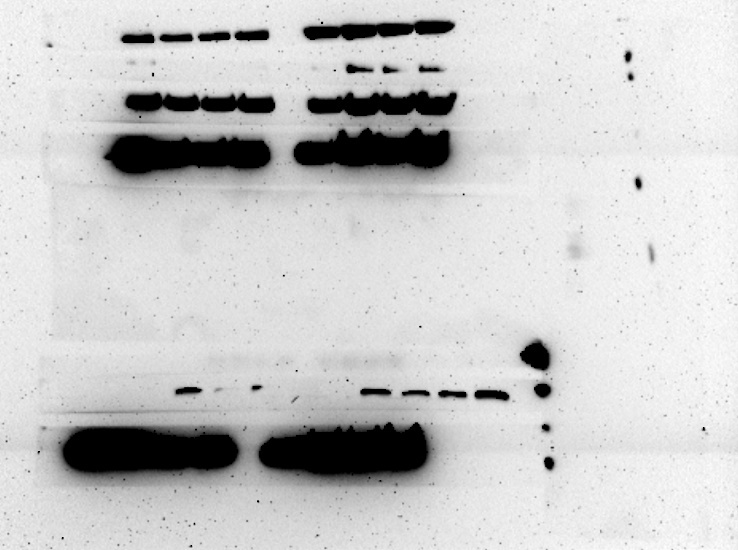

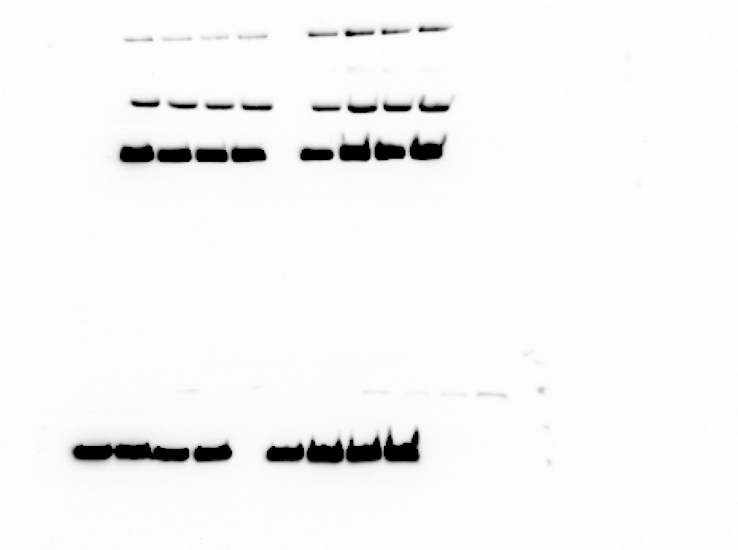


Akt R1

EGFR R1

IGF1R R5

Akt R6

EGFR R9

GAPDH

**W1B 11.5 µM**

**Control LN229 HG**

**W1B 23 µM**

**W1B 35 µM**

Gel with a molecular weight marker in CCD camera Merged images below

(colorimetric detection)

**260 kDa**

**160 kDa**

**110 kDa**

**80 kDa**

**30 kDa**

**50 kDa**

**60 kDa**

**40 kDa**

**15 kDa**


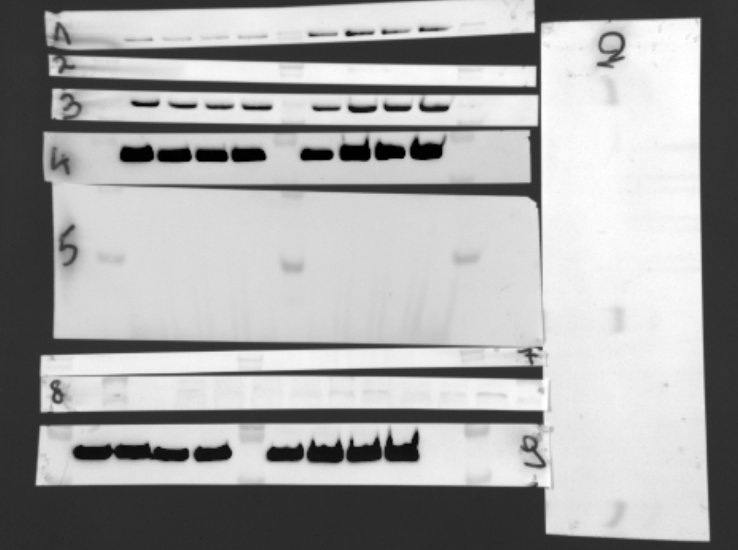

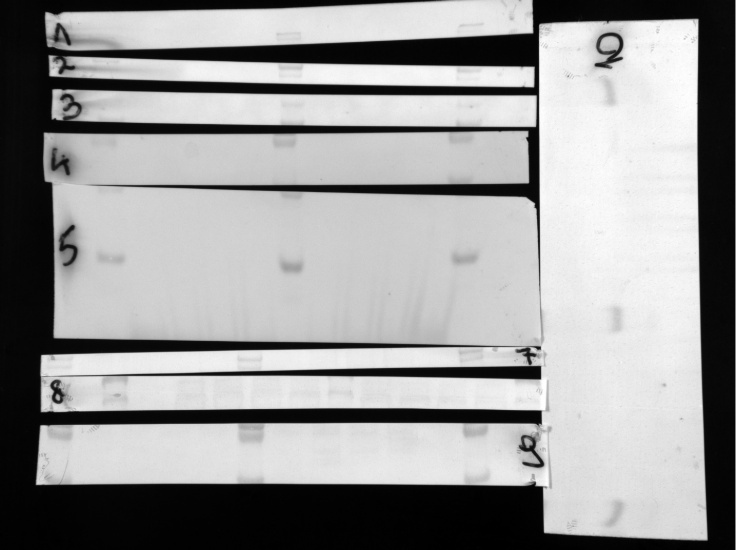


Gel with protein analyzed


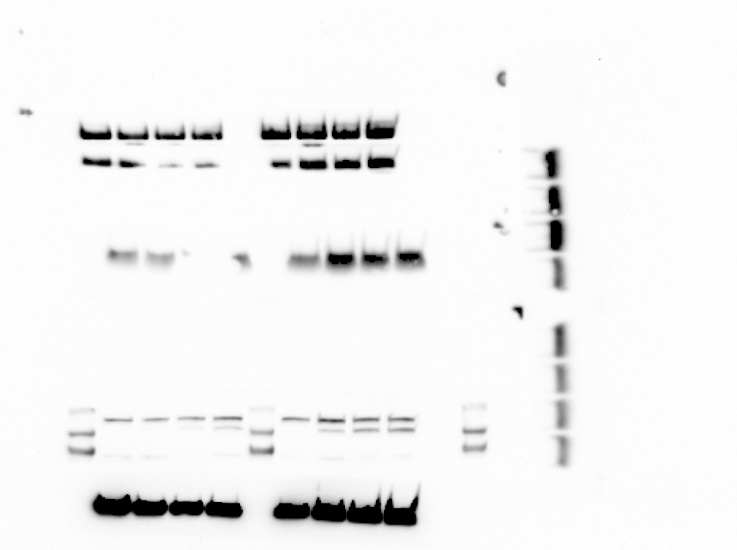


RAS R4

RAS R1

GAPDH

**W1B 11.5 µM**

**Control LN229 HG**

**W1B 23 µM**

**W1B 35 µM**

**W1B 11.5 µM**

**Control LN229**

**W1B 23 µM**

**W1B 35 µM**

Gel with a molecular weight marker in CCD camera Merged images below

(colorimetric detection)


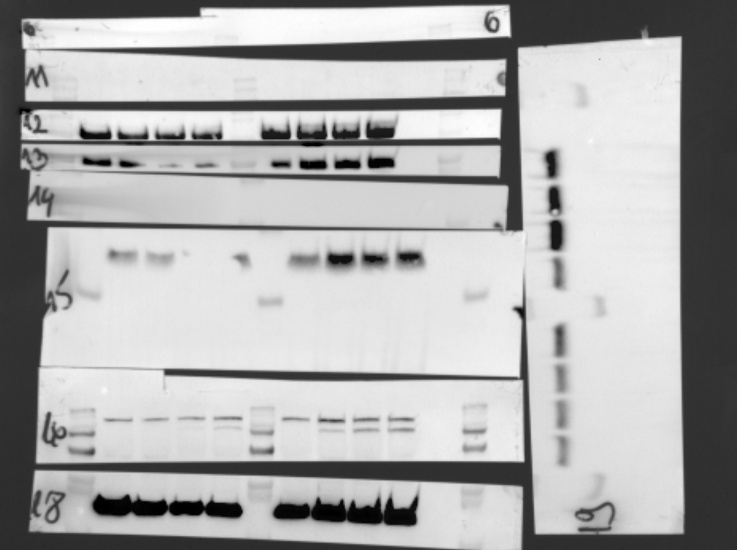

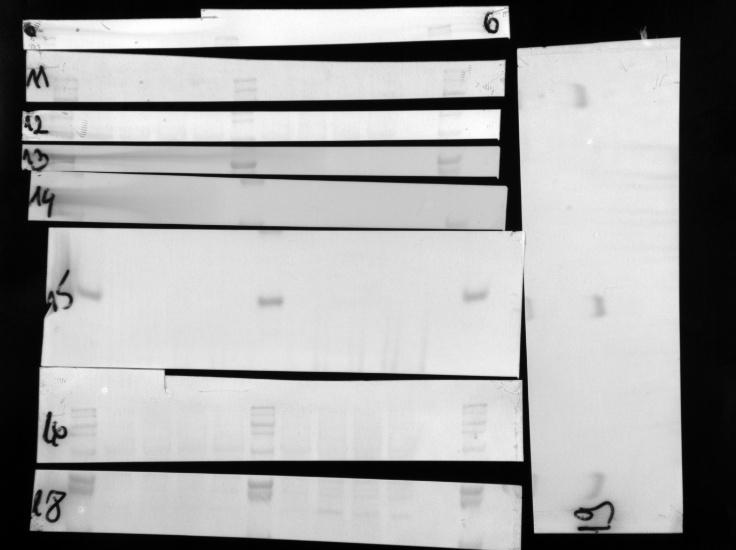


**260 kDa**

**160 kDa**

**110 kDa**

**80 kDa**

**30 kDa**

**50 kDa**

**60 kDa**

**40 kDa**

**15 kDa**

Gel with protein analyzed Later exposure time

**W1B 11.5 µM**

**Control LN229**

**W1B 23 µM**

**W1B 35 µM**


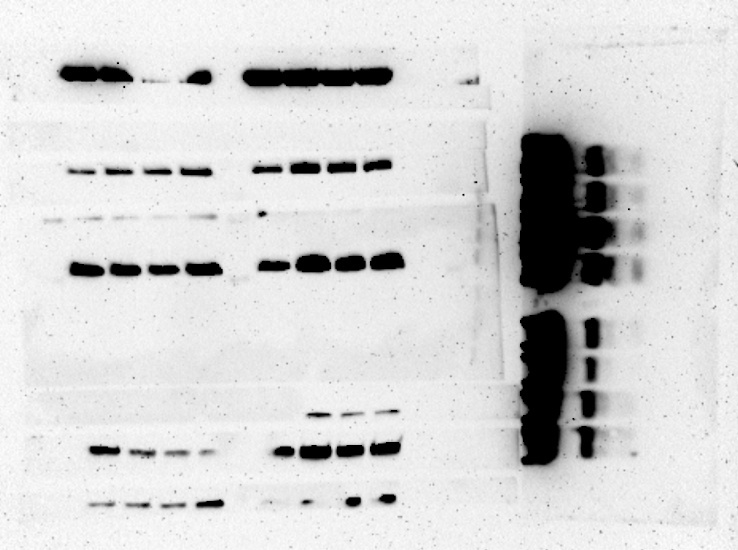

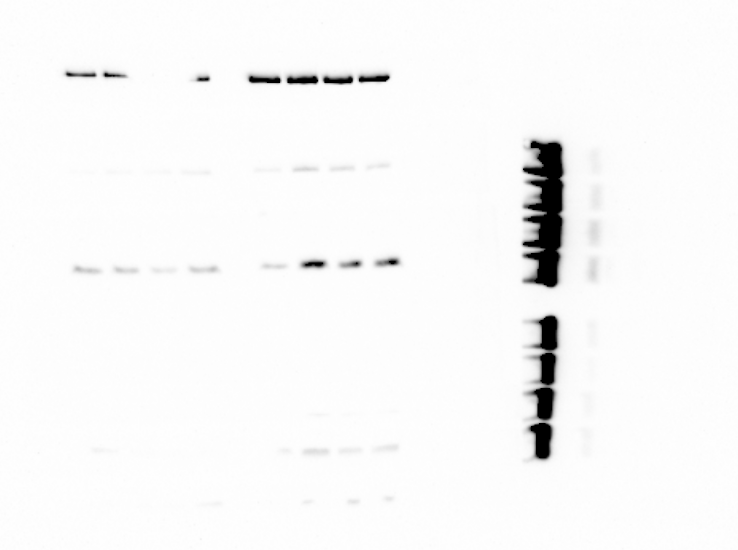


cyclophilin β

cyclophilin β

EGFR R10

EGFR R2

**W1B 11.5 µM**

**Control LN229 HG**

**W1B 23 µM**

**W1B 35 µM**

Gel with a molecular weight marker in CCD camera Merged images below

(colorimetric detection)


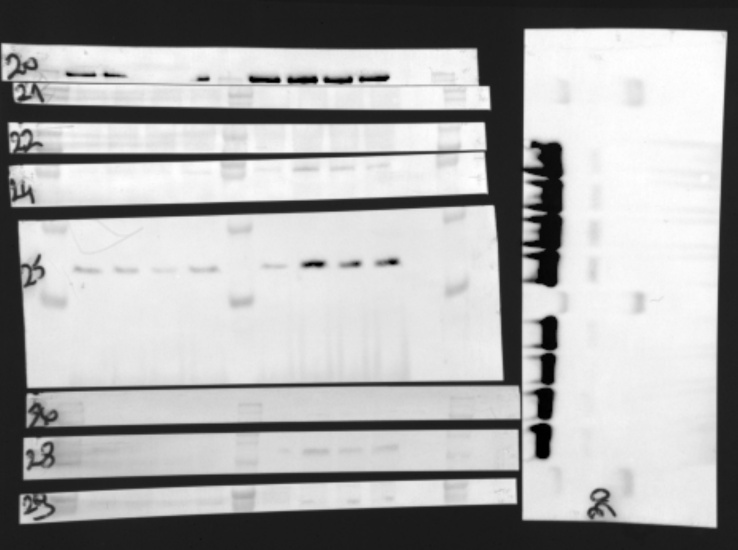

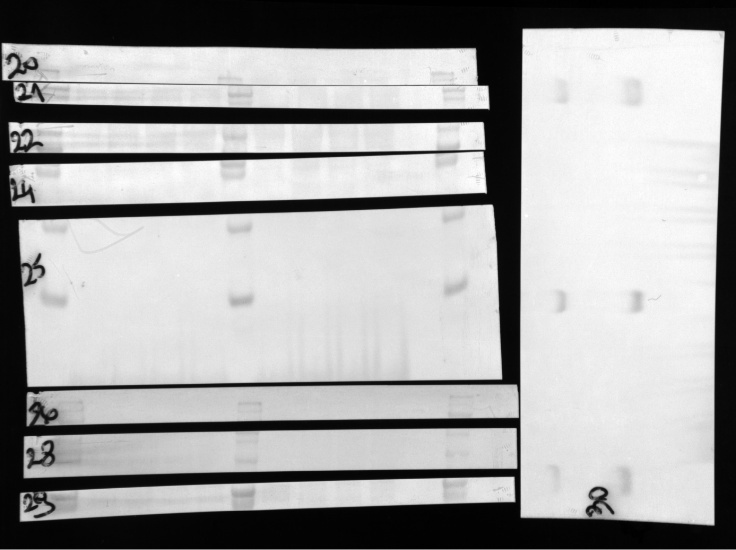


**260 kDa**

**160 kDa**

**110 kDa**

**80 kDa**

**30 kDa**

**50 kDa**

**60 kDa**

**40 kDa**

**15 kDa**


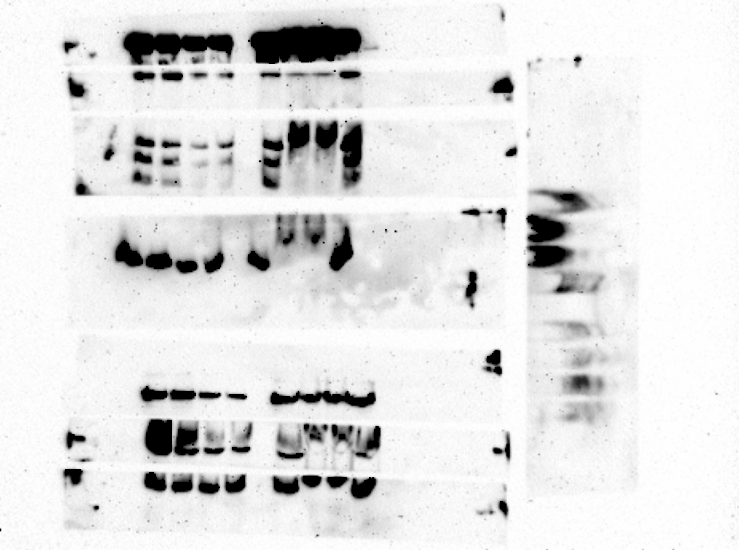

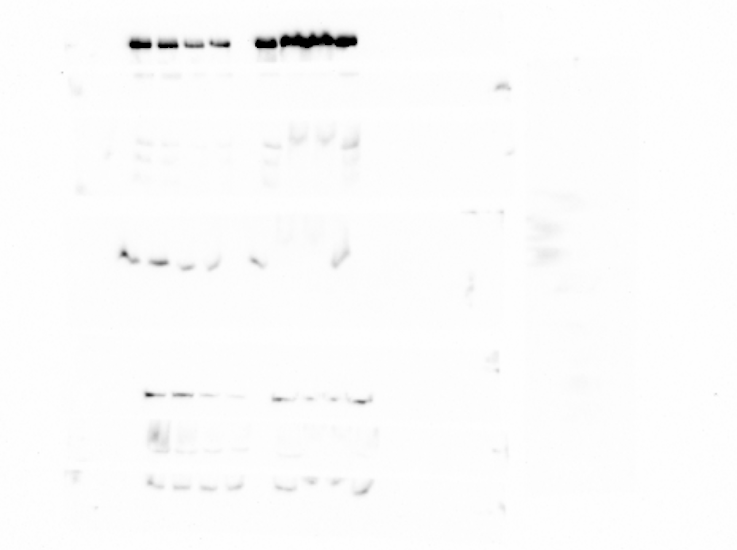
Gel with protein analyzed Later exposure time

vinculin

vinculin

p-Akt R4

p-Akt R1

IGF1R R1

IGF1R R6

EGFR R11

cyclophilin β

cyclophilin β

EGFR R3

**W1B 11.5 µM**

**Control LN229 HG**

**W1B 23 µM**

**W1B 35 µM**

**W1B 11.5 µM**

**Control LN229**

**W1B 23 µM**

**W1B 35 µM**

Gel with a molecular weight marker in CCD camera Merged images below

(colorimetric detection)


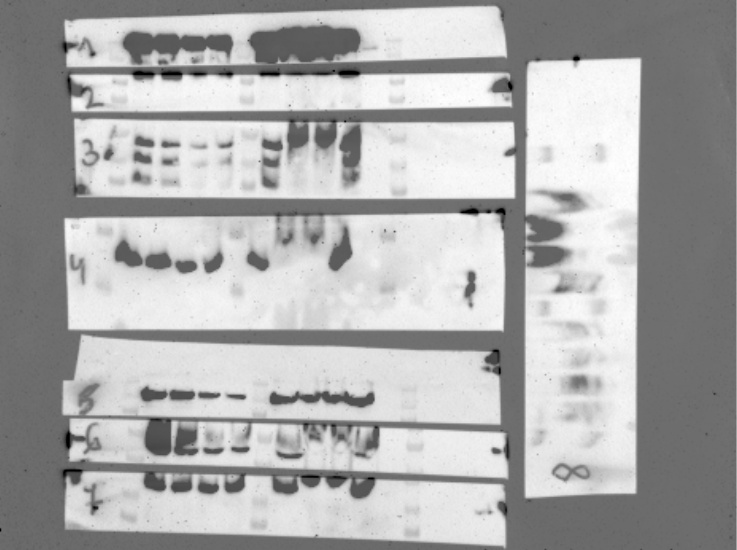

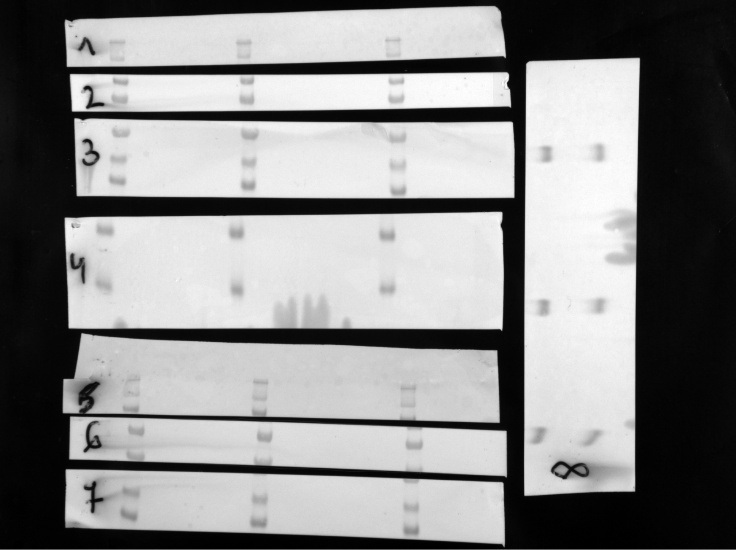


**260 kDa**

**140 kDa**

**100 kDa**

**70 kDa**

**25 kDa**

**40 kDa**

**50 kDa**

**35 kDa**

**15 kDa**

**260 kDa**

**140 kDa**

**100 kDa**

**70 kDa**

**25 kDa**

**40 kDa**

**50 kDa**

**35 kDa**

**15 kDa**

Gel with protein analyzed

**W1B 11.5 µM**

**Control LN229**

**W1B 23 µM**

**W1B 35 µM**


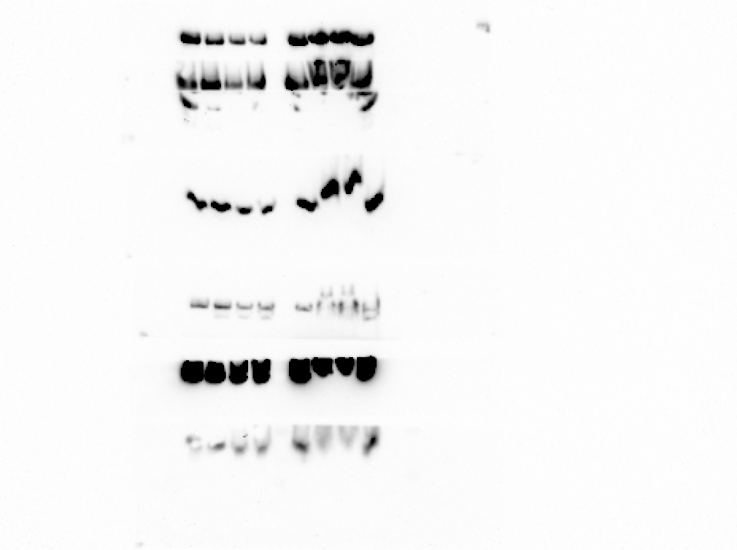


p70 S6 Kinase

R4

p70 S6 Kinase

R1

RAS R5

RAS R2

GAPDH

GAPDH

cyclophilin β

cyclophilin β

EGFR R12

EGFR R4

**W1B 11.5 µM**

**Control LN229 HG**

**W1B 23 µM**

**W1B 35 µM**

Gel with a molecular weight marker in CCD camera Merged images below

(colorimetric detection)


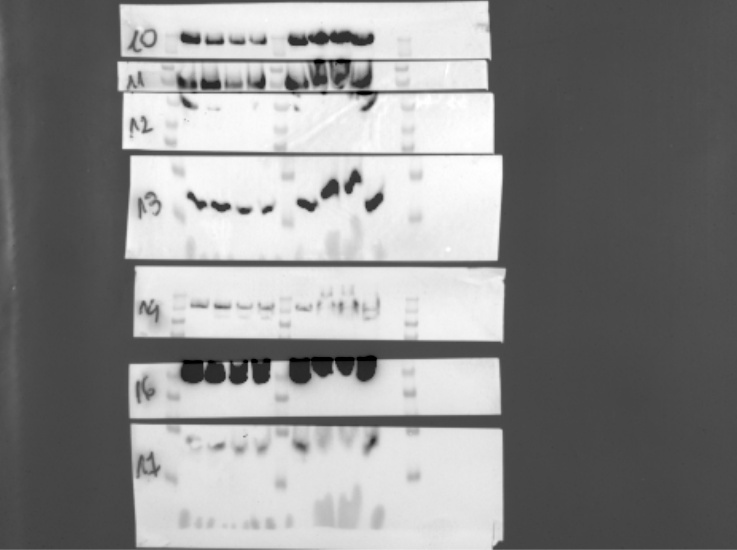

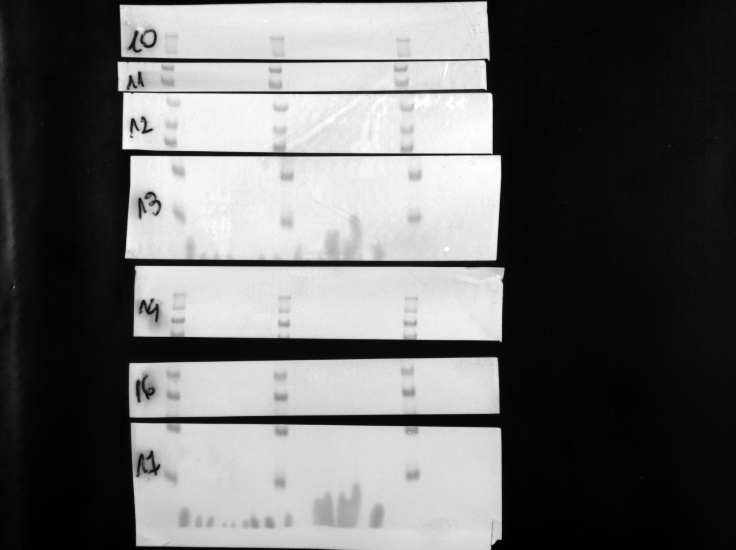


**260 kDa**

**140 kDa**

**100 kDa**

**70 kDa**

**25 kDa**

**40 kDa**

**35 kDa**

**15 kDa**

**260 kDa**

**140 kDa**

**100 kDa**

**70 kDa**

**25 kDa**

**40 kDa**

**50 kDa**

**35 kDa**

**15 kDa**

Gel with protein analyzed

EGFR R13

EGFR R5

**W1B 11.5 µM**

**Control LN229**

**W1B 23 µM**

**W1B 35 µM**


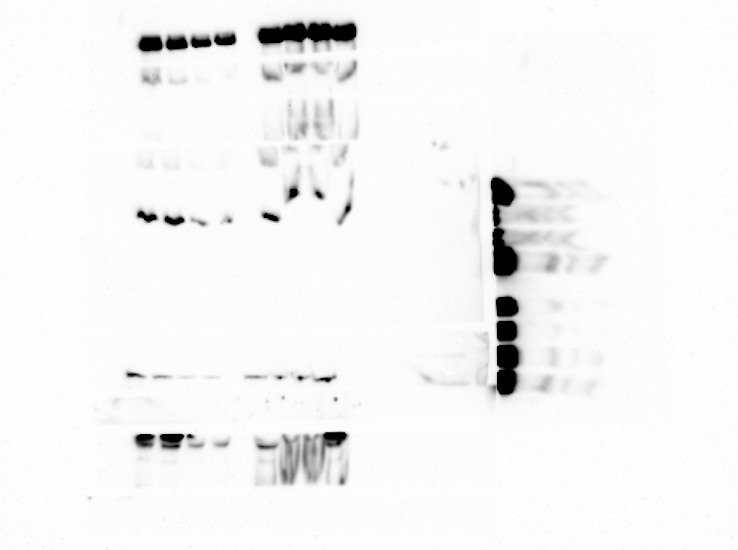


p-70 S6 Ribosomal

Protein R1

p-70 S6 Ribosomal

Protein R5

vinculin

vinculin

cyclophilin β

cyclophilin β

**W1B 11.5 µM**

**Control LN229 HG**

**W1B 23 µM**

**W1B 35 µM**

Gel with a molecular weight marker in CCD camera Merged images below

(colorimetric detection)


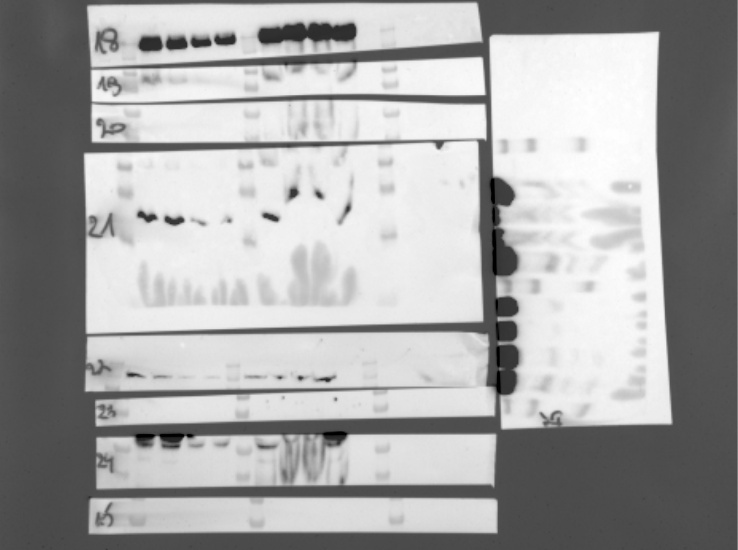

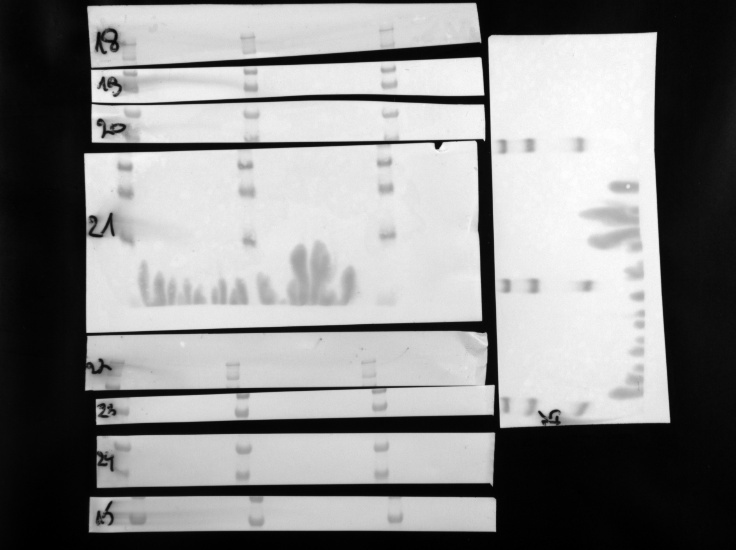


**260 kDa**

**140 kDa**

**100 kDa**

**70 kDa**

**25 kDa**

**40 kDa**

**50 kDa**

**35 kDa**

**15 kDa**

**260 kDa**

**140 kDa**

**100 kDa**

**70 kDa**

**25 kDa**

**40 kDa**

**50 kDa**

**35 kDa**

**15 kDa**

Gel with protein analyzed Later exposure time

**W1B 11.5 µM**

**Control LN229**

**W1B 23 µM**

**W1B 35 µM**


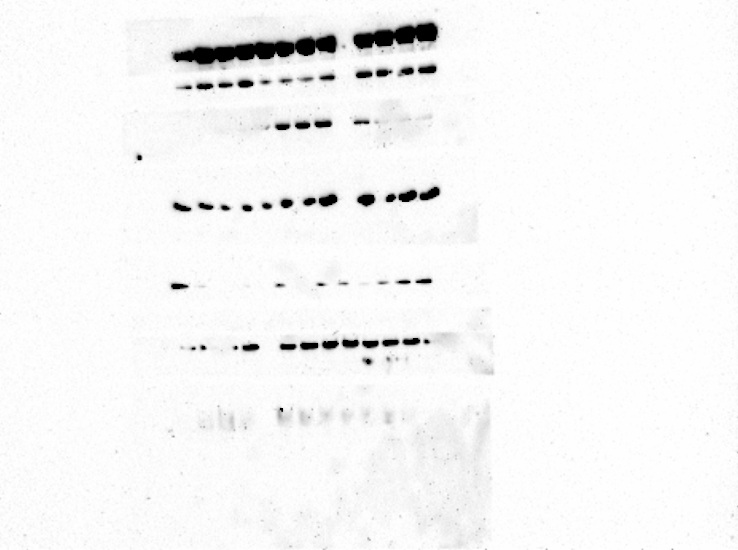

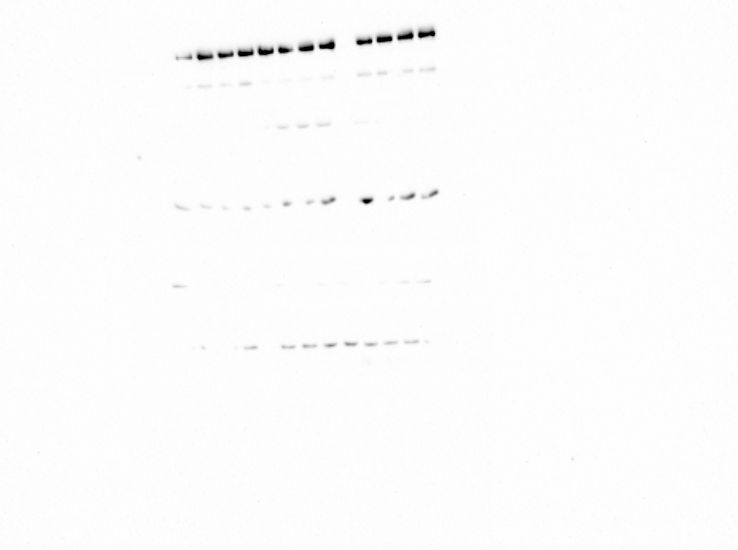


**W1B 11.5 µM**

**Control LN229 HG**

**W1B 23 µM**

**W1B 35 µM**

IGF1R R7

IGF1R R2-3

EGFR R14

cyclophilin β

cyclophilin β

EGFR R5-6

**W1B 11.5 µM**

**Control LN229**

**W1B 23 µM**

**W1B 35 µM**

Gel with a molecular weight marker in CCD camera Merged images below

(colorimetric detection)


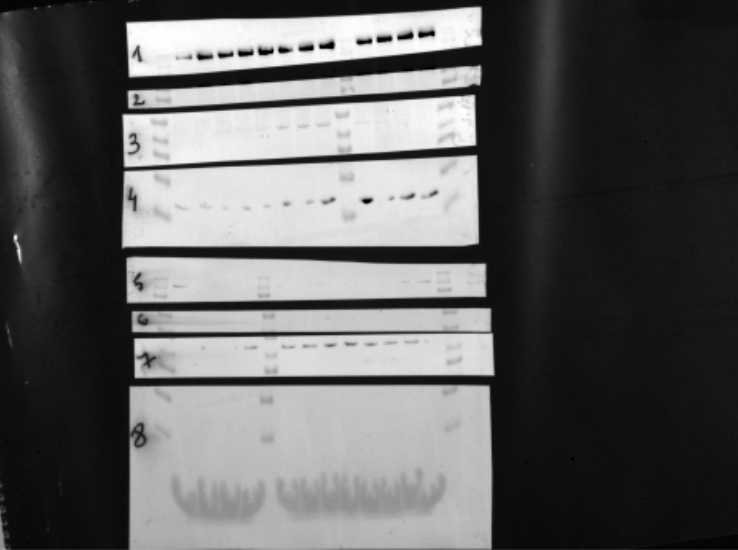

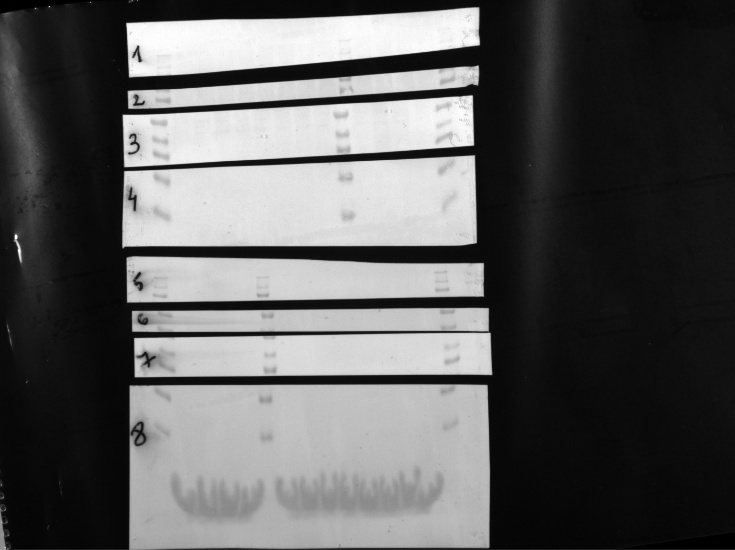


**260 kDa**

**140 kDa**

**100 kDa**

**70 kDa**

**25 kDa**

**40 kDa**

**50 kDa**

**35 kDa**

**15 kDa**

Gel with protein analyzed Later exposure time

**W1B 11.5 µM**

**Control LN229**

**W1B 23 µM**

**W1B 35 µM**

**W1B 11.5 µM**

**Control LN229**

**W1B 23 µM**

**W1B 35 µM**


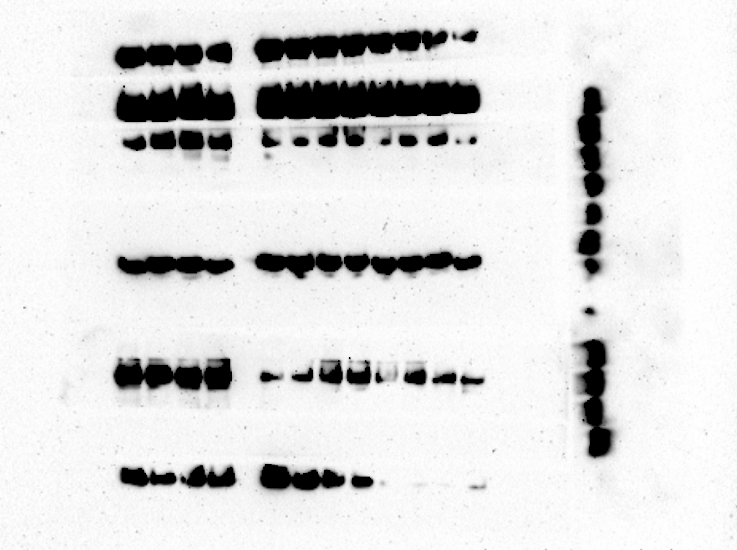

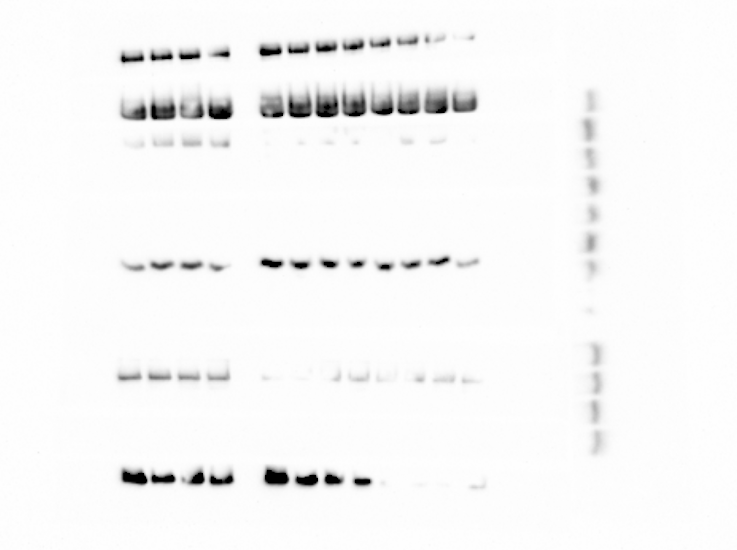


EGFR R14

GAPDH

GAPDH

RAS R6

RAS R3

p70 S6 Kinase

R5

cyclophilin β

cyclophilin β

p70 S6 Kinase

R2-3

EGFR R6-7

**W1B 11.5 µM**

**Control LN229 HG**

**W1B 23 µM**

**W1B 35 µM**

Gel with a molecular weight marker in CCD camera Merged images below

(colorimetric detection)


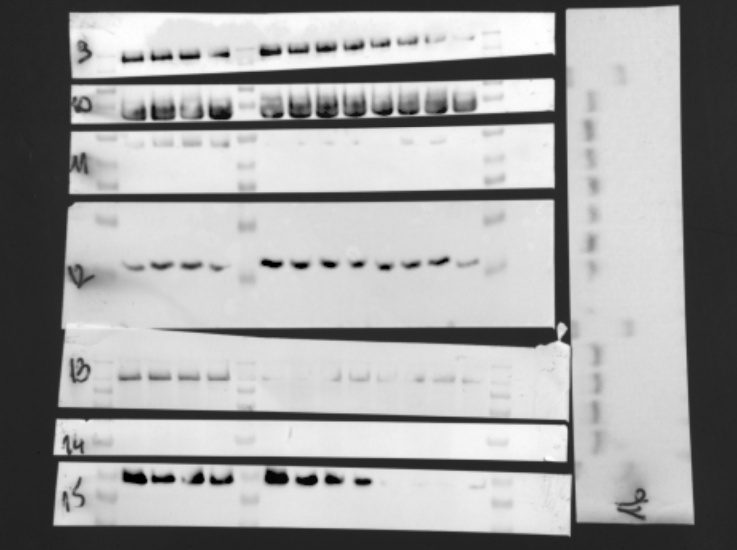

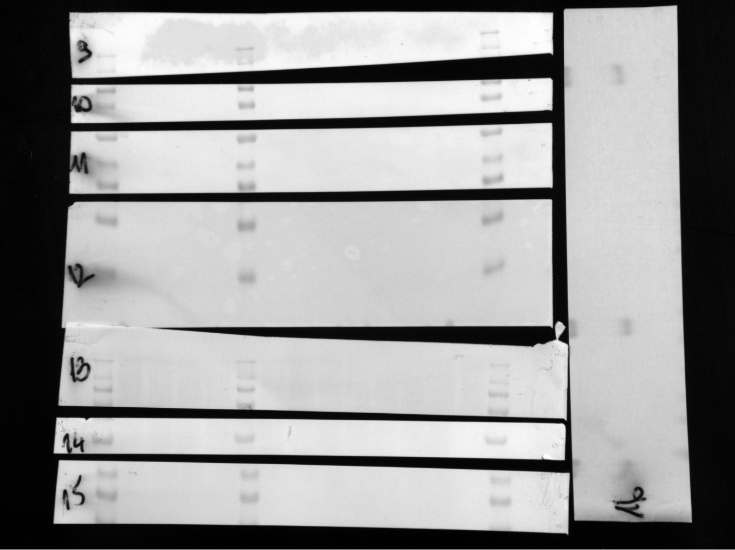


**260 kDa**

**140 kDa**

**100 kDa**

**70 kDa**

**25 kDa**

**40 kDa**

**50 kDa**

**35 kDa**

**15 kDa**

**260 kDa**

**140 kDa**

**100 kDa**

**70 kDa**

**25 kDa**

**40 kDa**

**50 kDa**

**35 kDa**

**15 kDa**

Gel with protein analyzed Later exposure time


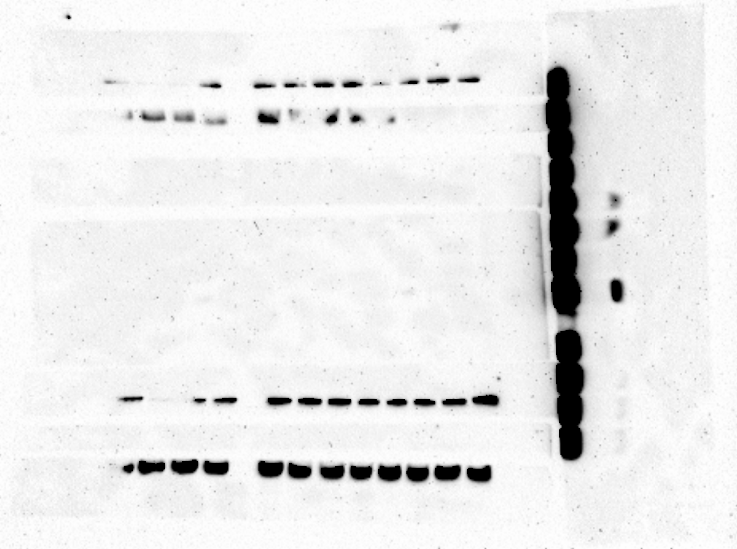

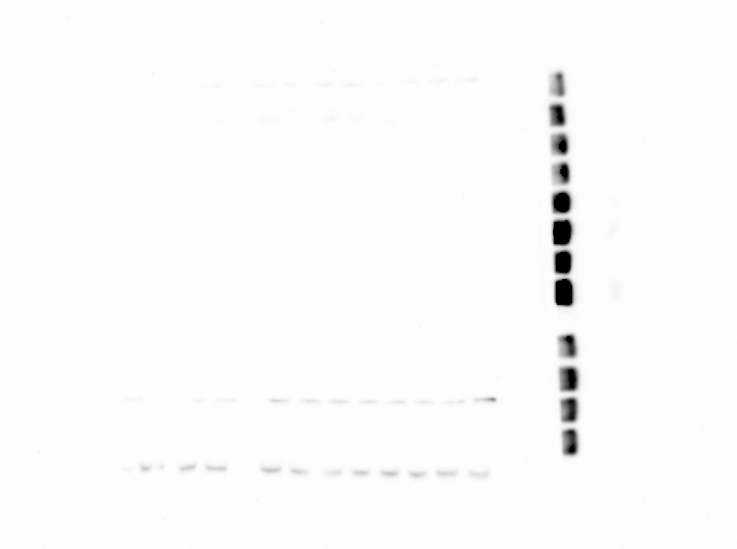


p-70 S6 Ribosomal

Protein R6

**W1B 11.5 µM**

**Control LN229 HG**

**W1B 23 µM**

**W1B 35 µM**

p-70 S6 Ribosomal

Protein R2-3

vinculin

vinculin

vinculin

**W1B 11.5 µM**

**Control LN229**

**W1B 23 µM**

**W1B 35 µM**

**W1B 11.5 µM**

**Control LN229**

**W1B 23 µM**

**W1B 35 µM**

Gel with a molecular weight marker in CCD camera Merged images below


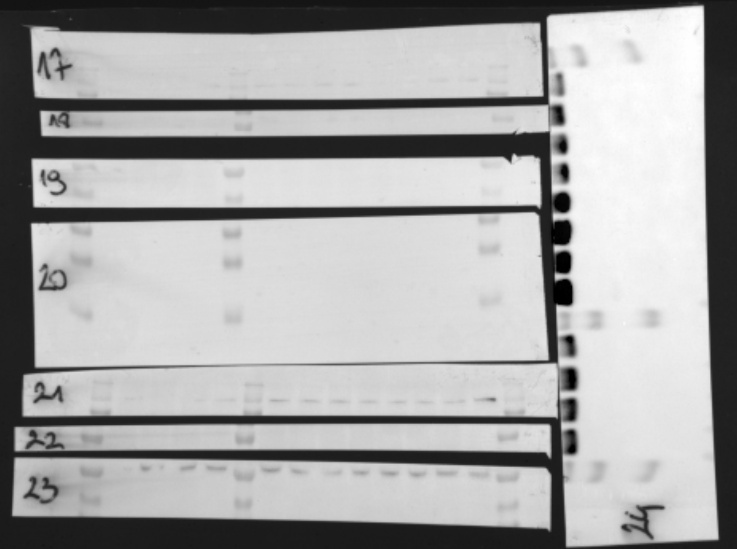
(colorimetric detection)


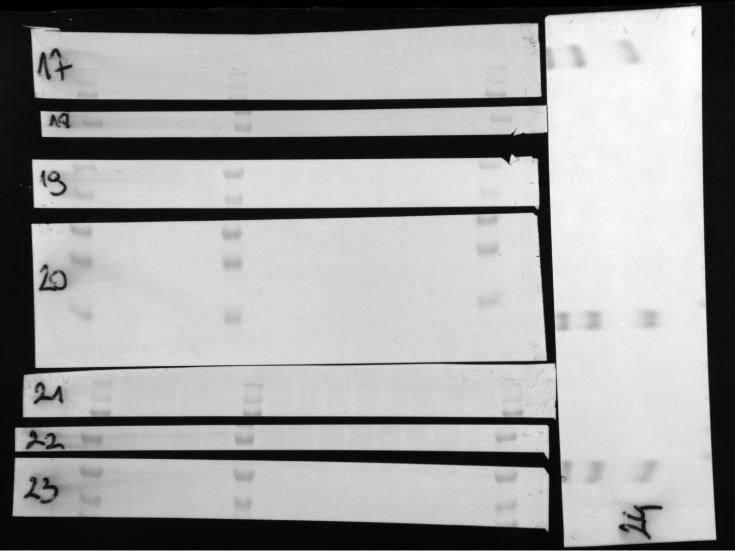


**260 kDa**

**140 kDa**

**100 kDa**

**70 kDa**

**25 kDa**

**40 kDa**

**50 kDa**

**35 kDa**

**15 kDa**

Gel with protein analyzed


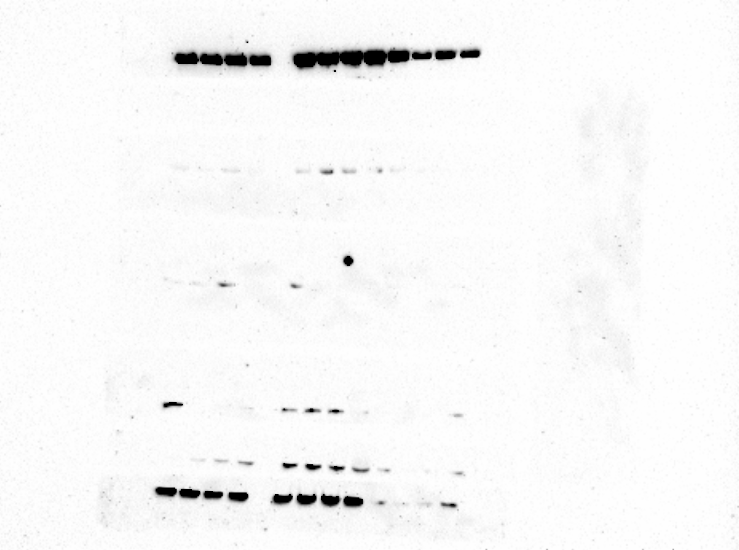


vinculin

p-Akt R5

**W1B 11.5 µM**

**Control LN229 HG**

**W1B 23 µM**

**W1B 35 µM**

Gel with a molecular weight marker in CCD camera Merged images below

(colorimetric detection)


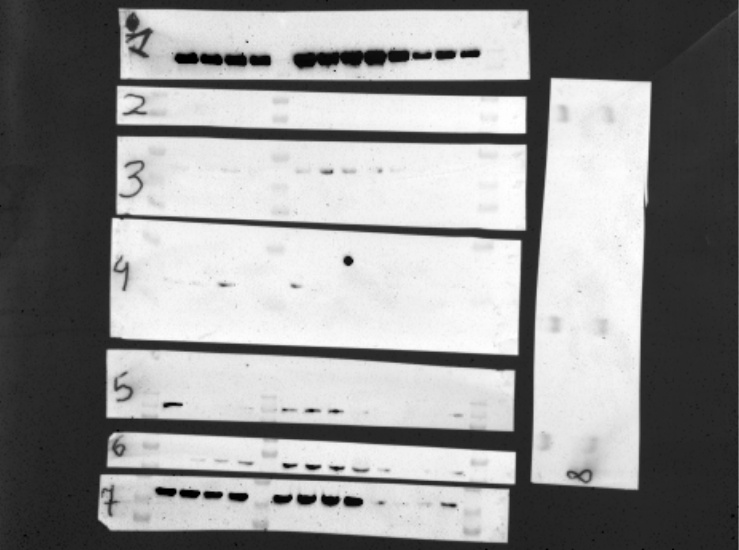

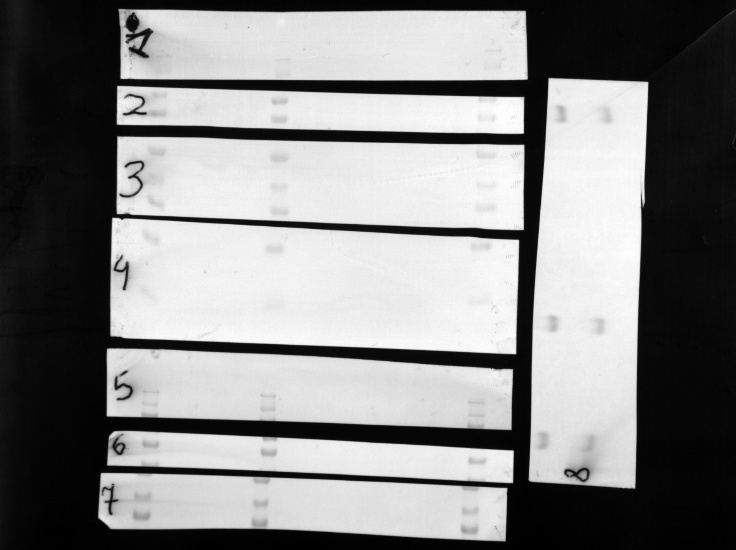


**260 kDa**

**140 kDa**

**100 kDa**

**70 kDa**

**40 kDa**

**50 kDa**

**35 kDa**

Gel with protein analyzed Later exposure time

p70 S6 Kinase

R6

**W1B 11.5 µM**

**Control LN229**

**W1B 23 µM**

**W1B 35 µM**

**W1B 11.5 µM**

**Control LN229**

**W1B 23 µM**

**W1B 35 µM**


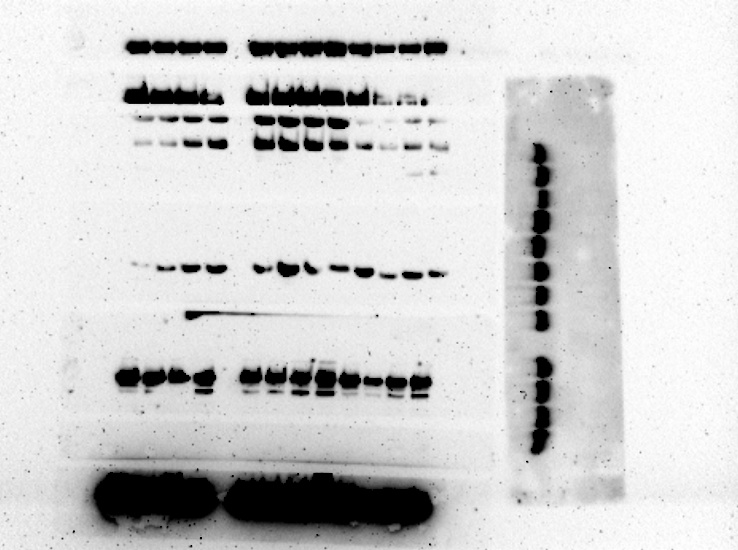

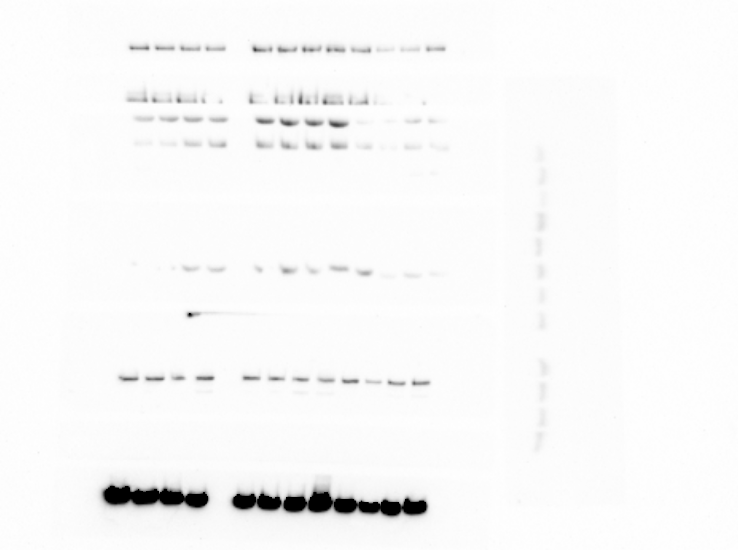


RAS R7

RAS R4-5

GAPDH

GAPDH

cyclophilin β

cyclophilin β

p70 S6 Kinase

R4-5

EGFR R15

EGFR R8-9

**W1B 11.5 µM**

**Control LN229 HG**

**W1B 23 µM**

**W1B 35 µM**

Gel with a molecular weight marker in CCD camera Merged images below

(colorimetric detection)


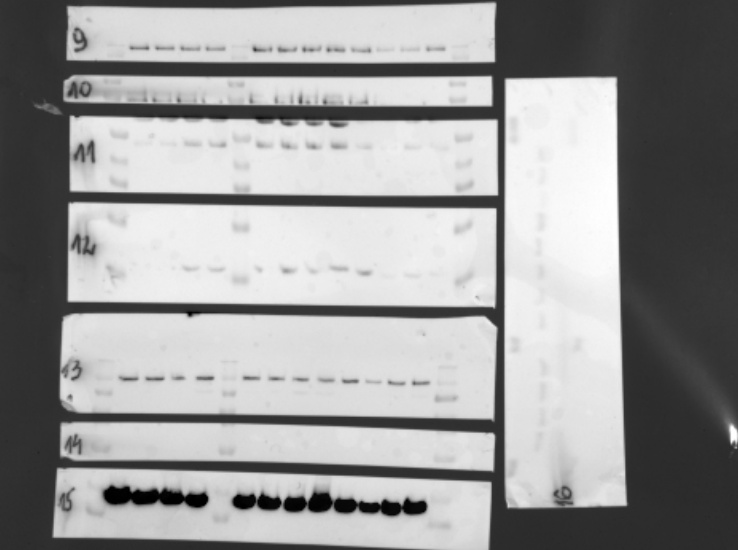

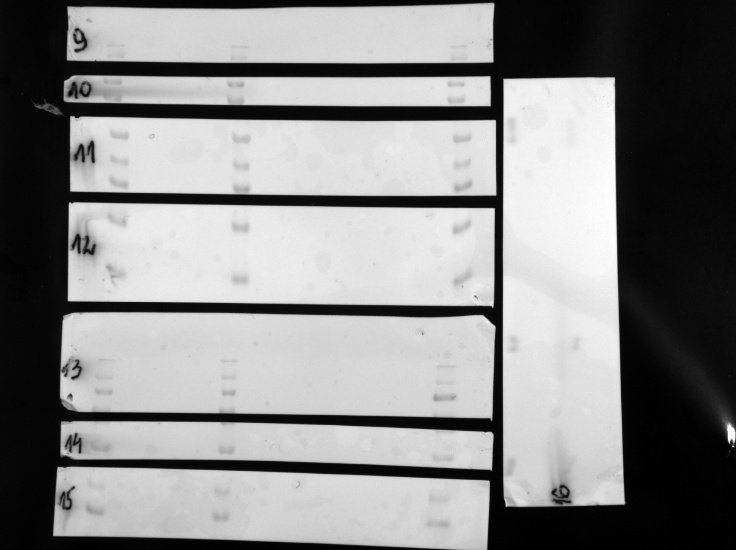


**260 kDa**

**140 kDa**

**100 kDa**

**70 kDa**

**25 kDa**

**40 kDa**

**50 kDa**

**35 kDa**

**15 kDa**

**260 kDa**

**140 kDa**

**100 kDa**

**70 kDa**

**25 kDa**

**40 kDa**

**50 kDa**

**35 kDa**

**15 kDa**

Gel with protein analyzed Later exposure time

vinculin


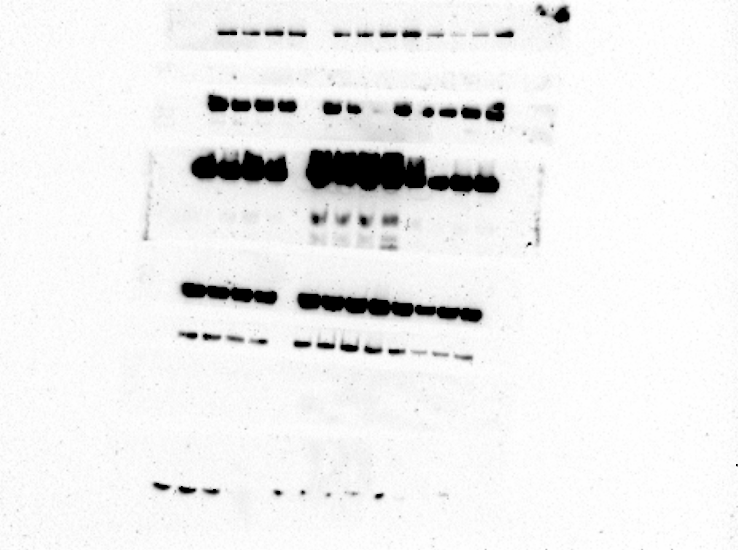

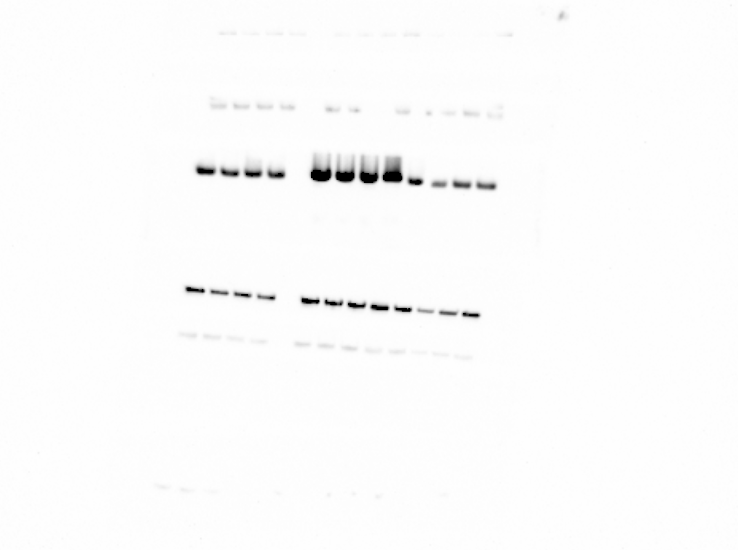


vinculin

p-70 S6 Ribosomal

Protein R7

p-70 S6 Ribosomal

Protein R4-5

**W1B 11.5 µM**

**Control LN229**

**W1B 23 µM**

**W1B 35 µM**

**W1B 11.5 µM**

**Control LN229 HG**

**W1B 23 µM**

**W1B 35 µM**

**W1B 11.5 µM**

**Control LN229**

**W1B 23 µM**

**W1B 35 µM**

Gel with a molecular weight marker in CCD camera Merged images below

(colorimetric detection)

**260 kDa**

**140 kDa**

**100 kDa**

**70 kDa**

**25 kDa**

**40 kDa**

**50 kDa**

**35 kDa**

**15 kDa**


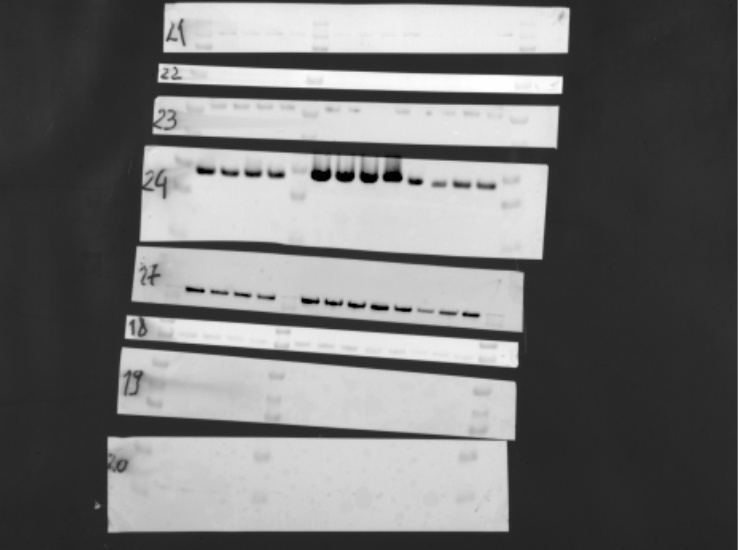

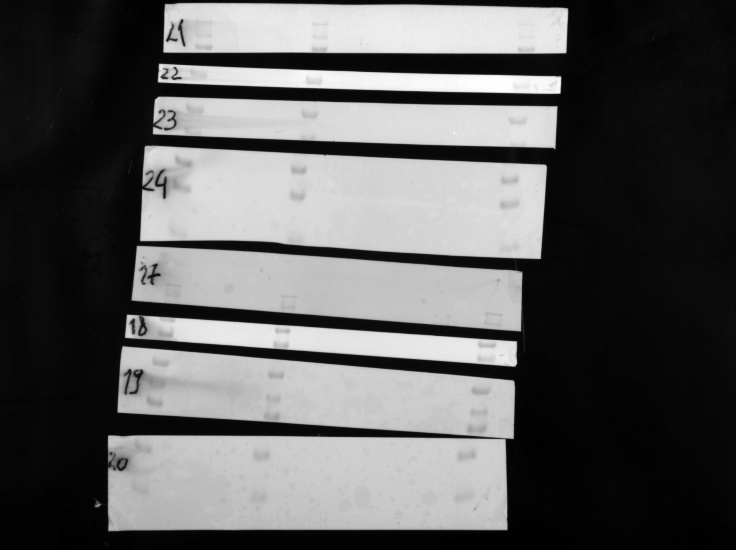


Gel with protein analyzed Later exposure time

**W1B 11.5 µM**

**Control LN229**

**W1B 23 µM**

**W1B 35 µM**

**W1B 11.5 µM**

**Control LN229**

**W1B 23 µM**

**W1B 35 µM**


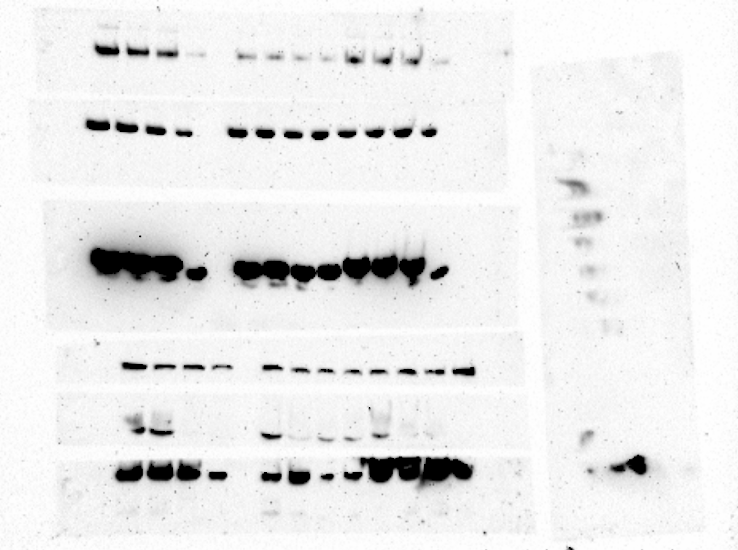

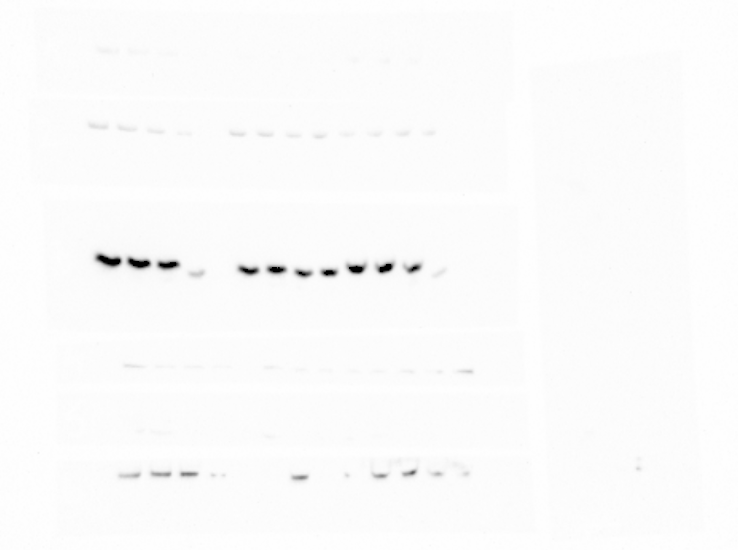


p-Akt R2-3

p-Akt R6

vinculin

vinculin

coffilin

coffilin

IGF1R R8

IGF1R R4-5

**W1B 11.5 µM**

**Control LN229 HG**

**W1B 23 µM**

**W1B 35 µM**

Gel with a molecular weight marker in CCD camera Merged images below


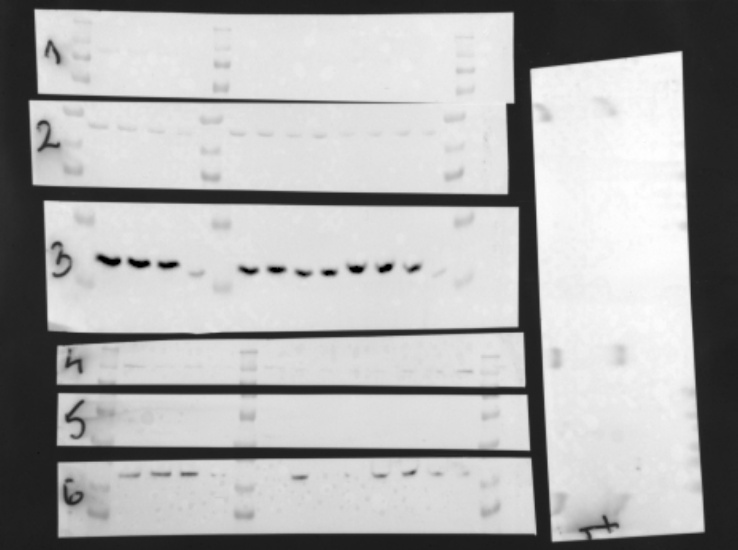
(colorimetric detection)


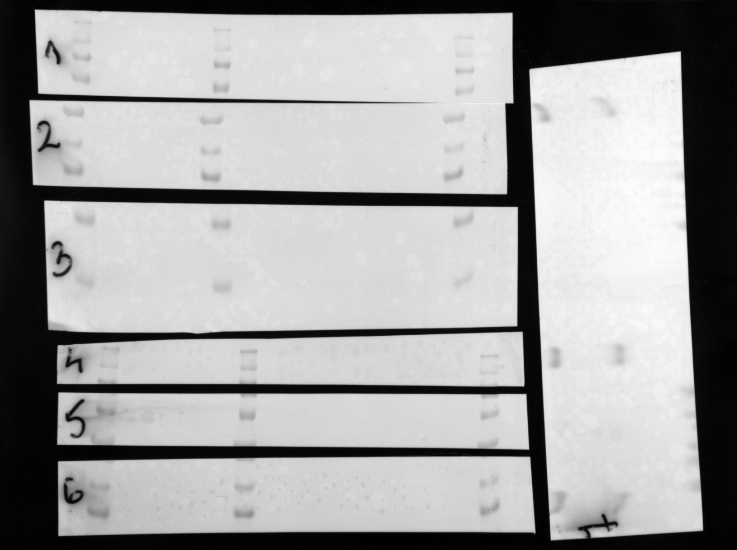


**260 kDa**

**140 kDa**

**100 kDa**

**70 kDa**

**25 kDa**

**40 kDa**

**50 kDa**

**35 kDa**

**15 kDa**

**260 kDa**

**140 kDa**

**100 kDa**

**70 kDa**

**25 kDa**

**40 kDa**

**50 kDa**

**35 kDa**

**15 kDa**

Gel with protein analyzed


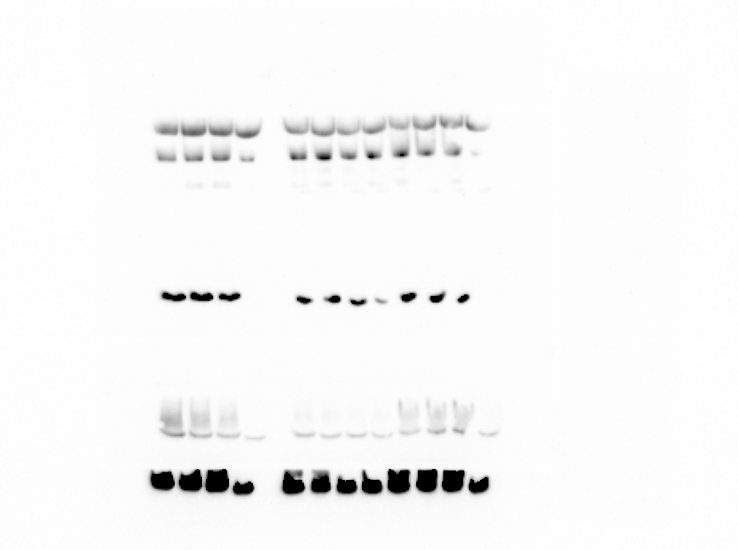


Akt R7

Akt R2-3

GAPDH

GAPDH

**W1B 11.5 µM**

**Control LN229 HG**

**W1B 23 µM**

**W1B 35 µM**

**W1B 11.5 µM**

**Control LN229**

**W1B 23 µM**

**W1B 35 µM**

**W1B 11.5 µM**

**Control LN229**

**W1B 23 µM**

**W1B 35 µM**

Gel with a molecular weight marker in CCD camera Merged images below

(colorimetric detection)


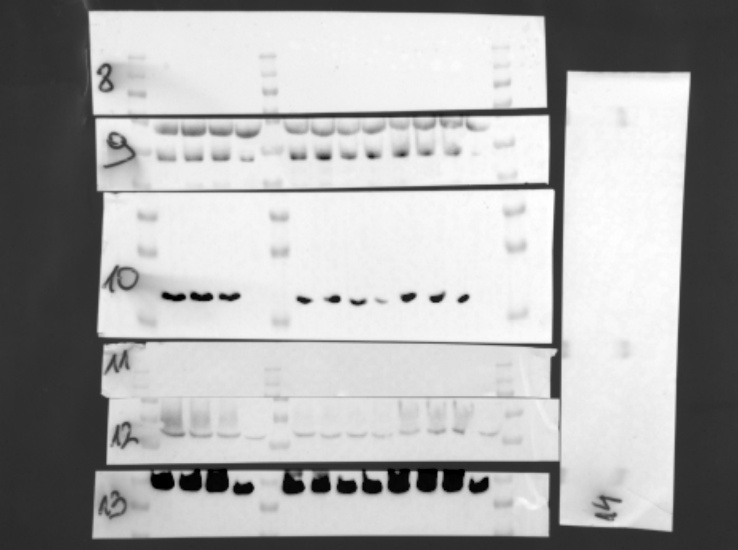

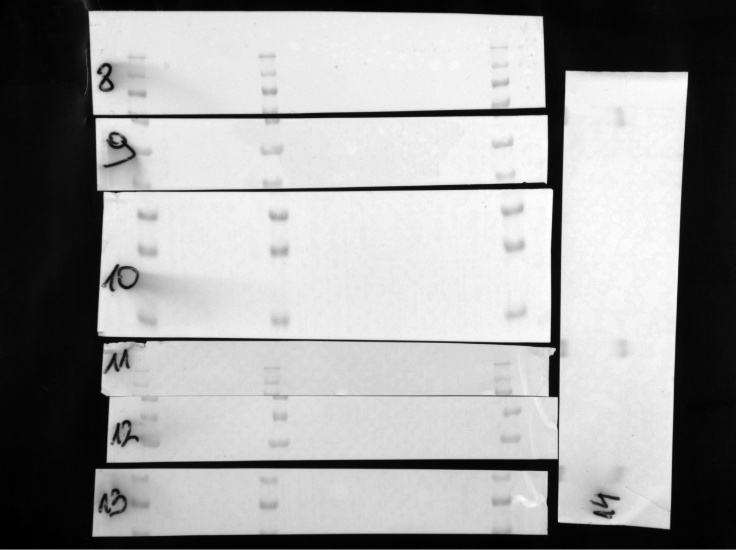


**260 kDa**

**140 kDa**

**100 kDa**

**70 kDa**

**25 kDa**

**40 kDa**

**50 kDa**

**35 kDa**

**15 kDa**

Gel with protein analyzed
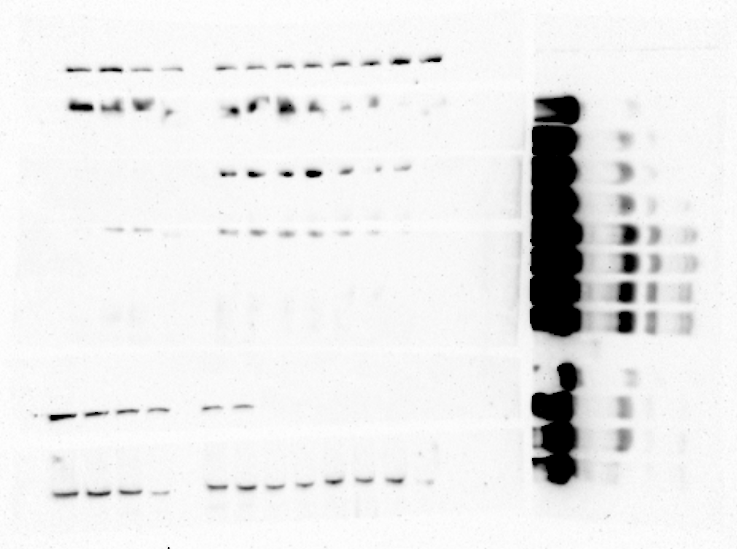


p-70 S6 Ribosomal

Protein R6

vinculin

**W1B 11.5 µM**

**Control LN229**

**W1B 23 µM**

**W1B 35 µM**

Gel with a molecular weight marker in CCD camera Merged images below

(colorimetric detection)


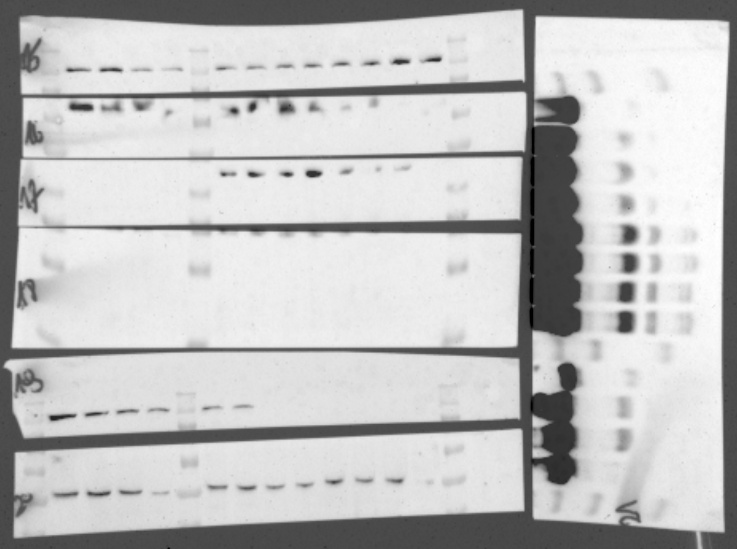

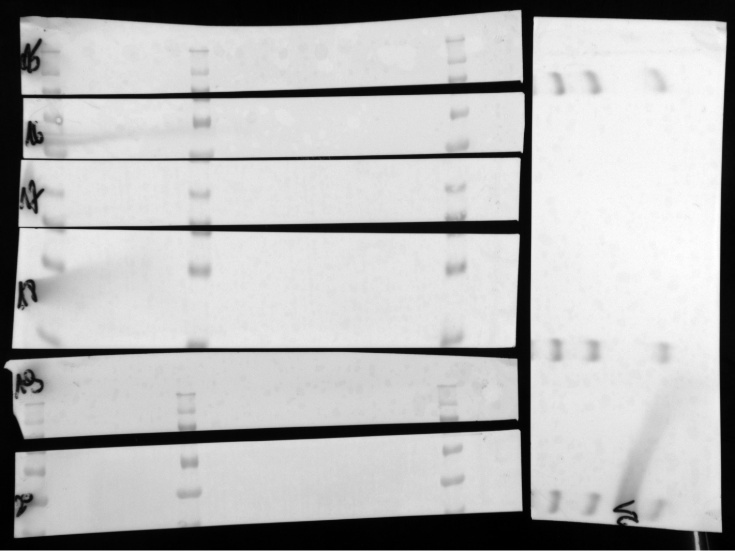


**260 kDa**

**140 kDa**

**100 kDa**

**70 kDa**

**25 kDa**

**40 kDa**

**50 kDa**

**35 kDa**

**15 kDa**

Gel with protein analyzed Later exposure time


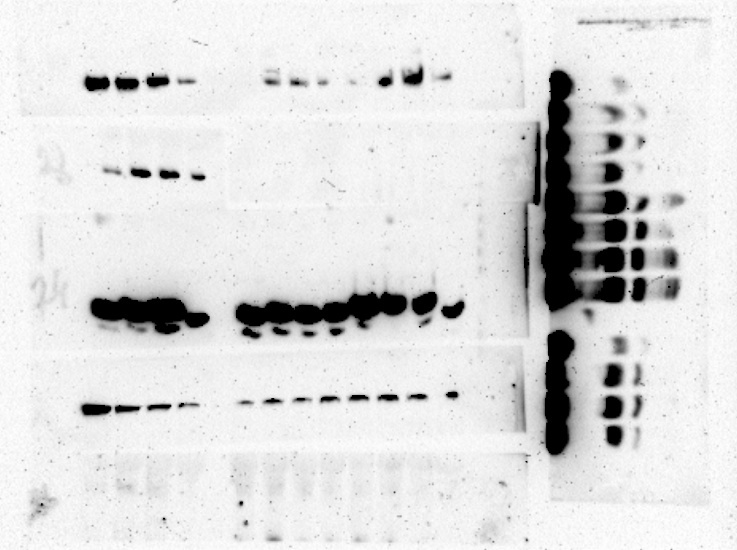

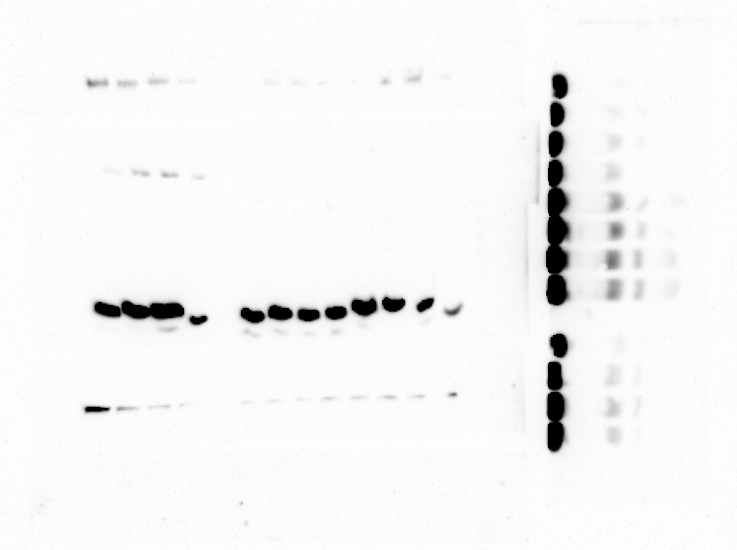


vinculin

vinculin

coffilin

coffilin

p-70 S6 Ribosomal

Protein R8

IGF1R R9

IGF1R R6

p-70 S6 Ribosomal

Protein R7-8

**W1B 11.5 µM**

**Control LN229**

**W1B 23 µM**

**W1B 35 µM**

**W1B 11.5 µM**

**Control LN229 HG**

**W1B 23 µM**

**W1B 35 µM**

**W1B 11.5 µM**

**Control LN229**

**W1B 23 µM**

**W1B 35 µM**

Gel with a molecular weight marker in CCD camera Merged images below

(colorimetric detection)


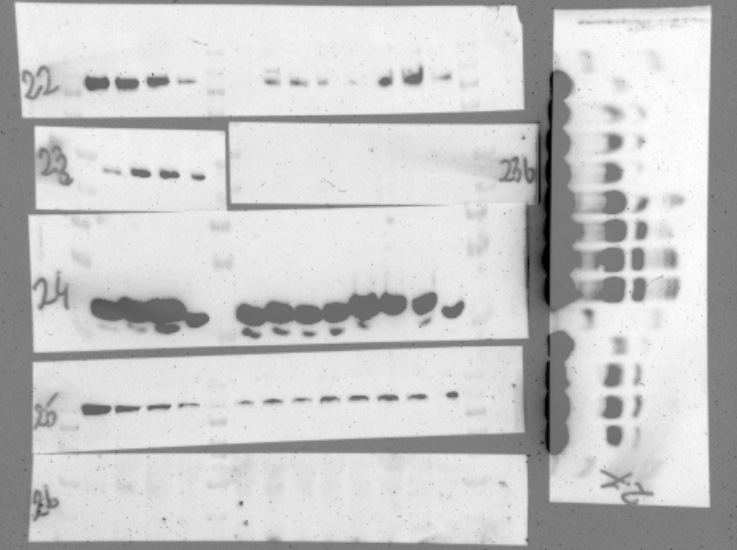

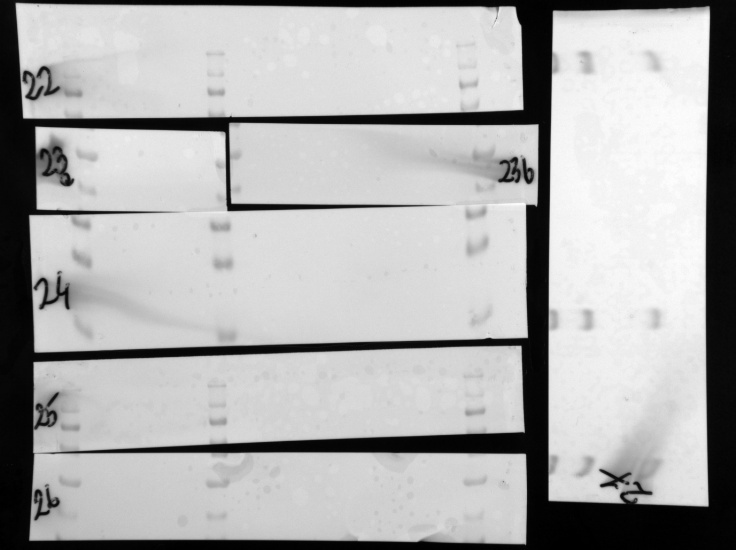


**260 kDa**

**140 kDa**

**100 kDa**

**70 kDa**

**25 kDa**

**40 kDa**

**50 kDa**

**35 kDa**

**15 kDa**

**260 kDa**

**140 kDa**

**100 kDa**

**70 kDa**

**25 kDa**

**40 kDa**

**50 kDa**

**35 kDa**

**15 kDa**

Gel with protein analyzed


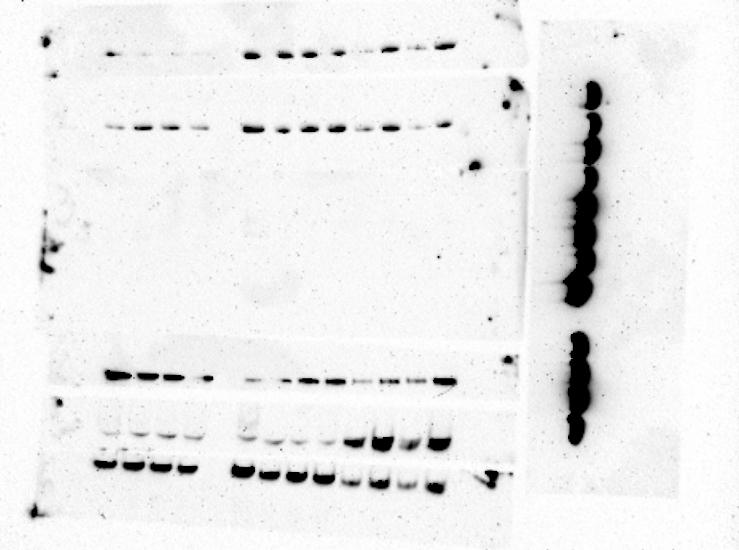


vinculin

vinculin

p-Akt R7

p-Akt R4

**W1B 11.5 µM**

**Control LN229**

**W1B 23 µM**

**W1B 35 µM**

**W1B 11.5 µM**

**Control LN229 HG**

**W1B 23 µM**

**W1B 35 µM**

Gel with a molecular weight marker in CCD camera Merged images below

(colorimetric detection)


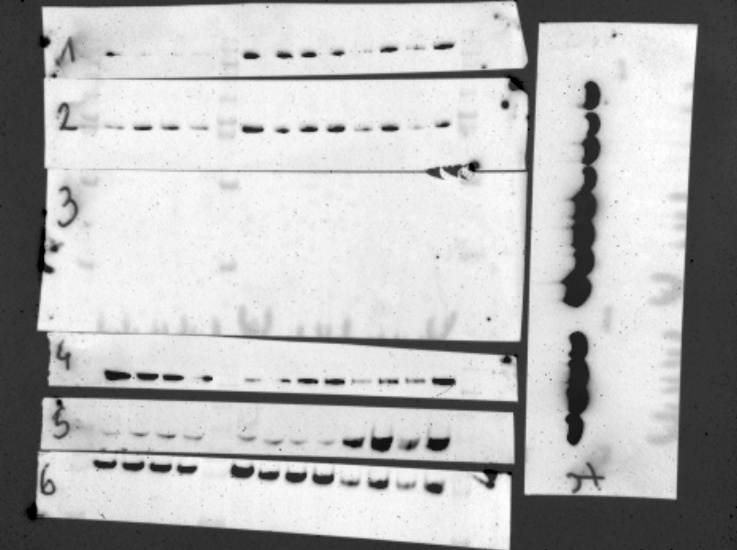

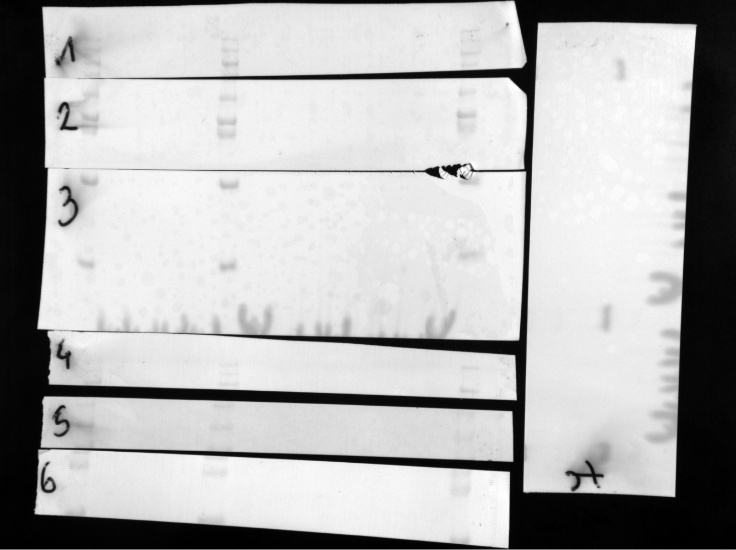


**260 kDa**

**160 kDa**

**110 kDa**

**80 kDa**

**30 kDa**

**50 kDa**

**60 kDa**

**40 kDa**

**15 kDa**

Gel with protein analyzed

**W1B 11.5 µM**

**Control LN229**

**W1B 23 µM**

**W1B 35 µM**


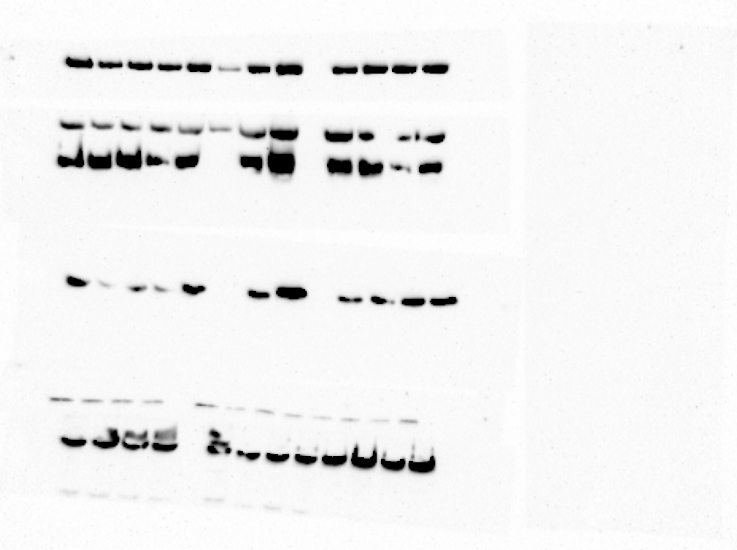


EGFR R16

EGFR R10

cofilin

cofilin

vinculin

vinculin

Akt R8

Akt R4

**W1B 11.5 µM**

**Control LN229 HG**

**W1B 23 µM**

**W1B 35 µM**

Gel with a molecular weight marker in CCD camera Merged images below


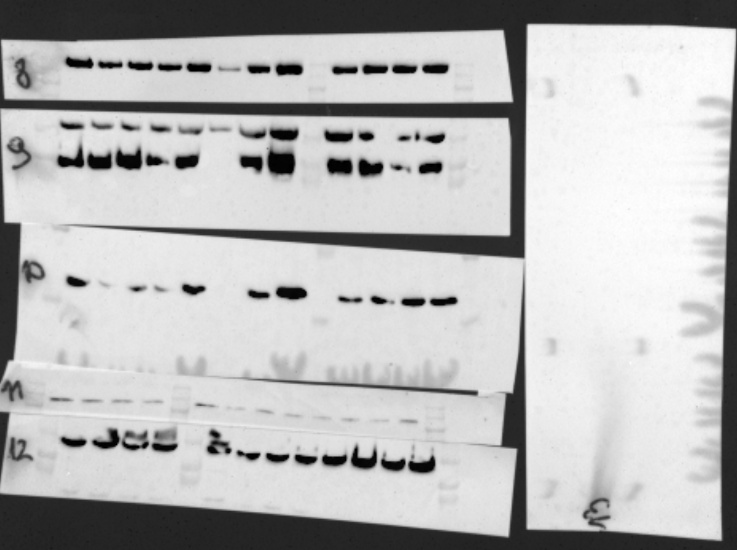
(colorimetric detection)


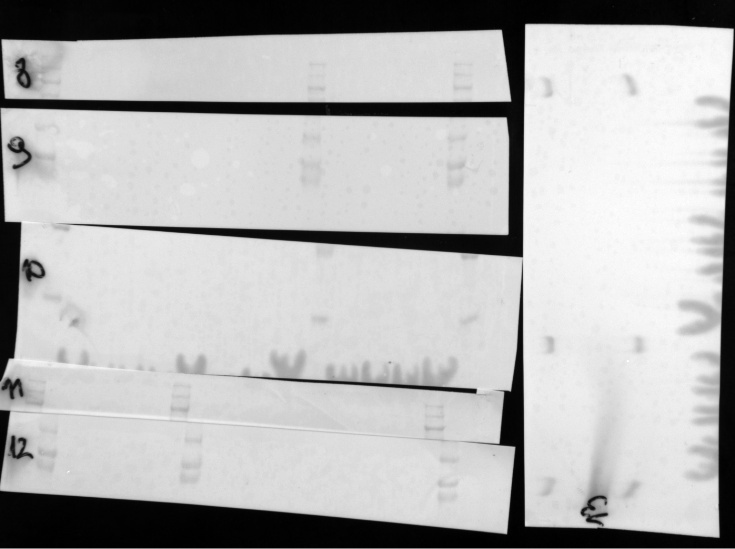


**260 kDa**

**160 kDa**

**110 kDa**

**80 kDa**

**30 kDa**

**50 kDa**

**60 kDa**

**40 kDa**

**15 kDa**

**260 kDa**

**160 kDa**

**110 kDa**

**80 kDa**

**30 kDa**

**50 kDa**

**60 kDa**

**40 kDa**

**15 kDa**

Gel with protein analyzed

vinculin

**W1B 11.5 µM**

**Control LN229**

**W1B 23 µM**

**W1B 35 µM**


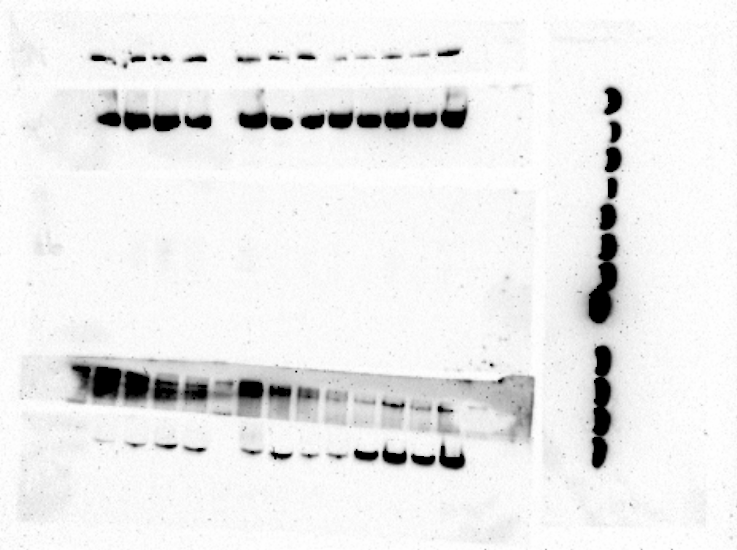


vinculin

p-Akt R8

p-Akt R5

**W1B 11.5 µM**

**Control LN229 HG**

**W1B 23 µM**

**W1B 35 µM**

Gel with a molecular weight marker in CCD camera Merged images below


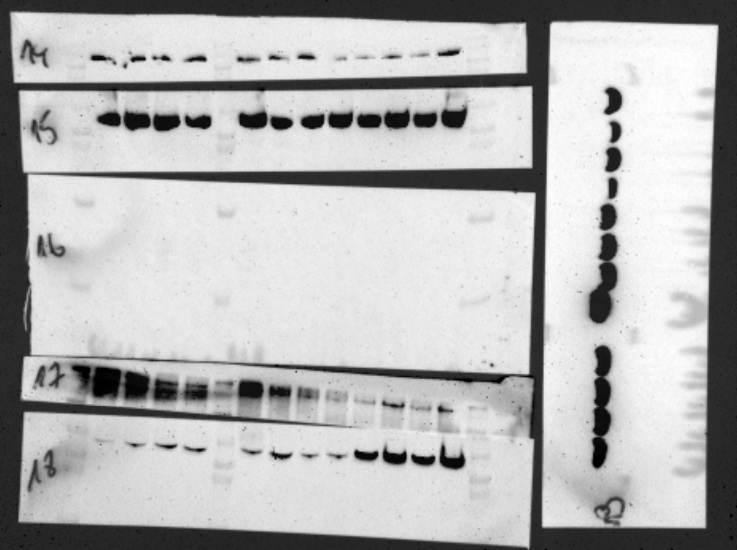
(colorimetric detection)


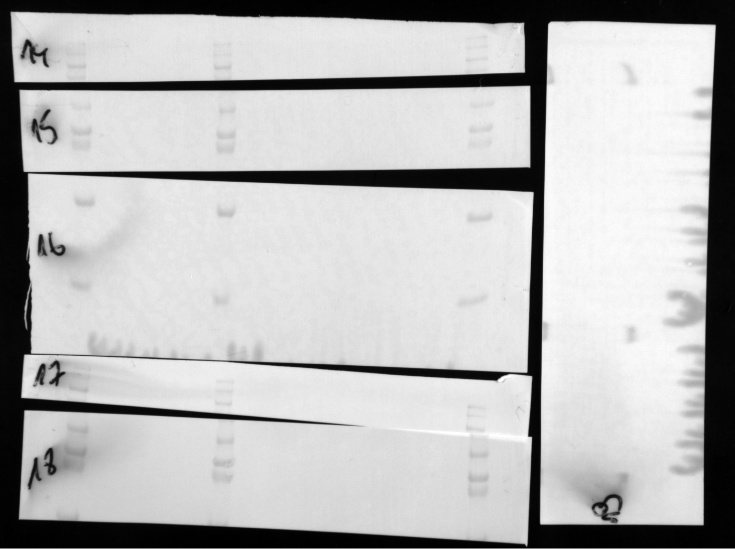


**260 kDa**

**160 kDa**

**110 kDa**

**80 kDa**

**30 kDa**

**50 kDa**

**60 kDa**

**40 kDa**

**15 kDa**

**Fig. S12.** The uncropped Western blots treated LN229 and LN229 HG cell lines with W1B compound in 3 different concentrations.

Gel with protein analyzed

**W1B 35 µM**

**Control LN229 HG**

**Dacomitinib 5.4µM**

**W1B 35 µM + Dacomitinib 5.4 µM**

**W1B 35 µM**

**Control LN229 HG**

**Dacomitinib 5.4µM**

**W1B 35 µM + Dacomitinib 5.4 µM**


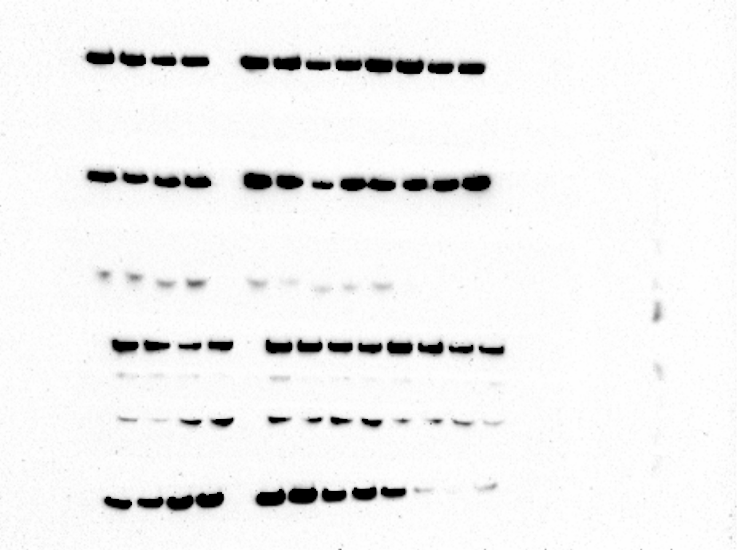


GADPH

GADPH

IGF1R R1-2

EGFR R4-5

EGFR R1-3

**W1B 35 µM**

**Control LN229 HG**

**Dacomitinib 5.4µM**

**W1B 35 µM + Dacomitinib 5.4 µM**

Gel with a molecular weight marker in CCD camera Merged images below

(colorimetric detection)


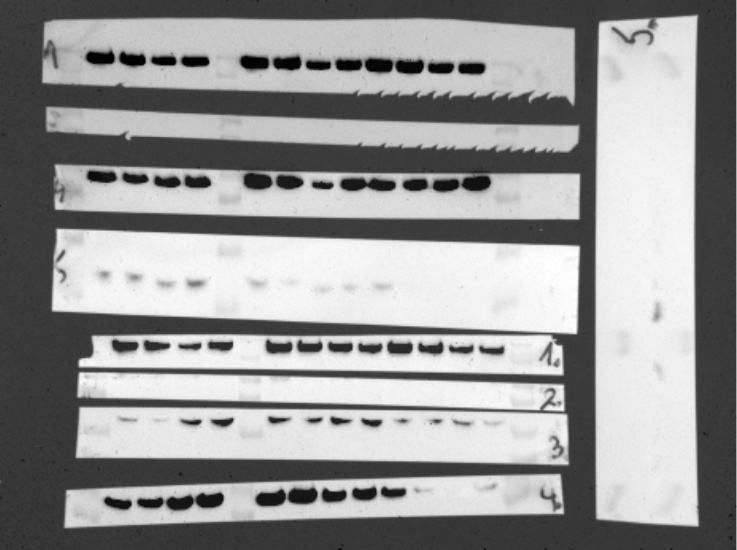

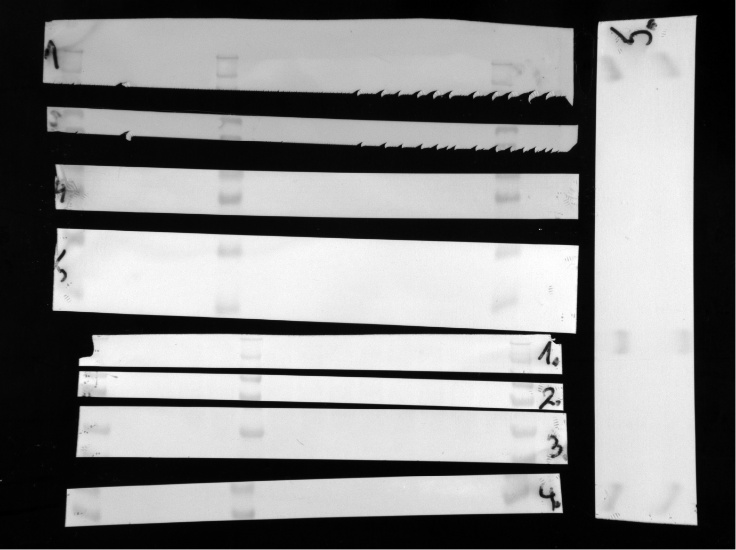


**260 kDa**

**140 kDa**

**100 kDa**

**70 kDa**

**25 kDa**

**40 kDa**

**50 kDa**

**35 kDa**

**15 kDa**

**260 kDa**

**140 kDa**

**100 kDa**

**70 kDa**

**25 kDa**

**40 kDa**

**50 kDa**

**35 kDa**

**15 kDa**

**W1B 35 µM**

**Control LN229 HG**

**Dacomitinib 5.4µM**

**W1B 35 µM + Dacomitinib 5.4 µM**

Gel with protein analyzed

**W1B 35 µM**

**Control LN229 HG**

**Dacomitinib 5.4µM**

**W1B 35 µM + Dacomitinib 5.4 µM**


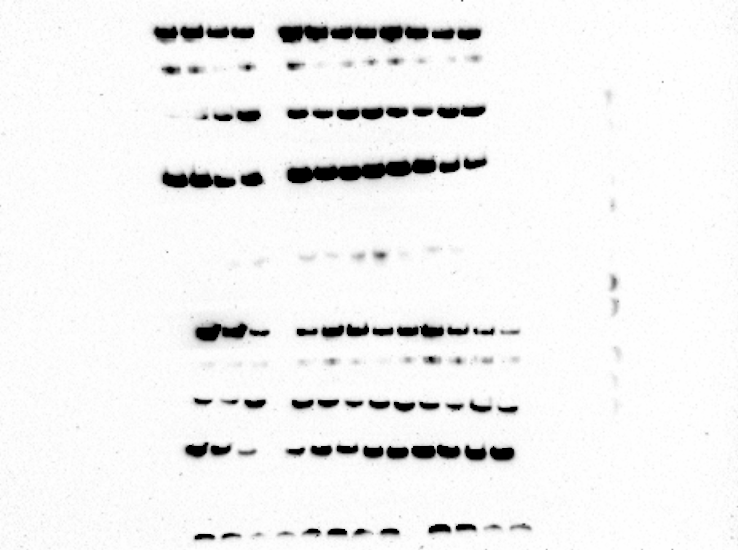


GADPH

GADPH

IGF1R R7-9

EGFR R10-12

IGF1R R3-6

EGFR R6-9

**W1B 35 µM**

**Control LN229 HG**

**Dacomitinib 5.4µM**

**W1B 35 µM + Dacomitinib 5.4 µM**

Gel with a molecular weight marker in CCD camera Merged images below

(colorimetric detection)


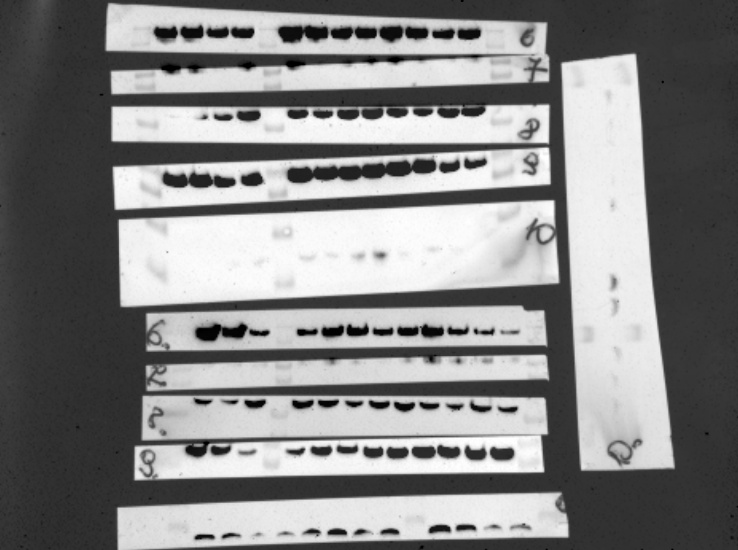

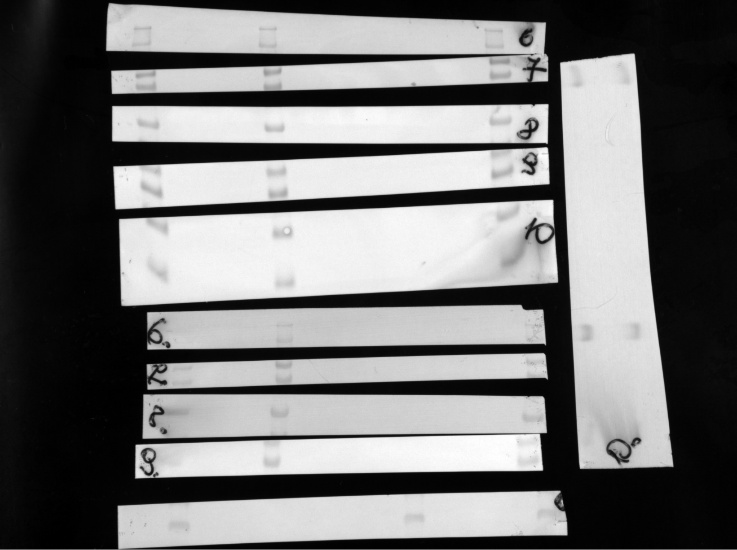


**50 kDa**

**260 kDa**

**140 kDa**

**100 kDa**

**70 kDa**

**25 kDa**

**40 kDa**

**50 kDa**

**35 kDa**

**15 kDa**

**260 kDa**

**140 kDa**

**100 kDa**

**70 kDa**

**25 kDa**

**40 kDa**

**50 kDa**

**35 kDa**

**15 kDa**

Gel with protein analyzed Later exposure time

GADPH

IGF1R R10

EGFR R13

**W1B 35 µM**

**Control LN229 HG**

**Dacomitinib 5.4µM**

**W1B 35 µM + Dacomitinib 5.4 µM**

Gel with a molecular weight marker in CCD camera Merged images below

(colorimetric detection)

**260 kDa**

**140 kDa**

**100 kDa**

**70 kDa**

**25 kDa**

**40 kDa**

**50 kDa**

**35 kDa**

**15 kDa**

Gel with protein analyzed Later exposure time

**W1B 35 µM**

**Control LN229 HG**

**Dacomitinib 5.4µM**

**W1B 35 µM + Dacomitinib 5.4 µM**

GADPH

GADPH

IGF1R R11

EGFR R15

EGFR R14

Gel with a molecular weight marker in CCD camera Merged images below

(colorimetric detection)

**260 kDa**

**140 kDa**

**100 kDa**

**70 kDa**

**25 kDa**

**40 kDa**

**50 kDa**

**35 kDa**

**15 kDa**

**40 kDa**

**35 kDa**

**260 kDa**

**140 kDa**

**100 kDa**

**70 kDa**

**25 kDa**

**40 kDa**

**50 kDa**

**35 kDa**

**15 kDa**

**Fig. S13.** The uncropped Western blots of LN229 HG cells treated with W1B compound, dacomitinib and their combination.
